# Aggregation-Induced Emission (AIE), Life and Health

**DOI:** 10.1021/acsnano.3c03925

**Published:** 2023-07-24

**Authors:** Haoran Wang, Qiyao Li, Parvej Alam, Haotian Bai, Vandana Bhalla, Martin R. Bryce, Mingyue Cao, Chao Chen, Sijie Chen, Xirui Chen, Yuncong Chen, Zhijun Chen, Dongfeng Dang, Dan Ding, Siyang Ding, Yanhong Duo, Meng Gao, Wei He, Xuewen He, Xuechuan Hong, Yuning Hong, Jing-Jing Hu, Rong Hu, Xiaolin Huang, Tony D. James, Xingyu Jiang, Gen-ichi Konishi, Ryan T. K. Kwok, Jacky W. Y. Lam, Chunbin Li, Haidong Li, Kai Li, Nan Li, Wei-Jian Li, Ying Li, Xing-Jie Liang, Yongye Liang, Bin Liu, Guozhen Liu, Xingang Liu, Xiaoding Lou, Xin-Yue Lou, Liang Luo, Paul R. McGonigal, Zong-Wan Mao, Guangle Niu, Tze Cin Owyong, Andrea Pucci, Jun Qian, Anjun Qin, Zijie Qiu, Andrey L. Rogach, Bo Situ, Kazuo Tanaka, Youhong Tang, Bingnan Wang, Dong Wang, Jianguo Wang, Wei Wang, Wen-Xiong Wang, Wen-Jin Wang, Xinyuan Wang, Yi-Feng Wang, Shuizhu Wu, Yifan Wu, Yonghua Xiong, Ruohan Xu, Chenxu Yan, Saisai Yan, Hai-Bo Yang, Lin-Lin Yang, Mingwang Yang, Ying-Wei Yang, Juyoung Yoon, Shuang-Quan Zang, Jiangjiang Zhang, Pengfei Zhang, Tianfu Zhang, Xin Zhang, Xin Zhang, Na Zhao, Zheng Zhao, Jie Zheng, Lei Zheng, Zheng Zheng, Ming-Qiang Zhu, Wei-Hong Zhu, Hang Zou, Ben Zhong Tang

**Affiliations:** † School of Science and Engineering, Shenzhen Institute of Aggregate Science and Technology, The Chinese University of Hong Kong, Shenzhen (CUHK-Shenzhen), Guangdong 518172, China; ‡ Department of Chemistry, Hong Kong Branch of Chinese National Engineering Research Center for Tissue Restoration and Reconstruction, Division of Life Science, State Key Laboratory of Molecular Neuroscience, Guangdong-Hong Kong-Macau Joint Laboratory of Optoelectronic and Magnetic Functional Materials, The Hong Kong University of Science and Technology, Clear Water Bay, Kowloon, Hong Kong SAR 999077, China; § Beijing National Laboratory for Molecular Sciences, Key Laboratory of Organic Solids, Institute of Chemistry, Chinese Academy of Sciences, Beijing 100190, China; ∥ State Key Laboratory of Crystal Materials, Shandong University, Jinan 250100, China; ⊥ Ming Wai Lau Centre for Reparative Medicine, Karolinska Institutet, Sha Tin, Hong Kong SAR 999077, China; # State Key Laboratory of Food Science and Resources, School of Food Science and Technology, Nanchang University, Nanchang 330047, China; ¶ State Key Laboratory of Coordination Chemistry, School of Chemistry and Chemical Engineering, Chemistry and Biomedicine Innovation Center (ChemBIC), Department of Cardiothoracic Surgery, Nanjing Drum Tower Hospital, Medical School, Nanjing University, Nanjing 210023, China; ⊕ Engineering Research Center of Advanced Wooden Materials and Key Laboratory of Bio-based Material Science and Technology of Ministry of Education, Northeast Forestry University, Harbin 150040, China; ⊖ School of Chemistry, Xi’an Jiaotong University, Xi’an 710049 China; ⊗ State Key Laboratory of Medicinal Chemical Biology, Key Laboratory of Bioactive Materials, Ministry of Education, and College of Life Sciences, Nankai University, Tianjin 300071, China; ○ Department of Biochemistry and Chemistry, La Trobe Institute for Molecular Science, La Trobe University, Melbourne, Victoria 3086, Australia; ● Department of Radiation Oncology, Shenzhen People’s Hospital (The Second Clinical Medical College, Jinan University, The First Affiliated Hospital, Southern University of Science and Technology), Shenzhen, Guangdong 518020, China; ▽ National Engineering Research Center for Tissue Restoration and Reconstruction, Key Laboratory of Biomedical Engineering of Guangdong Province, Key Laboratory of Biomedical Materials and Engineering of the Ministry of Education, Innovation Center for Tissue Restoration and Reconstruction, School of Materials Science and Engineering, South China University of Technology, Guangzhou 510006, China; ▼ The Key Lab of Health Chemistry and Molecular Diagnosis of Suzhou, College of Chemistry, Chemical Engineering and Materials Science, Soochow University, 199 Ren’ai Road, Suzhou 215123, China; △ State Key Laboratory of Virology, Department of Cardiology, Zhongnan Hospital of Wuhan University, School of Pharmaceutical Sciences, Wuhan University, Wuhan 430071, China; ▲ School of Chemistry and Chemical Engineering, University of South China, Hengyang 421001, China; □ Guangdong Provincial Key Laboratory of Advanced Biomaterials, Shenzhen Key Laboratory of Smart Healthcare Engineering, Department of Biomedical Engineering, Southern University of Science and Technology, No. 1088 Xueyuan Road, Nanshan District, Shenzhen, Guangdong 518055, China; ■ College of Chemistry and Chemical Engineering, Inner Mongolia Key Laboratory of Fine Organic Synthesis, Inner Mongolia University, Hohhot 010021, China; ◇ State Key Laboratory of Fine Chemicals, School of Bioengineering, Dalian University of Technology, 2 Linggong Road, Dalian 116024, China; ◆ College of Chemistry, Zhengzhou University, 100 Science Road, Zhengzhou 450001, China; α Key Laboratory of Macromolecular Science of Shaanxi Province, Key Laboratory of Applied Surface and Colloid Chemistry of Ministry of Education, School of Chemistry & Chemical Engineering, Shaanxi Normal University, Xi’an 710119, China; β Shanghai Key Laboratory of Green Chemistry and Chemical Processes & Chang-Kung Chuang Institute, East China Normal University, 3663 N. Zhongshan Road, Shanghai 200062, China; γ Innovation Research Center for AIE Pharmaceutical Biology, Guangzhou Municipal and Guangdong Provincial Key Laboratory of Molecular Target & Clinical Pharmacology, the NMPA and State Key Laboratory of Respiratory Disease, School of Pharmaceutical Sciences and the Fifth Affiliated Hospital, Guangzhou Medical University, Guangzhou 511436, China; δ CAS Key Laboratory for Biomedical Effects of Nanomaterials and Nanosafety, CAS Center for Excellence in Nanoscience, National Center for Nanoscience and Technology of China, Beijing 100190, China; ϵ School of Biomedical Engineering, Guangzhou Medical University, Guangzhou 511436, China; ζ Department of Materials Science and Engineering, Shenzhen Key Laboratory of Printed Organic Electronics, Southern University of Science and Technology, Shenzhen 518055, China; η Department of Chemical and Biomolecular Engineering, National University of Singapore, 4 Engineering Drive 4, Singapore 117585, Singapore; θ Ciechanover Institute of Precision and Regenerative Medicine, School of Medicine, The Chinese University of Hong Kong, Shenzhen (CUHK- Shenzhen), Guangdong 518172, China; ι State Key Laboratory of Biogeology and Environmental Geology, Engineering Research Center of Nano-Geomaterials of Ministry of Education, Faculty of Materials Science and Chemistry, China University of Geosciences, Wuhan 430074, China; κ International Joint Research Laboratory of Nano-Micro Architecture Chemistry, College of Chemistry, Jilin University, 2699 Qianjin Street, Changchun 130012, China; λ National Engineering Research Center for Nanomedicine, College of Life Science and Technology, Huazhong University of Science and Technology, Wuhan 430074, China; μ MOE Key Laboratory of Bioinorganic and Synthetic Chemistry, School of Chemistry, Sun Yat-Sen University, Guangzhou 510006, China; ν State Key Laboratory of Modern Optical Instrumentations, Centre for Optical and Electromagnetic Research, College of Optical Science and Engineering, International Research Center for Advanced Photonics, Zhejiang University, Hangzhou 310058, China; ξ State Key Laboratory of Luminescent Materials and Devices, Guangdong Provincial Key Laboratory of Luminescence from Molecular Aggregates, South China University of Technology, Guangzhou 510640, China; ο Department of Laboratory Medicine, Nanfang Hospital, Southern Medical University, Guangzhou 510515, China; π Institute for NanoScale Science and Technology, College of Science and Engineering, Flinders University, Bedford Park, South Australia 5042, Australia; ρ Center for AIE Research, College of Materials Science and Engineering, Shenzhen University, Shenzhen 518060, China; ϱ School of Energy and Environment and State Key Laboratory of Marine Pollution, City University of Hong Kong, Kowloon, Hong Kong SAR 999077, China; σ Central Laboratory of The Second Affiliated Hospital, School of Medicine, The Chinese University of Hong Kong, Shenzhen (CUHK- Shenzhen), & Longgang District People’s Hospital of Shenzhen, Guangdong 518172, China; τ State Key Laboratory of Luminescent Materials and Devices, Guangdong Provincial Key Laboratory of Luminescence from Molecular Aggregates, College of Materials Science and Engineering, South China University of Technology, Wushan Road 381, Guangzhou 510640, China; υ Key Laboratory for Advanced Materials and Joint International Research, Laboratory of Precision Chemistry and Molecular Engineering, Feringa Nobel Prize Scientist Joint Research Center, Institute of Fine Chemicals, Frontiers Science Center for Materiobiology and Dynamic Chemistry, School of Chemistry and Molecular Engineering, East China University of Science and Technology, Shanghai 200237, China; φ Department of Chemistry and Nanoscience, Ewha Womans University, Seoul 03760, Korea; χ Key Laboratory of Molecular Medicine and Biotherapy, the Ministry of Industry and Information Technology, School of Life Science, Beijing Institute of Technology, Beijing 100081, China; ψ Guangdong Key Laboratory of Nanomedicine, Shenzhen, Engineering Laboratory of Nanomedicine and Nanoformulations, CAS Key Lab for Health Informatics, Shenzhen Institute of Advanced Technology, Chinese Academy of Sciences, University Town of Shenzhen, 1068 Xueyuan Avenue, Shenzhen 518055, China; ω Department of Chemistry, Research Center for Industries of the Future, Westlake University, 600 Dunyu Road, Hangzhou, Zhejiang Province 310030, China; a Westlake Laboratory of Life Sciences and Biomedicine, 18 Shilongshan Road, Hangzhou, Zhejiang Province 310024, China; b Department of Chemical, Biomolecular, and Corrosion Engineering The University of Akron, Akron, Ohio 44325, United States; c School of Chemistry and Chemical Engineering, Hefei University of Technology, Hefei 230009, China; d Wuhan National Laboratory for Optoelectronics, School of Optical and Electronic Information, Huazhong University of Science and Technology, Wuhan 430074, China; e Clinical Translational Research Center of Aggregation-Induced Emission, School of Medicine, The Second Affiliated Hospital, School of Science and Engineering, The Chinese University of Hong Kong, Shenzhen (CUHK- Shenzhen), Guangdong 518172, China; f Department of Chemistry, Guru Nanak Dev University, Amritsar 143005, India; g Department of Chemical Science and Engineering, Tokyo Institute of Technology, O-okayama, Meguro-ku, Tokyo 152-8552, Japan; h Department of Chemistry, University of Bath, Bath BA2 7AY, United Kingdom; i Department of Materials Science and Engineering, City University of Hong Kong, Kowloon, Hong Kong SAR 999077, China; j Department of Chemistry, Durham University, South Road, Durham DH1 3LE, United Kingdom; k Department of Polymer Chemistry, Graduate School of Engineering, Kyoto University, Katsura, Nishikyo-ku, Kyoto 615-8510, Japan; l Department of Chemistry and Industrial Chemistry, University of Pisa, Via Moruzzi 13, Pisa 56124, Italy; m Department of Chemistry, University of York, Heslington, York YO10 5DD, United Kingdom

**Keywords:** aggregation-induced emission, luminescent material, bioimaging, phototheranostics, combined therapy, detection, monitoring, biomedical application, precision medicine

## Abstract

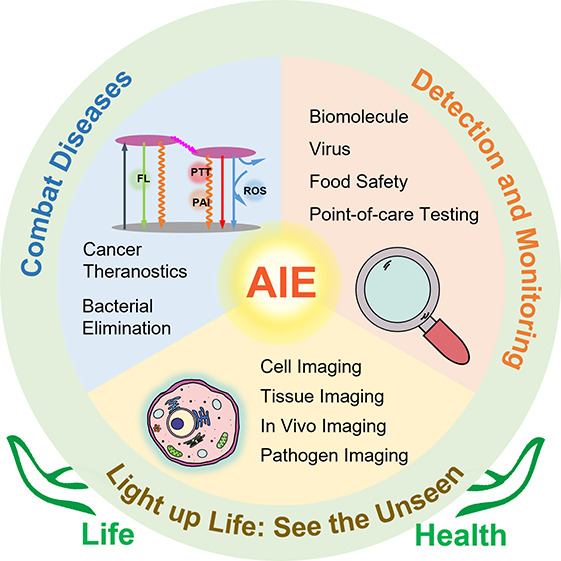

Light has profoundly impacted modern medicine and healthcare, with numerous luminescent agents and imaging techniques currently being used to assess health and treat diseases. As an emerging concept in luminescence, aggregation-induced emission (AIE) has shown great potential in biological applications due to its advantages in terms of brightness, biocompatibility, photostability, and positive correlation with concentration. This review provides a comprehensive summary of AIE luminogens applied in imaging of biological structure and dynamic physiological processes, disease diagnosis and treatment, and detection and monitoring of specific analytes, followed by representative works. Discussions on critical issues and perspectives on future directions are also included. This review aims to stimulate the interest of researchers from different fields, including chemistry, biology, materials science, medicine, etc., thus promoting the development of AIE in the fields of life and health.

## 1 Introduction

Health is of fundamental and utmost importance for human beings to enjoy a high-quality life.^1,2^ In the pursuit of this goal, significant endeavors have been made to understand human bodies and explore the mysteries of life, even since ancient times. From the humoral theory of Hippocrates in Greece to Zhongjing Zhang’s Treatise on Cold Damage Diseases (Shanghan Lun) in China, people in different areas have established systems and put forward different theories to guide the advancement of healthcare.^3,4^ The constant pursuit of good health has also boosted the underlying fundamental and applied science.^5^ The beginning of modern biology can be dated back to the invention of the microscope, which revealed the previously unknown world of microorganisms, laying the groundwork for cell theory.^6^ Since then, our ability to observe and understand physiological processes at the cellular level (and below) has steadily improved. Imaging the biogenic species and physiological processes is the first step to recognizing, monitoring, and regulating healthcare and diseases in living beings. With the advancement of imaging techniques and high-level requirements, modern imaging modalities have been developed, e.g., computed tomography (CT), magnetic resonance imaging (MRI), positron emission tomography (PET), ultrasound, etc.^7^ While these modalities have greatly improved medical diagnosis and treatment, their limitations are becoming apparent and cannot be ignored. CT and PET scans expose patients to hazardous ionizing radiation, which can increase the risk of cancer. MRI and PET imaging systems are comparatively expensive, while ultrasound imaging suffers from limited spatial resolution and tissue penetration.^8^ Compared with these methods, fluorescence imaging, with the merits of high sensitivity and selectivity, noninvasiveness, real-time and on-site responsiveness, and ease of application, has become an increasingly popular and expedient tool for direct visualization of biological structures and processes.^9^

Fluorescent agents, as the core of fluorescence imaging, have been developed for high-quality imaging, including organic dyes, conjugated polymer nanoparticles (NPs), semiconductor quantum dots (QDs), metal nanoclusters, up-conversion NPs, and fluorescent proteins.^10−22^ Among them, organic dyes and NPs have attracted immense research interest due to their advantageous features including precise molecular structures, controllable biocompatibility, tunable spectral characteristics, and versatile modification strategies.^23^ Traditional organic fluorescent molecules with rigid and coplanar structures, such as pyrene, are excellent emitters in dilute solutions. However, they can suffer from aggregation-caused quenching (ACQ) at high concentrations or in the solid state.^24,25^ The ACQ effect, early reported by Forster and Kasper, is attributed to strong π–π interactions formed in the aggregate state, which result in nonradiative decay of the excited state.^26,27^ Since most organic materials have hydrophobic structures and tend to aggregate in an aqueous physiological environment, the ACQ effect has greatly limited their applications in biological systems.

In 2001, a fluorescent molecule, 1-methyl-1,2,3,4,5-pentaphenylsilole, with a helical propeller structure was reported to be almost nonemissive in dilute solutions, but exhibited strong emission in the aggregate state.^28^ This photophysical behavior has been termed aggregation-induced emission (AIE). After more than 20 years of development in the field of AIE, the working mechanism of restriction of intramolecular motion (RIM) has been established and well-recognized.^29,30^ RIM can lead to conformational rigidification, thereby suppressing nonradiative decay pathways, e.g., vibronic coupling, conical intersection, and photochemical reactions, thus contributing to the enhanced luminescence process.^31−38^ The discovery and exploration of AIE has also greatly advanced the field of precision medicine and light-based healthcare.^39,40^ For fluorescence imaging, AIE luminogens (AIEgens) and their formed NPs are endowed with the inherent advantages of low background noise, fair photostability,^41−46^ large Stokes shift,^47−52^ biocompatibility,^53−56^ high brightness,^7,57−61^ and deeper tissue penetration and higher spatial resolution.^62,63^ In particular, the tetraphenylethylene (TPE) skeleton is the most versatile AIEgene because it can be designed to have various desired functions by introducing functional groups or π-extensions.^64^

**Scheme 1:**
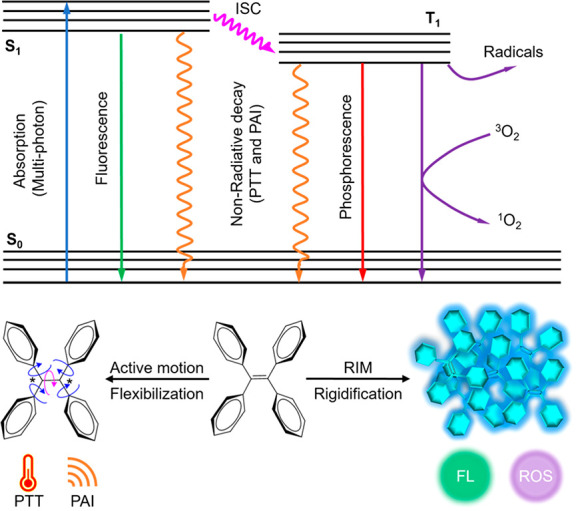
Schematic Illustration of AIEgens in Phototheranostics Based on the Jablonski Diagram

With the deepening of our understanding of the RIM mechanism, it is found that some AIEgens can work as highly efficient photosensitizers (PSs) in photodynamic therapy (PDT). Upon light irradiation, these AIEgens can produce reactive oxygen species (ROS), which are highly toxic to cancer cells and pathogens.^65,66^ Traditional PSs with rigid and coplanar structures usually suffer from diminished ROS sensitizing efficiency at high concentrations or when encapsulated in NPs. In contrast, AIE-active PSs, with stronger emission in the aqueous medium, exhibit enhanced ROS generation capacity upon aggregation, endowing them with excellent PDT characteristics suitable for practical applications.^67,68^ Except for restriction of molecular motion, reasonable promotion and utilization of solid-state molecular motion could work as a guideline for designing photothermal and photoacoustic systems.^69,70^ Following the molecular motion-facilitated nonradiative decay effect, the molecule is pumped to the singlet excited state and then can return to the ground state via nonradiative decay, converting the absorbed light into invisible forms of energy, such as heat.^71−73^ Thus, according to the Jablonski diagram, as shown in Scheme 1, when a molecule absorbs a photon and returns to the singlet excited state, there are three competitive pathways to release the excited-state energy and return to the ground state: (i) radiative decay referred to as fluorescence emission, (ii) intersystem crossing (ISC) transition from the singlet excited state to the lowest triplet state with the further release of energy through phosphorescence or transfer to oxygen to generate ROS, and (iii) nonradiative heat dissipation, which is desirable for photothermal therapy (PTT) and photoacoustic imaging (PAI). The balanced combination of the three excited-state energy dissipations in AIEgens could afford a versatile fluorescence imaging-guided phototheranostic system based on one single molecular species.^74−78^ Besides, efforts to combine PDT with other therapies, such as immunotherapy, chemotherapy and gene therapy, which could improve disease treatment efficiency and overcome the limitations of individual therapies, are showing promising results in clinical studies.^79−84^ Furthermore, being modified with different functional groups, AIEgens can be employed to capture analytes in biological systems.^85,86^ The intrinsic versatility of AIEgens enables fluorescence enhancement upon binding to their targets and differentiation of distinct biomolecular species upon binding.^87−89^ Pathological processes, including protein aggregation and cell apoptosis, can be monitored as well.

**Scheme 2:**
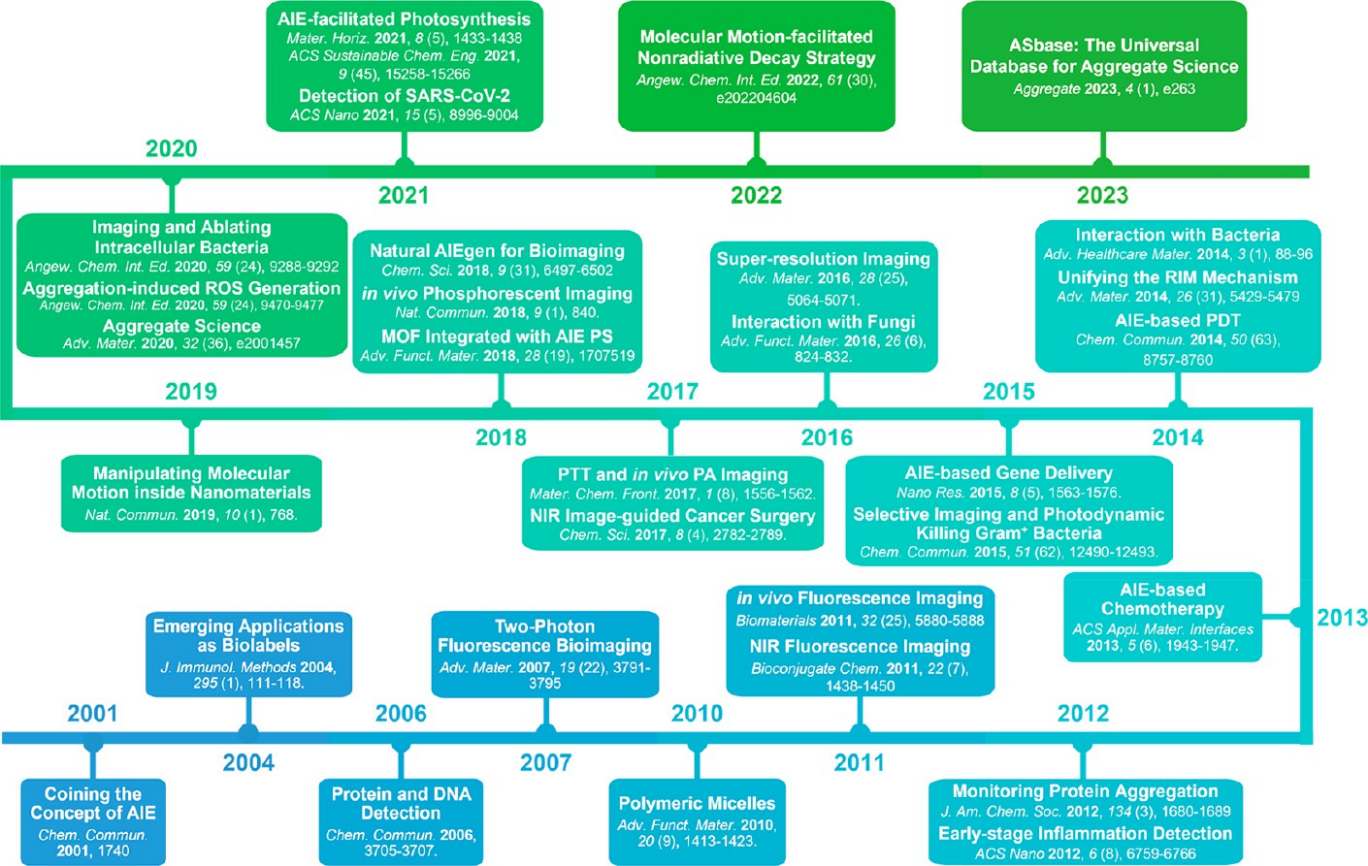
Timeline with the Critical Milestones in the Historical Development of AIE in Life Science and Health

Thanks to the persistent efforts and collaborative work of researchers around the globe, AIE has gained significant momentum and become an increasingly popular area (Scheme 2).^90^*Research trends and impact report on (Aggregation-Induced Emission) AIE, 2001–2021* released by Elsevier in 2022 indicated that the academic impact of AIE related to the terms “photothermotherapy”, “photodynamic therapy”, and “photosensitizer” was as high as 3.7 in terms of Field-Weighted Citation Impact. Moreover, AIE research in pharmacology, toxicology, pharmacy, environmental science, and medicine is gaining increasing attention. Undoubtedly, AIE has a significant impact on research and innovation in the fields of life science and health. In this review, the development of AIE for life and health is showcased in the following sections: imaging, diagnosis and treatment, and monitoring and detection. For each section, we will introduce and discuss these trends and progress, followed by presenting some representative research. A summary along with a discussion of current limitations, challenges, and opportunities will be provided at the end of this review.

## 2 Light up Life: See the Unseen

Fluorescence bioimaging is a sensitive, selective, and noninvasive method that has become a powerful tool in biological and pathological studies at the microscale. AIE has emerged as a promising approach for biological imaging, since it enables high-contrast imaging with low background noise, antiphotobleaching, and minimal toxicity. AIEgens are utilized to observe the refined structures of organelles or tissues and dynamic pathological processes such as deoxyribonucleic acid (DNA) aggregation, mitochondrial mitophagy, cell differentiation, apoptosis, drug/gene delivery, etc.^91−94^ AIE-based bioimaging has thus become a critical tool for advancing our understanding of biological processes and thereby facilitating the development of diagnostic and therapeutic approaches.

In this section, we will introduce the achievements in bioimaging by AIEgens with respect to the various levels from subcellular organelles and tissues to live animals and pathogens, respectively. In addition to commonly used fluorescence imaging, recently developed techniques such as super-resolution imaging, two-/three-photon imaging, near-infrared imaging, and afterglow imaging will also be included.

### 2.1 Cell Imaging

Living systems are highly complex and precisely regulated with the fundamental functional unit being the cell. Subcellular organelles are essential components of cells, with various organelles found in eukaryotic cells.^95^ Each organelle plays a unique role in supporting normal cell functions, such as the cell membrane for cell recognition and signal transmission, mitochondria for energy generation, lipid droplets (LDs) for lipid storage and metabolism, lysosomes for macromolecule degradation, and the endoplasmic reticulum (ER) for protein synthesis and folding.^96−100^ Studies have revealed that abnormal metabolic fluctuations within these subcellular organelles are highly associated with many severe diseases and disorders, including Alzheimer’s disease, fatty liver disease, and cancer.^101−103^ Therefore, visualizing subcellular structures and dynamic biological activities within specific organelles is essential for understanding, unveiling their functions, and illustrating how they contribute to health and disease.

#### 2.1.1 Static Structure Visualization

Imaging fine structures of biological specimens at scales ranging from the subcellular to tissue level is challenging. For most conventional probes, locally aggregated probe molecules suffer from a self-quenching effect, which is detrimental to achieving a high signal-to-background ratio. In contrast, probe aggregation is beneficial for fluorescent probes with AIE properties, which do not emit light in solution but fluoresce intensely upon binding to targets. TPEs and monomeric emitting AIEgens are particularly effective for imaging that requires noninvasiveness because they emit strongly only by adsorption to the target without aggregation of dyes.^104−106^ The development of AIE-active fluorescent probes over the past decade has expanded the color palette, elevated the imaging performance, and increased the variety of AIE-active probes for investigating biological structures.

Mitochondria are the energy factories of the cell, participating in diverse interconnected biological processes, including ATP generation, nucleotide biosynthesis, fatty acid oxidation, cell differentiation, and signal transduction. Benefiting from the negative membrane potential of mitochondria, cationic and lipophilic molecules prefer to accumulate in mitochondria through electronic interactions.^107^ Based on this principle, a series of mitochondria-targeting AIEgens was developed.^108,109^ A red emissive AIEgen (TPE-Ph-In) was constructed by conjugating a TPE group with an indolium group. Due to its cationic and AIE features, TPE-Ph-In stained the mitochondria with high specificity and high photostability. Moreover, TPE-Ph-In was highly sensitive to the mitochondrial membrane potential, which allowed facilitated the monitoring of mouse sperm activity.^110^ Zhao et al. systematically investigated the side chain effect for the mitochondria targeting ability of TPE functionalized pyridiniums and selected TPEPy-3 with suitable side chain length to specifically image the mitochondria.^111^ By fixing a cyano-pyridinium unit as an electron withdrawing group and varying aromatic moieties as electron donating groups, symmetric full-color AIEgens were successfully developed, which could be applied to multicolor imaging of mitochondria.^112^

**Figure 1:**
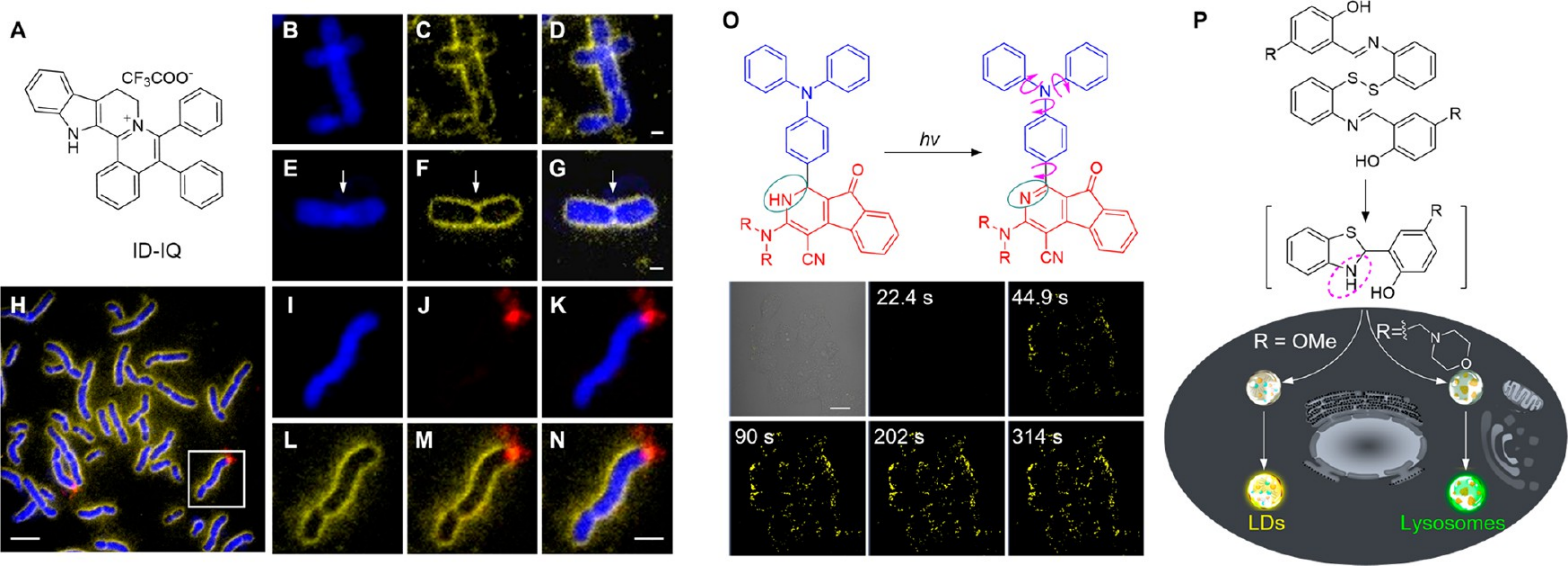
(A) Chemical structure of ID-IQ. (B–G) ID-IQ (yellow) labeled the boundary of chromosomes, which can facilitate segregation of overlapping and touching chromosomes (B–D). The centromere position (indicated by arrows) was clearly distinguished with the help of ID-IQ. These features cannot be easily achieved by Hoechst staining (blue). Scale bars: 1 μm. (H) Fluorescence image of lymphocyte chromosomes costained by ID-IQ (yellow), DAPI (blue), and the chromosome 4q telomere FISH probe (red). (I–N) Enlarged images from panel H. The telomere FISH probe labeled the end of the chromosome outlined by ID-IQ. Scale bars: 5 μm. Adapted with permission from ref (113). Copyright 2020 Wiley-VCH. (O) Photoactivatable dihydro-2-azafluorenones for LDs-specific imaging. Scale bar: 20 μm. Adapted with permission under a Creative Commons Attribution 3.0 Unported License from ref (114). Copyright 2017 The Royal Society of Chemistry. (P) *In situ* generated DH-HBT for photoactivatable LDs and lysosomes imaging. Adapted with permission under a Creative Commons Attribution 3.0 Unported License from ref (115). Copyright 2018 The Royal Society of Chemistry.

The chromosome is one of the most important subcellular structures. Chromosome abnormalities, either numerical or structural, can cause severe genetic disorders. The analysis of chromosomes and related abnormalities, named cytogenetic analysis, is widely used for the diagnosis of cancers and genetic diseases. However, in cytogenetic studies, it is often challenging to separate touching and overlapping chromosomes, precisely localize the centromere position, and identify a clear landmark to map gene loci on the chromosome. In 2020, Chen’s group reported a small-molecule fluorescent probe, ID-IQ, for chromosome periphery staining and cytogenetic studies (Figure 1A).^113^ ID-IQ is AIE active and exhibits a large Stokes shift (138 nm). Costaining ID-IQ with the DNA stain confirmed that ID-IQ specifically labeled the sheath-like structure on the surface of mitotic chromosomes known as the chromosome periphery. ID-IQ staining clearly outlined the chromosome morphology and highlighted the long arm, the short arm, and the centromere position of chromosomes—the latter is a constricted region that plays a key role in distributing the cells’ DNA during division. The high specificity and excellent contrast of ID-IQ enabled the precise identification of chromosome morphology and facile segregation of overlapping and touching chromosomes (Figure 1B–1G). ID-IQ staining was compatible with fluorescence in situ hybridization (FISH), one of the most widely used standard techniques in cytogenetic studies. Costaining ID-IQ with the FISH probe can assist in precisely locating a gene on the chromosome with high accuracy (Figure 1H–1N). This AIE probe can help clinical cytogeneticists quickly identify the cytogenetic location and chromosomal abnormalities and may find clinical applications in chromosome periphery studies.

Gao et al. developed a series of photoactivatable LDs-specific probes based on dihydro-2-azafluorenones with different amine substituents, which could easily undergo photooxidative dehydrogenation reaction to afford aromatic 2-azafluorenones with AIE characteristics based on RIM and twisted intramolecular charge transfer (TICT) in the aggregate state (Figure 1O).^114^ Based on this dehydrogenation reaction, an excellent light-up enhancement of 265-fold was obtained for LDs-specific turn-on imaging in LDs-rich cancer cells. The colocalization experiments with lipid dye BODIPY493/503 Green verified the LDs-specific imaging ability of AIE-active 2-azafluorenones. Selective photoactivation for cancer cells could be achieved in a multicellular environment with an excellent photoactivation efficiency and high spatiotemporal resolution, which can also efficiently discriminate between lung cancer and normal cells based on the different expression levels of LDs. To avoid the potential interference of environmental light for the storage of photoactivatable probes, a strategy for *in situ* generation of photoactivatable AIE probes based on 2-(2-hydroxyphenyl)-benzothiazolines (DH-HBT) was proposed. The probe, can be quantitatively generated *in situ* from readily available disulfide and thiol substrates through tandem S–S bond reduction and an intramolecular cyclization reaction (Figure 1P).^115^ The generated DH-HBT could efficiently undergo a photooxidative dehydrogenation reaction to afford the AIE-active 2-hydroxyphenyl-benzothiazole (HBT) under one- or two-photon light irradiation, which can be used for substituent-controlled selective imaging of LDs and lysosomes with excellent spatiotemporal resolution. Based on their *in situ* generation and adjustable organelle-targeting ability, the photoactivatable DH-HBT probes are easy-to-use imaging tools for the evaluation of the biological functions of organelles.

**Figure 2:**
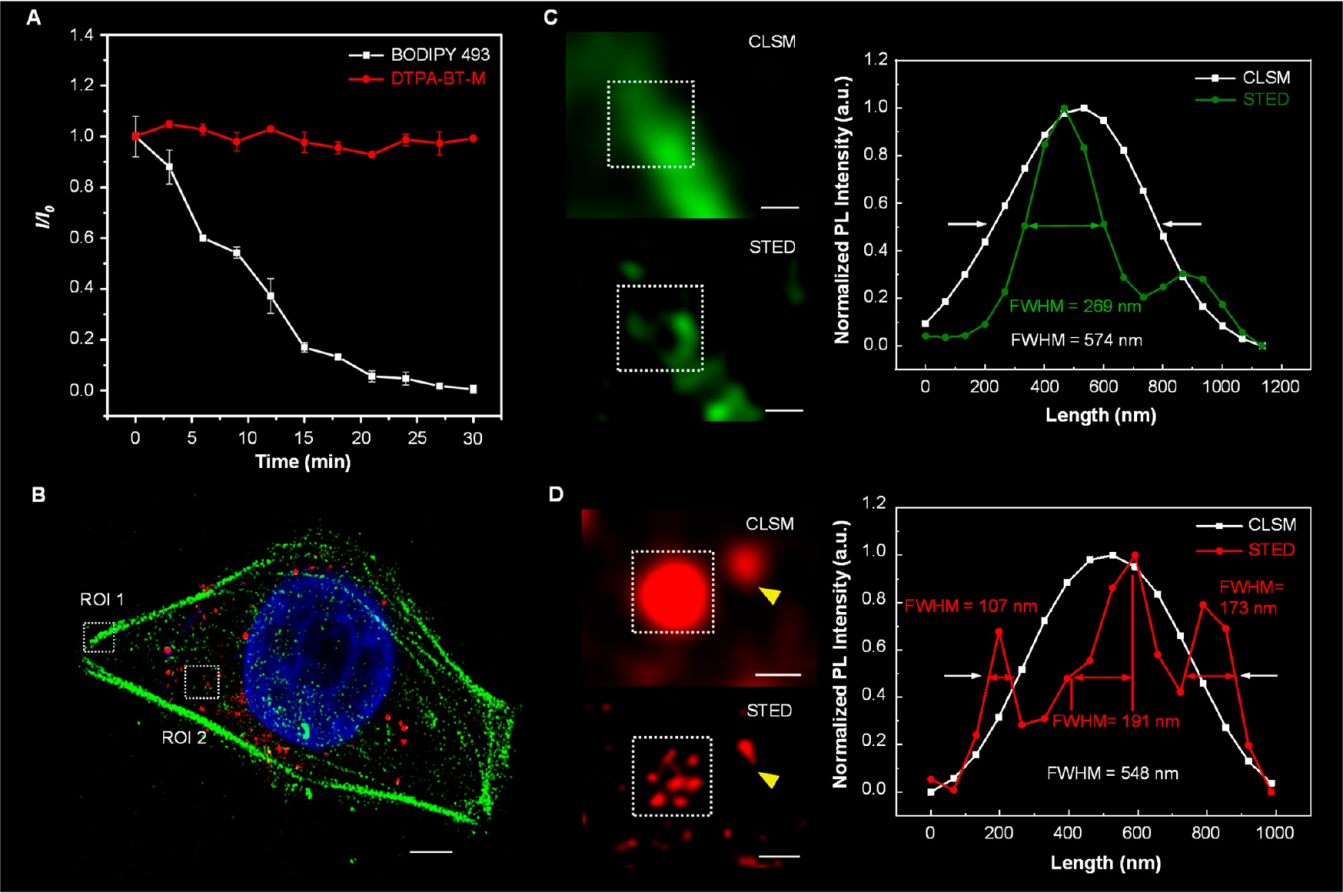
(A) Plots of relative fluorescence intensity (*I*/*I*_0_) for BODIPY 493/503 and DTPA-BT-M in HeLa cells by the continuous scanning via a depletion laser in STED nanoscopy over 30 min. Adapted with permission from ref (123). Copyright 2021 The Royal Society of Chemistry. (B) Fluorescence image of fixed HeLa cell costained with DAPI (blue), Alexa Fluor488-phalloidin (green), and DTPA-BT-F NCs (red) via STED nanoscopy. Scale bar: 5 μm. (C,D) Magnified fluorescence images and their corresponding PL intensities of (C) ROI 1 (Alexa Fluor488-phalloidin) and (D) ROI 2 (DTPA-BT-F NCs). Scale bars: 500 nm. Adapted with permission from ref (127). Copyright 2022 The Royal Society of Chemistry.

Among the fluorescence microscopies that have been widely applied for visualizing subcellular structures, stimulated emission depletion (STED) nanoscopy has been demonstrated to break the optical diffraction limit (200 nm) and thus achieve the imaging of biological objects at the nanoscale.^116−120^ Dang and co-workers developed several deep-red AIEgens with high brightness and excellent photostability. Red emission is highly desirable, as discussed in Section 2.3.1. These probes were eventually applied for organelle-specific imaging through STED nanoscopy.^121,122^ DTPA-BT-M with a symmetrical donor (D)-acceptor (A)-D architecture was developed in 2021.^123^ Due to the methoxy substitutions, an obvious bathochromic-shift absorption and red-shift emission occurred for DTPA-BT-M, then matching well the long-wavelength depleting beam of 775 nm in STED nanoscopy. Taking its large Stokes shift, high photoluminescence quantum yield (PLQY) and low biotoxicity into account, DTPA-BT-M was applied in STED nanoscopy for LDs-specific imaging. As shown in Figure 2A, after continuous scanning using a depletion laser, fluorescence signals of the commercial dye, BODIPY 493/503, were quickly quenched, whereas that of DTPA-BT-M remained unchanged, indicating its high photobleaching resistance in STED imaging. After incubating cells, only a slight decrease in diameter with minimally changed full width at half-maximum (FWHM) values observed for the LDs stained with BODIPY 493/503 under STED nanoscopy; however, the values for DTPA-BT-M changed from 370 to 95 nm. In addition, a D-A-typed AIEgen of DBTBT-4C8 was developed through the alkyl chain engineering strategy.^124^ In this case, an efficient intramolecular charge transfer (ICT) with a decreased π–π interaction was introduced. This resulted in the deep-red emission from 600 to 800 nm and high brightness with the absolute PLQY of 25% for DBTBT-4C8. Subsequently, DBTBT-4C8 was applied to prepare AIE NPs for the visualization of lysosomes in cells. Compared with conventional confocal microscopy (with a typical FWHM of ∼400 nm), superior resolution with a significantly decreased FWHM of 100 nm was achieved in lysosomes under STED mode. On the other hand, it is noteworthy that although several AIE NPs have been designed and applied for super-resolution imaging via STED nanoscopy, most of them are amorphous. This may still allow molecular motion to occur, which opens nonradiative channels.^125,126^ Therefore, to boost the fluorescence brightness for high-performance STED imaging, AIE nanocrystals (NCs) were developed.^127^ They possess more compact molecular stackings than their amorphous counterparts, thus leading to the desired optical properties, including deep-red emission with a PLQY value of 27.06%, an extinction coefficient of 13.1 × 10^3^ M^–1^·cm^–1^ as well as high brightness of 3.54 × 10^3^ M^–1^·cm^–1^. High photobleaching resistance and pH-stability were also observed in this case. Finally, DTPA-BT-F NCs were used for the super-resolution imaging of lysosomes (Figure 2B). As depicted, the imaging resolution for the cells stained with a commercial STED agent, Alexa Flour488-phalloidin, was only partly improved in STED mode, whereas in the case of DTPA-BT-F NCs, the FWHM significantly decreased from 548 to 107 nm (Figure 2C and 2D).

#### 2.1.2 Dynamic Process Visualization

Observing dynamic processes within and between cells is essential for gaining a comprehensive understanding of the complex biological systems that underlie all living organisms. Cells are dynamic entities that constantly engage in a wide range of processes such as cell division, migration, and differentiation, as well as the regulation of gene expression, metabolism, and signal transduction. By monitoring these processes at the cellular level, researchers can gain insights into the underlying mechanisms that govern cellular behavior and identify effective therapeutic strategies.

**Figure 3:**
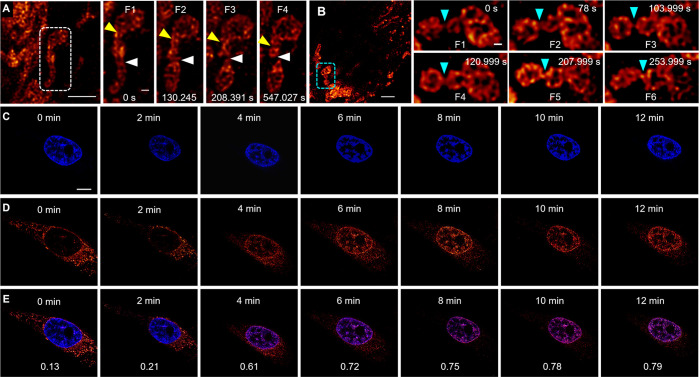
(A,B) Time-lapse STED imaging of mitochondrial dynamics (fission: yellow and white arrow; fusion: indigo arrow) in DTPAP-P labeled HeLa cells. The inset values are the precise time points. Scale bars: 2.5 μm. (C,D) Time-dependent fluorescence images of HeLa cells stained by (C) DAPI, (D) DTPAP-P in STED, and (E) their merged fields. The inset values in panel E are their corresponding Pearson’s correlation coefficients. Scale bar: 10 μm. Adapted with permission from ref (128). Copyright 2022 American Chemical Society.

Tunable organelle-specific imaging and their dynamic interplay on the nanometer scale are essential to studying biological behaviors. Dang et al. developed a cationic AIEgen DTPAP-P based on anion-π^+^ interactions. This provides cell status-dependent organelle targeting, enabling tunable organelle-specific imaging and dynamic tracking by a single AIEgen at ultrahigh resolution.^128^ A deep-red emission with excellent AIE features was observed for DTPAP-P, because of its twisted molecular structures and also the anion-π^+^ interactions here, which not only restrict the intramolecular motions but also prevent the strong π-π interactions. Afterward, mitochondria-specific imaging with a high Pearson’s correlation coefficient of 97% by costaining with MitoTracker Red was observed for DTPAP-P. In addition, a low saturation laser power value of 26 mW was also achieved using DTPAP-P, indicating the high depletion efficiency. For both fixed and live cells, DTPAP-P helped uncover the detailed structures of the mitochondria, providing high performance in STED imaging with a 6–7-fold increase in resolution. In addition, fission and fusion in mitochondria were clearly observed using the STED mode (Figure 3A and 3B). Interestingly, under light activation, a migration process appeared in fixed cells from mitochondria to nucleus. Time-dependent cell imaging was also recorded via STED nanoscopy (Figure 3C–3E). As illustrated, mitochondria-nucleus migration occurred when the irradiation time increased, resulting in the gradually enhanced emission in the nucleus until the migration was complete. These findings suggest that DTPAP-P can not only provide the detailed structures of organelles but also be used to further understand biological functions in cells.

**Figure 4:**
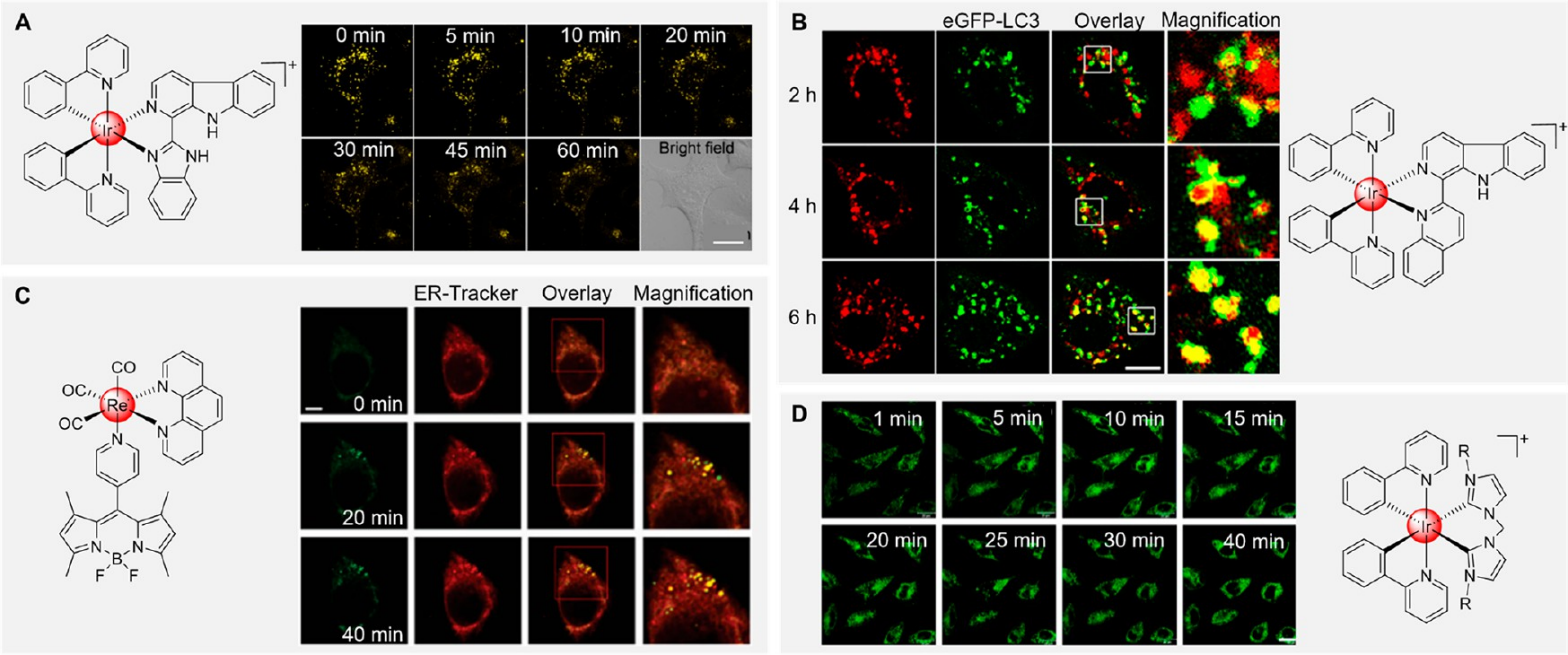
(A) The real-time monitoring of the lysosomal morphology in A549 cells. Scale bar: 10 μm. Adapted with permission under a Creative Commons Attribution 3.0 Unported License from ref (131). Copyright 2015 The Royal Society of Chemistry. (B) Visualization of autophagosomal-lysosomal fusion under two-photon excitation. Scale bar: 10 μm. Adapted with permission from ref (132). Copyright 2014 Wiley-VCH. (C) Dynamic monitoring of the autophagy of the endoplasmic reticulum. Scale bar: 5 μm. Adapted with permission under a Creative Commons CC BY License from ref (133). Copyright 2021 Oxford University Press. (D) Real-time tracking of mitochondria in HeLa cells for different time intervals. R = CH_2_CH_2_CH_2_CH_3_. Scale bar: 20 μm. Adapted with permission from ref (137). Copyright 2015 Elsevier.

The large amounts of substances required by cancer cells are predominantly obtained by phagocytosis, endocytosis, and micropinocytosis, which are delivered to lysosomes to generate nutrients through lysosomal degradation. Besides, the acidic surrounding of lysosomes could be used as a catalyst to activate the therapeutic effects of prodrugs.^129,130^ Taking advantage of the acidic environment of lysosomes, carboline alkaloids containing acid-activatable iridium complexes were developed. The protonation of the a carboline nitrogen atom could enhance the phosphorescence and ^1^O_2_ generation in tumor/lysosome-related acidic environments, which can be utilized to monitor lysosomal integrity (Figure 4A).^131^ With the help of the iridium complex in Figure 4B, the changes of lysosomal morphology during lysosome autophagy were monitored in real-time.^132^ Moreover, by introducing the commercial BODIPY unit into the metal rhenium complex, Mao’s group constructed an AIE-active complex for the environmental monitoring of the ER viscosity, and was used to evaluate changes in the ER viscosity during ER-to-lysosome-associated degradation (Figure 4C).^133^ By introducing a methylquinolin-1-ium group to the tripodal complex, a platinum complex with photoactivated lysosome-nucleus escape properties was designed.^134^ Mitochondria are biosynthetic, bioenergetic, and signaling organelles existing in almost all eukaryotic cells, and the state of them is closely related to tumorigenesis, tumor development, and tumor metastasis.^135,136^ A series of bis-*N*-heterocyclic carbene ligands containing positively charged iridium complexes show the capabilities of inducing and monitoring the morphological changes in mitochondria by initiating a cascade of events related to mitochondrial dysfunction (Figure 4D).^137^

A photoactivatable probe THTTVP containing a photosensitive tetrahydropyridine moiety was developed to facilitate the *in situ* monitoring of organelle interplay.^138^ After cellular uptake, this polarity-sensitive THTTVP probe first selectively accumulated in lysosomes and displayed weak emission, attributed to its ICT properties. Under white light irradiation, the probe could escape from lysosomes. One part of the THTTVP selectively accumulated in hydrophobic LDs with a turn-on green fluorescence, while other parts could undergo photo-oxidative dehydrogenation reaction and accumulate in cytomembrane and mitochondria with a turn-on red fluorescence. The interplay between LDs and mitochondria under ROS-induced oxidative stress can be monitored *in situ* and long-term, with significant fluorescence spectral difference. This photoactivatable tandem organelle imaging strategy is promising for the study of organelle interplay in various physiological processes.

Tracking cellular trafficking of metal ions with subsequent cascading subcellular effects is also of great importance, assisting our understanding on metal homeostasis, regulation, toxicity, as well as detoxification in the biological systems.^139^ Wang et al. recently employed two AIEgens (AMBPA for Zn^2+^; CF4 for Cu^+^) and showed that different metals (Zn and Cu) exhibited contrasting cellular behavior and functions, despite the fact that these were considered to be chemically similar and essential.^140^ When entering cells, the first target of the Zn element was the lysosome, whereas Cu only initially targeted the mitochondria. The initial active participation of lysosomes partially explains the homeosis of Zn in cellular systems. Beyond the handling ability of lysosomes, there was a burst of Zn to other subcellular organelles such as mitochondria, disrupting cellular homeostasis and causing cellular toxicity. In contrast to Zn, there was time-dependent Cu toxicity since it first targeted the mitochondria. Such differential targeting of subcellular organelles subsequently resulted in a cell functional change. The mitochondria membrane potential was much more sensitive to Cu exposure, whereas the lysosomal pH was more sensitive to Zn exposure.

DNA is essential for every form of life, carrying genetic instructions for the development, functioning, growth, and reproduction of all known organisms and many viruses. DNA damage triggered by endogenous and exogenous factors can affect biological processes and cause many diseases.^141−145^ Therefore, monitoring the state of DNA is important. To date, numerous fluorescent molecules have been deliberately designed to probe DNA-related activities, which can interact with DNA through electrostatic interactions, hydrophobic interactions, hydrogen bonding, or π-π stacking.^146^ Some of these molecules have been commercialized for nucleic acid binding and imaging, such as ethidium bromide, propidium iodide (PI), Hoechst 33258, and 4′,6-diamidino-2-phenylindole (DAPI).^147−150^ However, PI and other traditional DNA probes face the problems of stability, photobleaching, and the ACQ effect, which greatly limits their applications in imaging and detection. In sharp contrast, AIEgens are more stable and resistant to photobleaching, and more favorably, and are able to boost fluorescence in the aggregate state or upon binding with DNA, hence attracting great attention for detecting and illuminating DNA.^151−158^ An important feature of AIEgens is that their fluorescence signal can be turned on upon binding with DNA. With the increase in the concentration or the length of DNA, the fluorescence of AIEgens increases accordingly. Zhou’s group designed a derivative of p-phenylenediacetonitrile (FcPy) with pyridinium end groups, whose fluorescence was used to detect nucleic acids, including single-stranded DNA, double-stranded DNA, and ribonucleic acid (RNA).^159^ The increase in DNA sequence length led to an increase of the fluorescence of FcPy, given that a longer DNA sequence with more negative charges could attach more positively charged FcPy molecules through electrostatic interactions.

**Figure 5:**
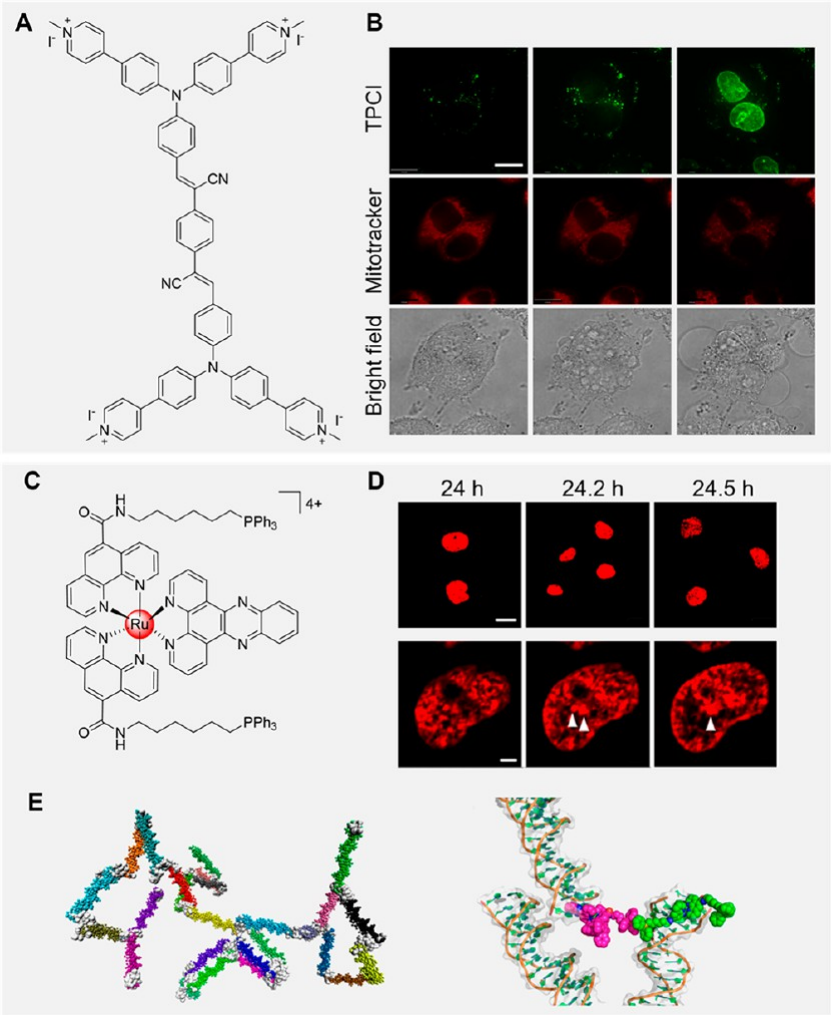
(A) Chemical structure of TPCI. (B) Time-lapse CLSM images of irradiated TPCI-pretreated living HeLa cells costained with MitoTracker. Time interval: 5 s. Scale bar: 13 μm. Adapted with permission from ref (160). Copyright 2019 Wiley-VCH. (C) Chemical structure of DNA light-switching ruthenium complex. (D) DNA aggregation in living cells visualized by confocal microscopy (top, scale bar: 20 μm) and super-resolution Airyscan (bottom, scale bar: 2 μm) at different time intervals. (E) Computer simulation of the induced DNA aggregation mechanism in all-atom MD simulation systems. Adapted with permission from ref (162). Copyright 2021 American Chemical Society.

Recently, researchers developed a dual-functional AIEgen, TPCI, with high DNA binding affinity (Figure 5A).^160^ The dual functionality of TPCI comprises: (i) the efficient ablation of cancer cells and (ii) reporting the anticancer effects in real time from the start of therapy. Determined by isothermal titration calorimetry, the binding constant of the DNA to TPCI was 5.68 × 10^8^ M ^–1^, higher than most DNA intercalators. Before light irradiation, there was no TPCI fluorescence in the nuclei of the HeLa cells. However, after laser irradiation, the fluorescence was turned on (Figure 5B). TPCI could translocate across the nuclear membrane and bind to nucleic acids in the chromatin, leading to fluorescence in the nuclei. In addition, studies on two other multicationic analogs with a similar triphenylamine core structure as TPCI, indicated that increasing the number of cationic branches could enhance the binding affinity of the AIEgens to DNA.^161^ Based on the photoswitching activity upon binding with DNA, a ruthenium complex with the ability to induce nuclear DNA aggregation (phase separation) was constructed (Figure 5C).^162^ The process of nuclear DNA aggregation was induced and traced (Figure 5D and 5E). Meanwhile, it was found that changes in the aggregate state of DNA could directly affect chromatin accessibility and result in the gene expression chaos of tumor cells, which provided a perspective for tumor therapy through modulation of tumor related gene expression.

G-quadruplex DNA is a special structure composed of poly guanine sequenced DNA. The amount of G-quadruplex DNA is significantly increased in a variety of tumor cells, suggesting that G-quadruplex may be linked with cancer progression.^163,164^ By introducing the AIE-active triphenylamine skeleton into cisplatin analogues, a platinum complex with the ability to induce G-quadruplex DNA aggregation was constructed, providing a structural basis for understanding this aggregation process.^165,166^ The binding features between AIEgens and DNA can further be employed to diagnose certain diseases involving abnormal DNA, such as DNA methylation and single nucleotide polymorphisms.^167−170^

**Figure 6:**
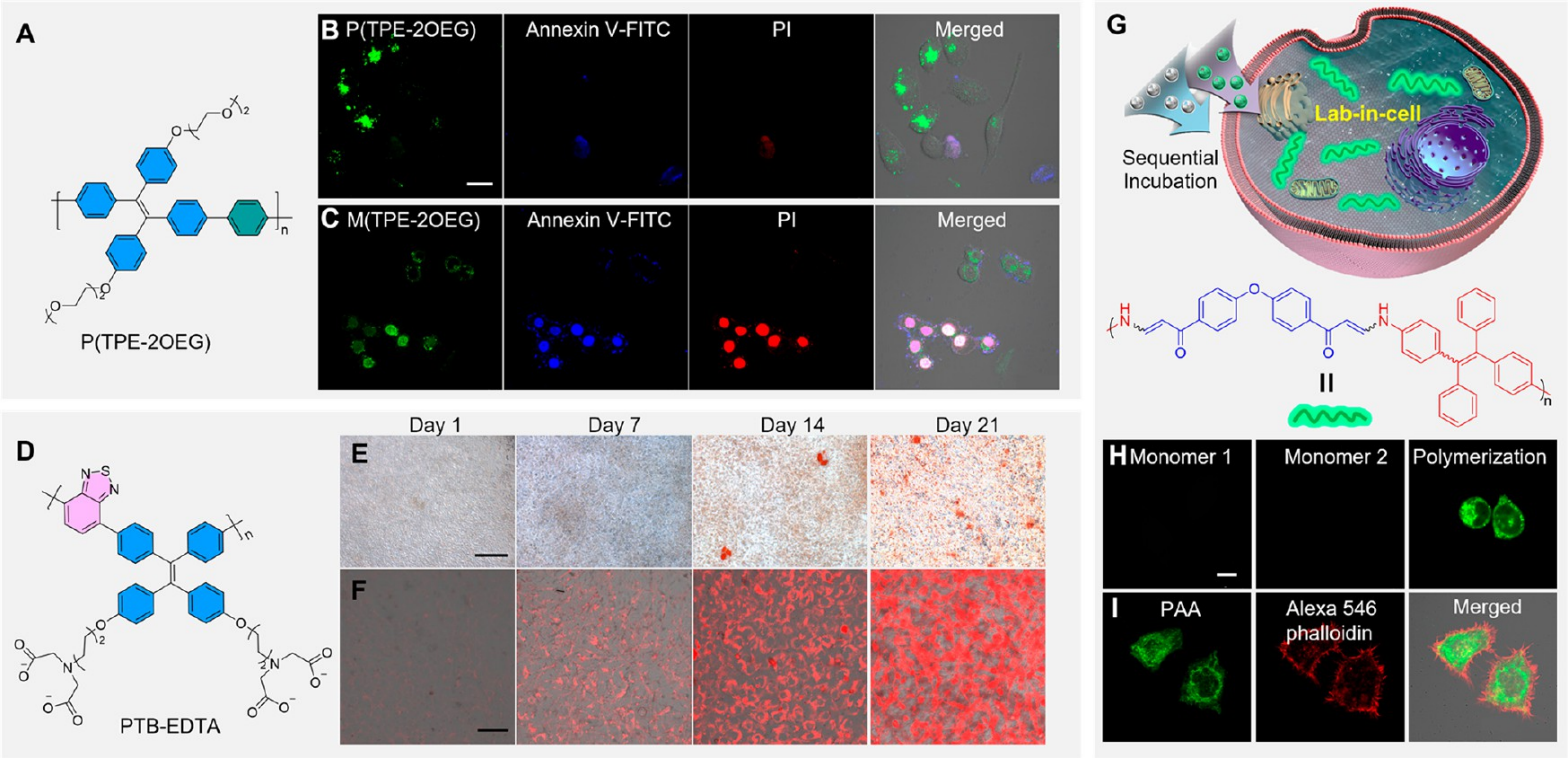
(A) Chemical structure of P(TPE-2OEG). (B,C) CLSM images of HeLa cells incubated with (B) P(TPE-2OEG) and (C) M(TPE-2OEG) after being treated with 500 μM H_2_O_2_ for 6 h and followed by staining with Annexin V-FITC and PI for 5 min. Scale bar: 20 μm. Adapted with permission from ref (171). Copyright 2020 Elsevier. (D) Chemical structure of PTB-EDTA. (E,F) Representative photos of (E) Alizarin Red S staining and (F) PTB-EDTA at indicated differentiation times. Scale bars: 100 μm. Adapted with permission from ref (172). Copyright 2020 Elsevier. (G) Illustration of the intracellular spontaneous amino-yne click polymerization and chemical structure of poly(β-aminoacrylate). (H) CLSM images of HeLa cells with different treatments. (I) CLSM images of HeLa cells with intracellular polymerization followed by Alexa 546 phalloidin labeling. Scale bar: 10 μm. Adapted with permission from ref (173). Copyright 2019 Springer Nature.

AIEgens also have great potential in visualizing cellular physiological activities, including apoptosis, autophagy, osteogenic differentiation, etc. Previous research demonstrated that the synergistic hydrophilicity and electric charge modulation could endow AIEgens with specific recognition properties. Therefore, it was rationalized that introduction of the electronegative oligo(ethylene glycol) side chains into the polymer P(TPE-2OEG) could reduce the nonspecific binding effect (Figure 6A). As shown in Figure 6B and 6C, good selectivity of P(TPE-2OEG) toward viable cells in the presence of dead and apoptotic ones was acquired, which benefited from the high molecular weight and synergistic effect and could not be realized by the related low-mass monomers.^171^ Furthermore, by the modification of the ethylenediaminetetraacetic acid (EDTA) moiety to the conjugated backbone, whose structure is shown in Figure 6D, the electronegativity of the side-chain was enhanced. Benefiting from the relatively intense binding ability with calcium ions (Ca^2+^), PTB-EDTA possessed high selectivity toward differentiated osteoblasts cells, which could not be realized by its monomer analog. Compared to the typical Alizarin Red S staining, the dynamic monitoring of the differentiation process could be observed by labeling with PTB-EDTA. Moreover, the sensitivity was much higher than that of the Alizarin Red S staining method by 7 days, which is highly desirable for related applications (Figure 6E and 6F).^172^ Taking advantage of the amino-yne click reaction, an intracellular polymerization was realized (Figure 6G). It was proved that the monomers alone could not light-up cells, while bright green fluorescence could be detected after the sequential incubation with the two monomers, realizing the turn-on imaging of HeLa cells (Figure 6H and 6I).^173^

Nanoparticle-based drug delivery systems (NDDSs), as a powerful platform for the controlled delivery of cargoes to the target site, have attracted considerable attention in biomedical fields.^174,175^ For the delivery process, NDDSs should be able to (i) accumulate at the target site with prolonged circulation time after administration, (ii) attach to the cell membrane and enter the cell, and (iii) then undergo controlled release of the loaded cargoes under different stimuli-responsive mechanisms at the subcellular organelle.^176−179^ Due to the high resolution and sensitivity, noninvasive fluorescent imaging is widely used as a first-line method for visualizing bio-nano interactions and dynamic transportation behavior.^180−184^ Using a typical AIEgen TPE, the dynamic delivery and release process of two representative cargoes, small molecule drug doxorubicin (DOX) and nucleic acid, was spatiotemporally visualized. DOX was bound to the surface of the TPE NPs via electrostatic interactions, providing self-indicating TPE/DOX (TD) NPs. When internalized into cells, TD NPs were first wrapped into lysosomes, and then, DOX was released from the surface of the TPE NPs under the acid microenvironment of lysosomes. Then, the TPE NPs were transported into the cytosol, and the released DOX was sorted into the nucleus.^185^ Then, a double-quenched nanoprodrug system was constructed.^186^ TPE and DOX were linked together via a pH-responsive hydrazone bond, thus forming THyD NPs by reprecipitation. THyD NPs were nonemissive due to the fluorescence resonance energy transfer between TPE and DOX. When internalized into cells, THyD NPs were sorted into lysosomes. The fluorescence signal of TPE and DOX was recovered along with breakage of the hydrazone bond under the acidic conditions of the microenvironment. Thus, the fluorescence “turn-on” process represents the spatiotemporal release kinetics.

Apart from the wide applications in mammalian cells, AIEgens also exhibit great potential for studies plants. Microalgae offer scope to help secure food supplies by synthesizing numerous compounds with health benefits. Lipid composition in microalgae can alter from saturation to unsaturation at the later phases of lipid accumulation, therefore, strict temporal and spatial controls on cell harvesting are critical for maximal lipid yield.^187^ Facile detection of the microalgal strains and the phases of optimum lipid production can advance algal lipid research in labor and cost-effective manners. Over the past few years, several high-quality, biocompatible fluorophores with AIE properties have been developed for lipid research.^188^ Recently, an AIE-active triphenylamine derivative, TPA-A was used to label and sort the *Chlorella vulgaris* cells with high lipid content.^189^ In the aggregate state, TPA-A exhibited a strong green fluorescence and could easily avoid the red fluorescence of chloroplasts. Due to the AIE properties and photostability of TPA-A, long-term monitoring of the LDs and chloroplasts during the growth period of *C. vulgaris* was realized and enabled determination of the optimum lipid harvesting time. Moreover, a rapid and wash-free strategy to visualize the LDs has also been reported in morphologically distinctive microalgae, *Euglena gracilis*, without a cell wall, and *Chlamydomonas reinhardtii*, with a carbohydrates-based cell wall utilizing AIEgens, DPAS, and 2-DPAN, respectively.^190,191^ The hydrophobicity of benzophenone allowed these nanoprobes to strongly target and accumulate in LDs that are also hydrophobic in nature. Additionally, neither of the AIEgens interfered with the red channel of chlorophyll autofluorescence, meaning that they are promising as a multicolor visualizing tool for microalgae.

### 2.2 Tissue Imaging

While the subcellular structure and cellular processes can be studied by *in vitro* imaging, *in situ* and direct imaging of intact tissues isolated from animals or humans can be used to obtain more intrinsic and accurate information. Different from cells added and cultured externally in an artificial medium, the interaction and status of immobilized cells in the tissue are more complex. In addition, tissue specimens are often thicker with compact objects, which causes severe autofluorescence and scattering during fluorescent imaging. Guided by the RIM mechanism, the intramolecular motions of AIE probes are restricted under the formation of aggregates, which activates the radiative pathway and strong emission. This mechanism has endowed AIE probes with high signal-to-noise ratio, photostability and brightness after specifically binding to the subcellular structure or protein in tissues, therefore exhibiting great promise in the field of tissue imaging.^192^

**Figure 7:**
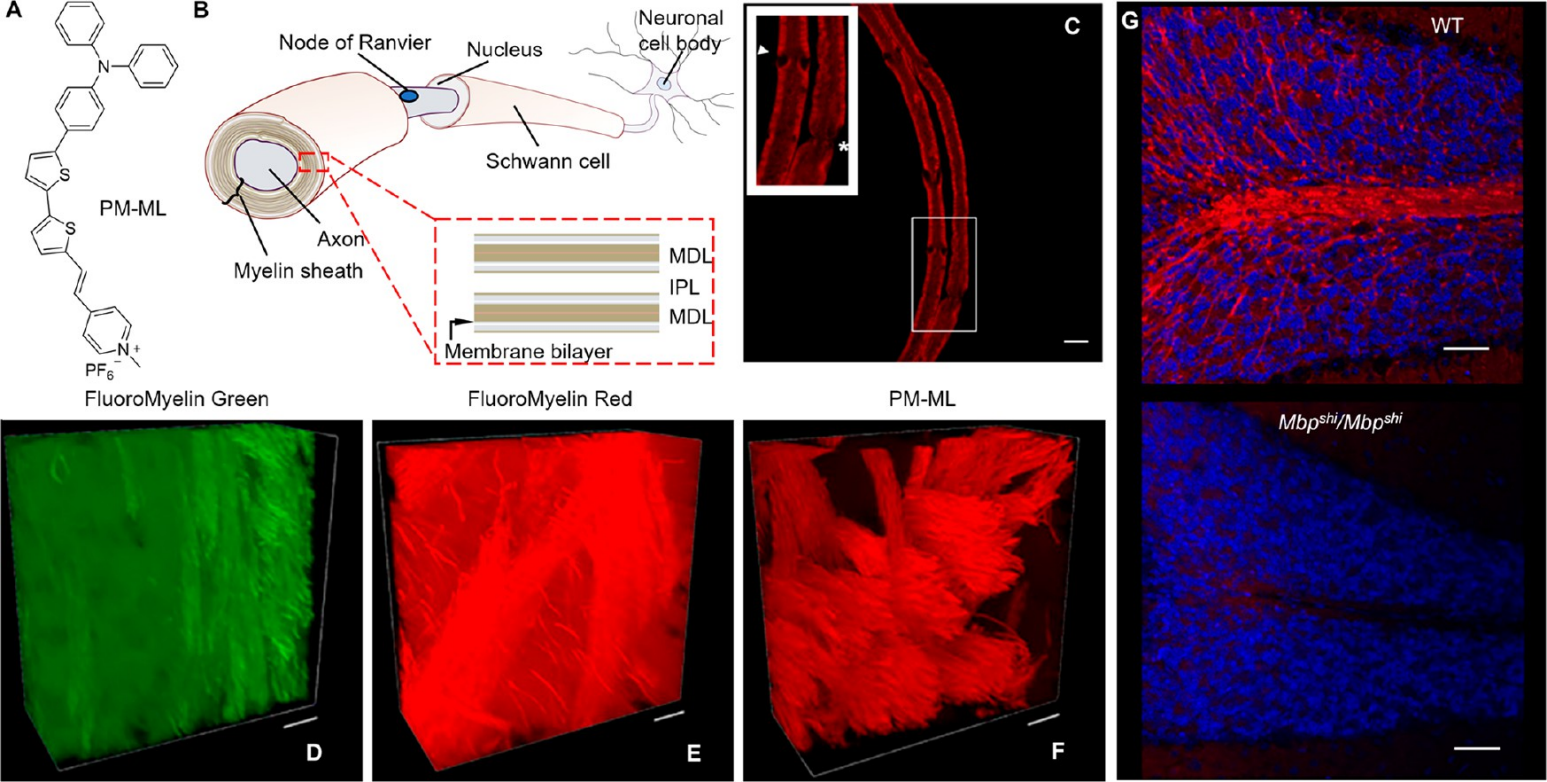
(A) Chemical structure of PM-ML. (B) A schematic illustration of an axon wrapped by a myelin sheath. (C) A segment of teased sciatic nerve fibers stained with PM-ML. The inset shows an enlarged region with Schmidt-Lanterman incisures (arrowhead) and a node of Ranvier (asterisk). Scale bar: 5 μm. (D–F) Three-dimensional rendering of Z-stacks of caudal putamen in the optically cleared mouse brain stained with (D) FluoroMyelin Green, (E) FluoroMyelin Red, and (F) PM-ML. Scale bars: 20 μm. (G) Overlaid images of sagittal sections in the cerebellum of wild-type (WT) or *shiverer* homozygous (*Mbp*^*shi*^/*Mbp*^*sh*^^i^) mice stained with PM-ML (red) and Hoechst 33342 (blue). Scale bars: 50 μm. Adapted with permission from ref (193). Copyright 2021 The National Academy of Sciences.

Chen’s group explored the potential application of an AIE probe in neuroimaging.^193^ They reported an AIE-active probe, named PM-ML, that showed significant fluorescence enhancement in the aggregate state or upon interacting with phospholipid bilayers (Figure 7A). Myelin is a biological structure composed of several layers of densely packed membranes that are wrapped around the axons (Figure 7B). It is particularly important for rapid nerve impulse propagation in axons of the central nervous system. PM-ML was rationally designed with a D-π-A structure and a nonplanar triphenylamine terminal unit as the basis for its AIE properties. PM-ML not only selectively binds to the myelin sheath in teased sciatic nerve fibers from the peripheral nervous system but also stained the myelinated fibers in mouse brain tissues (Figure 7C). PM-ML with a high signal-to-background ratio is superior to the commercial myelin stains Fluoromyelin Green and FluoroMyelin Red (Figure 7D–7F), which allowed neuroscientists to trace myelin fibers with PM-ML and to evaluate myelination in thick tissue slices. The application of PM-ML staining in assessing myelination for neuropathological studies was also demonstrated by using a mouse model with demyelination disease (Figure 7G).

Multiphoton fluorescence imaging has become another popular method for deep tissue visualization due to its advantages of longer-wavelength excitation and higher 3D resolution.^194−197^ Tang et al. reported a bright two-photon AIEgen (TTS) with a D-A structure.^198^ Resulting from a high two-photon absorption cross section at 900 nm under laser irradiation, a much deeper penetration (>200 μm) and mild fluorescence loss were achieved by TTS dots in excised liver tissue, clearly exhibiting advantages over one photon fluorescence. In addition two-photon AIE molecules with D-A structures have been developed by researchers for tumor tissues,^199,200^ fatty liver,^201^ and neurodegenerative diseases^202,203^ with high resolution and deep tissue penetration, showing the broad application of this strategy. Besides AIE molecular probes, AIE NPs were synthesized by a simple nanoprecipitation method for *ex vivo* two-photon tissue imaging. Niu et al. developed a synthetic strategy to produce AIE-active acrylonitriles (2TPAT-AN and TPAT-AN-XF) by simply tuning the reaction temperature.^204^ Given the large two-photon absorption cross-section at 880 nm of up to 508 GM due to their D-A structure and extended π-conjugation, as well as strong two-photon fluorescence, the TPAT-AN NPs prepared by nanoprecipitation methods showed a high signal-to-noise ratio and high contrast, which was distinctly revealed by the reconstructed 3D images. Such AIE NPs show great potential in two-photon deep tissue bioimaging and the long-term dynamic tracking of tumor metastasis.

For the application of AIEgens in tissue imaging, the particle size is one of the key factors in their distribution within the body. Normally, large AIE nanodots have intense accumulation in organs, such as the liver and kidneys, after intravenous injection, which limits their usage for imaging specific tissues. To enhance tumor targeting and reduce the retention in the liver, Jiang et al. used a microfluidic chip to control the assembly of AIEgens and their particle sizes.^205^ Instead of the conventional stirring or vortexing methods, microfluidics provided ultrahigh mixing efficiency and precise regulation of different components.^206,207^ In the presence of the coating agent DSPE-PEG, sub-10 nm AIE QDs were synthesized in the rationally designed double spiral channel. Four kinds of AIEgens were tested as QDs and nanodots. The small-size AIE QDs showed increased cell uptake as compared to the large-size ones. Small-size QDs could evade the liver and target the tumor more efficiently than the larger-sized ones.

### 2.3 *In Vivo* Imaging

*In vivo* imaging techniques allow scientists to study and understand the dynamic and complex biological processes such as tumor growth, gene expression, and drug delivery in living animal models, with real-time, in situ and noninvasive characteristics.^208,209^ In addition, the applied experimental objects are closer to the actual condition, which can better simulate the physiological and pathological processes of the organism. Significant advancements have been made in *in vivo* imaging to overcome its current limitations by NIR, multiphoton and PAI equipment, which have expanded its applications and increased its potential for clinical translation.^210−214^ Considering the successful demonstration of several rising examples for *in vitro* and *ex vivo* visualization, AIE probes are anticipated to offer good imaging performance in live animals.

#### 2.3.1 Near-Infrared Imaging

With enhanced light penetration and lower background signals, near-infrared (NIR) imaging can offer improved performance when compared with traditional techniques at the shorter wavelength region.^215−223^ Taking advantage of the AIE features and their long emission wavelength, NIR AIEgens can not only provide desirable photostability and enhanced fluorescence efficiency in aggregates but also exhibit reduced tissue scattering, enhanced penetration depth, diminished autofluorescence, and minimal photodamage to biological tissues.^224−228^ A win-win cooperation could be achieved by combining NIR emission into luminogens with AIE characteristics, enabling better bioimaging quality and rapid development for biological applications.

**Figure 8:**
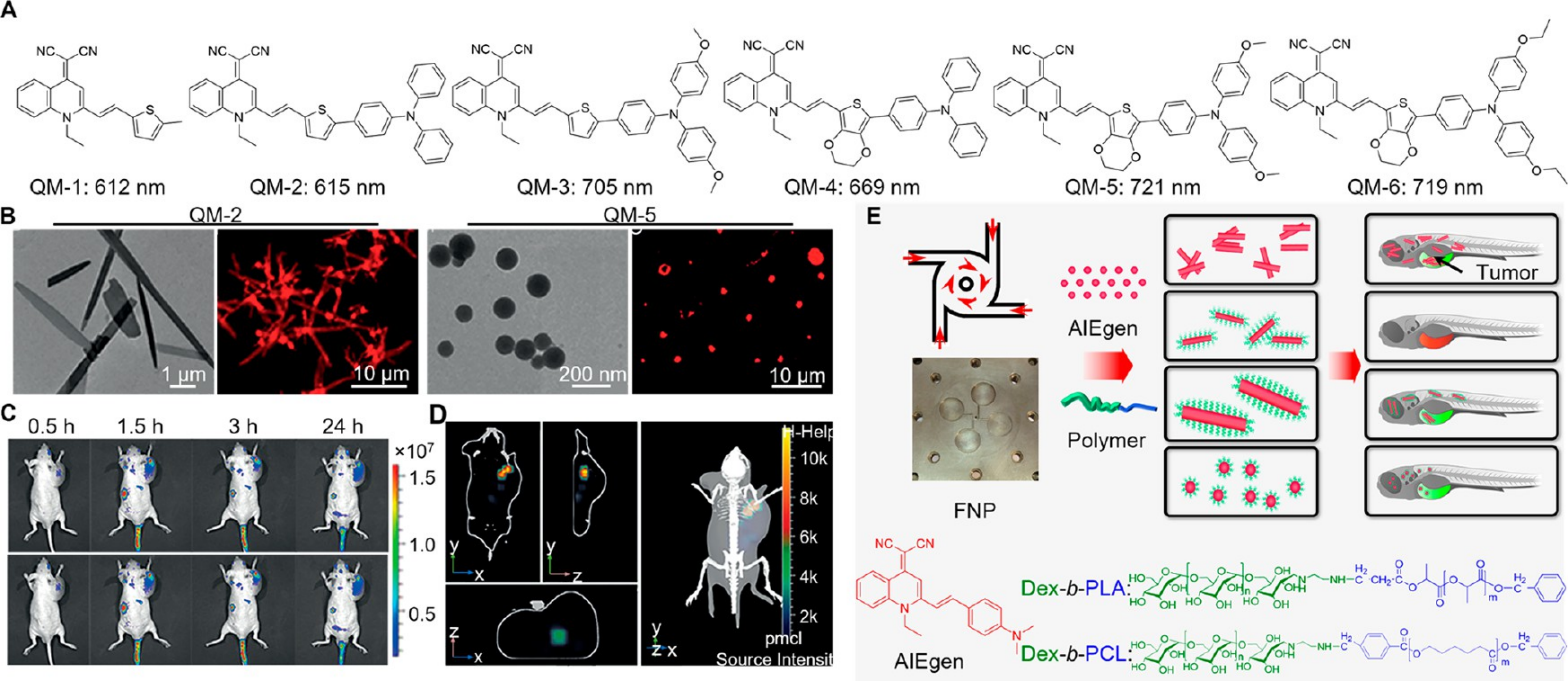
(A) Chemical structures and emission peaks of QM-based AIEgens in their aggregate states. (B) Transmission electron microscopy and confocal laser scanning microscopy images of the QM-2 and QM-5. (C) NIR fluorescence imaging of tumor-bearing mice after intravenous injection of QM-2 (top) and QM-5 (bottom). (D) The 3D fluorescence imaging of tumor-bearing mice after intravenous injection of QM-5. Adapted with permission from ref (229). Copyright 2015 Wiley-VCH. (E) Illustration of the morphology and size tuning by FNP and their effects on tumor cell imaging of zebrafish. Adapted with permission from ref (230). Copyright 2018 American Chemical Society.

Aiming to extend the emission wavelength of the AIEgens, the predominant molecular engineering strategy is focused on introducing large π-conjugated structures, strong electron-donating, and/or electron-accepting groups.^229^ When covalently linking quinoline-malononitrile (QM) with thiophene via a carbon-carbon double bond, the resulting QM-1’s emission wavelength peaked at only 612 nm (Figure 8A). After attaching a strong electron-donating alkoxytriphenylamine group to QM-1, the resulting QM-3 showed a longer emission at 705 nm. Furthermore, using the 3,4-ethylene-dioxythiophene (EDOT) unit produced QM-6, extending the emission to around 719 nm. Such structural modifications can not only tailor fluorescence wavelength, but also determine the morphology of the aggregates, as shown in Figure 8B. Further *in vivo* imaging demonstrated that rod-like QM-2 nanoaggregates displayed whole-body distribution, whereas spherical QM-5 nanoaggregates showed excellent tumor-targeting ability (Figure 8C and 8D). Furthermore, Zhu et al. introduced the flash nanoprecipitation (FNP) method to control the aggregation of AIE NPs.^230−232^ As shown in Figure 8E, AIEgens and amphiphilic block copolymers were dissolved in a water-miscible organic solvent, and then the organic solvent was mixed with water in the mixing chamber. Subsequently, NPs were obtained with AIE molecules encapsulated. Through engineering parameters, including the flow rate and solvent ratio, this FNP method can regulate the size, shape, and orientation of AIE NPs. Thus, the FNP method is a promising platform to achieve the facile and scale-up manufacture of AIE NPs, especially with a narrow size distribution, tunable physical and chemical characteristics, and good batch-to-batch reproducibility.

**Figure 9:**
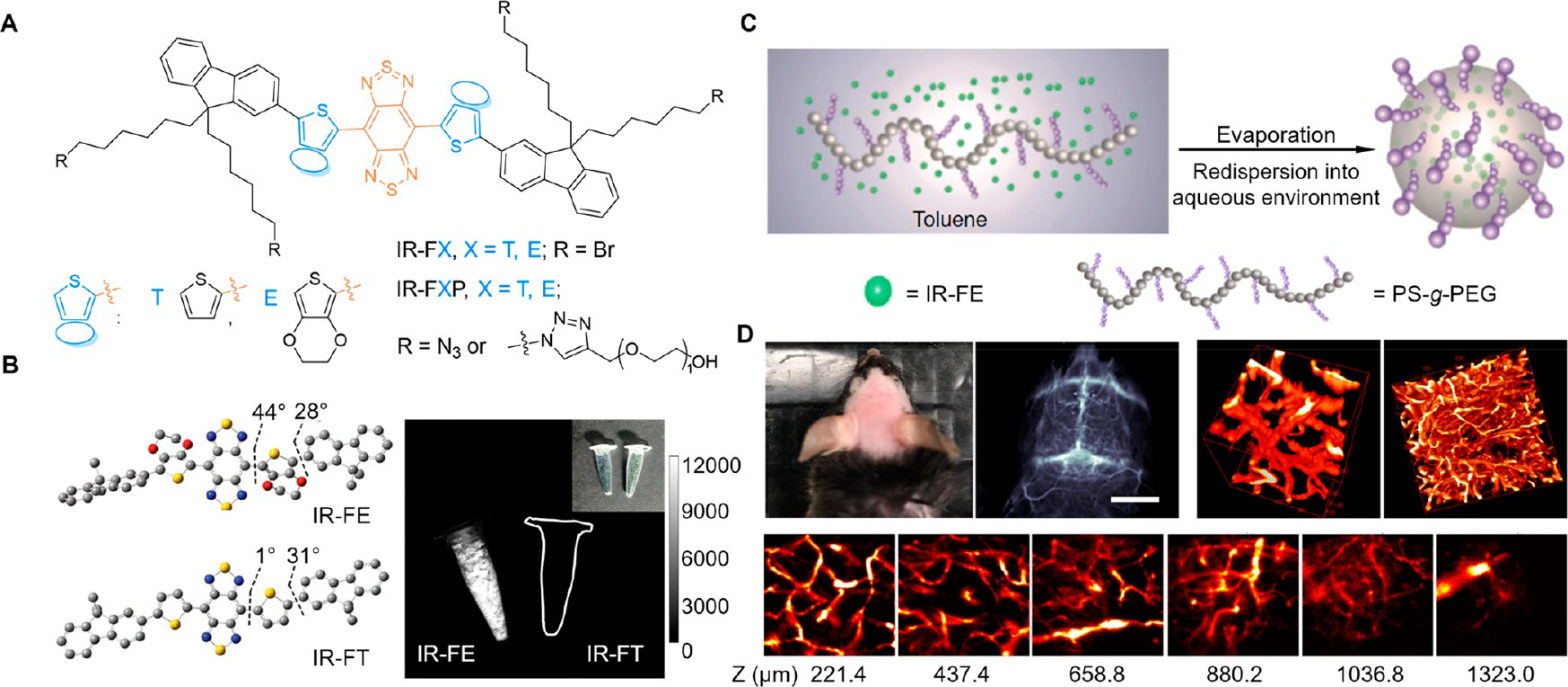
(A) Chemical Structures of the NIR-II fluorophores IR-FX and IR-FXP. (B) Optimized ground-state geometries of IR-FE and IR-FT and their powder fluorescence photograph. Adapted with permission from ref (233). Copyright 2017 Wiley-VCH. (C) Illustration of the encapsulation method for p-FE. (D) 3D imaging of brain vasculature of a mouse injected with p-FE. Exposure time: 5 ms. Scale bar: 6 mm. Adapted with permission under a Creative Commons CC BY License from ref (237). Copyright 2018 Springer Nature.

Enhancing the emission intensity is important for further promotion of NIR imaging and detection in biological research and clinical applications. To afford higher PLQY in aqueous solutions, Liang et al. utilized EDOT as the donor unit to construct NIR-II fluorophores IR-FEP (Figure 9A).^233^ Such a rather bulky EDOT unit significantly increased the dihedral angle between the donor and the benzobisthiadiazole (BBTD) acceptor, when compared to IR-FT with thiophene as the donor (Figure 9B). Interestingly, the PLQY of PEGylated IR-FEP reached 2% in water, which was around 100-fold higher than that of IR-FTP. The bulkier donor can efficiently shield the BBTD acceptor, reducing the interaction with water molecules and thus improving the aqueous QY.^233,234^ Benefiting from the AIE effect, IR-FE powder showed stronger NIR-II emission than the ACQ molecule IR-FT. A series of NIR-II fluorophores with backbone distortion between donor and acceptor have also been developed, and they showed enhanced PLQYs in aqueous solutions, partially correlated with the AIE effect originating from backbone distortion.^235,236^ Poly(styrene-*co*-chloromethyl-styrene)-*graft*-poly(ethylene glycol) (PS-*g*-PEG) was used as an amphiphilic polymer to encapsulate IR-FE and afford a p-FE (Figure 9C).^237^ Under the excitation of a laser at 808 nm, p-FE showed a strong emission peaking at around 1010 nm with a high PLQY of 16.5% in water. So far, several bright NIR-II AIEgens have been developed, and they exhibit promising application prospects.^238−240^ Owing to the high PLQY of p-FE, the exposure time could be reduced to 2 μs in the fluorescence brain imaging beyond 1100 nm, enabling real-time fast NIR-II imaging. One-photon 3D confocal imaging was also conducted on account of the high QY of p-FE. Clear distinguishability of the brain vessel depth from 221 to 1323 μm was demonstrated, and small vessels with a diameter of 5–7 μm could be clearly resolved (Figure 9D).

**Figure 10:**
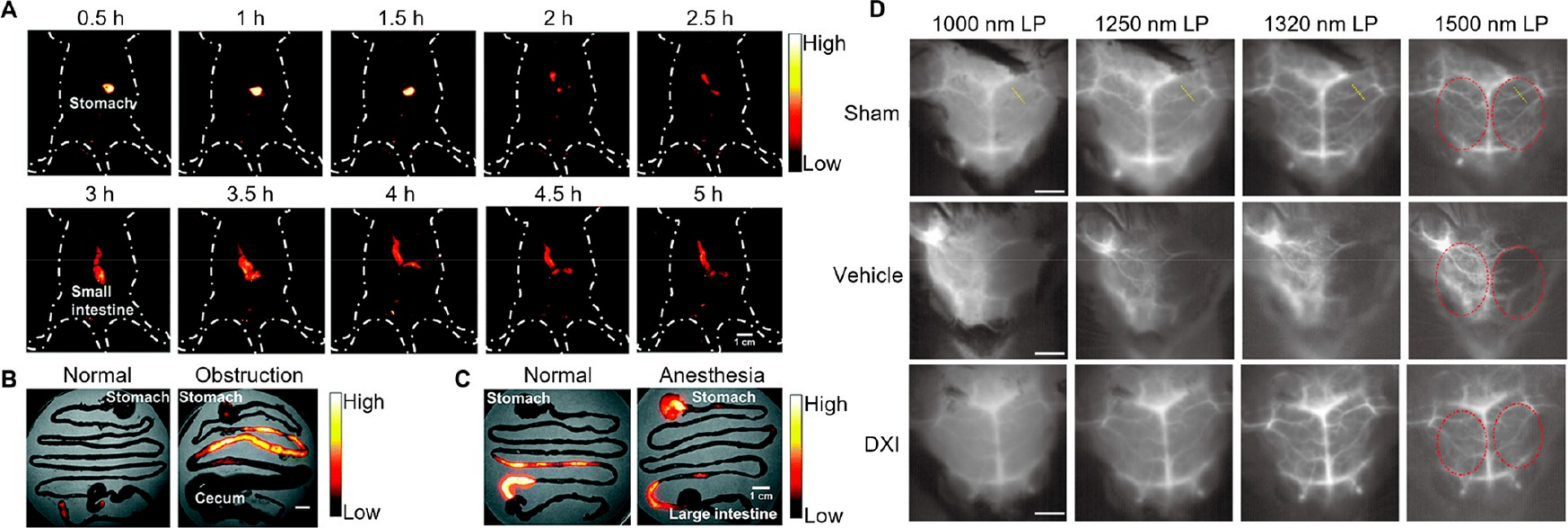
(A) Fluorescence images of the gastrointestinal tract in BALB/c mice gavaged with HLZ-BTED dots for visualizing intestinal obstruction. (B,C) NIR-II images of (B) the GI tract in normal mice or mice with intestinal obstruction at 5 h after gavage and (C) the GI tract in BALB/c mice that are normal or anesthetized by pentobarbital sodium at 2 h after gavage. Adapted with permission from ref (250). Copyright 2019 The Royal Society of Chemistry. (D) *In vivo* NIR-IIb imaging for ischemic stroke. Adapted with permission from ref (253). Copyright 2022 CCS Chemistry.

The *in vivo* optical imaging beyond 900 nm has been discovered to possess deeper penetration and higher resolution, compared with imaging in the 360–900 nm range.^241−244^ The NIR-II (1000–1700 nm) window along with its subwindows including NIR-IIa (1300–1400 nm) and NIR-IIb (1500–1700 nm) window, endow optical molecular imaging with desired penetration depth and spatial-temporal resolution, which has attracted much attention.^245−249^ Incorporating TPE or bulky modules into the backbone, NIR-II fluorophores can inherit AIE characteristics that not only minimize the quenching effect but also promote the overall fluorescence intensity and the emission tails wavelength moves into the NIR-IIa and NIR-IIb regions. The NIR-II AIE fluorophore, HLZ-BTED based on benzobisthiadiazole (BBTD) and TPE reported by Hong’s group was successfully used for blood vessels, long-term breast tumor, ischemia, the gastrointestinal (GI) tract, and intestinal obstruction NIR-II imaging (Figure 10A–10C).^250^ Very recently, a few highly twisted small-molecule NIR-IIb AIE imaging agents, such as HL3, HQL2, and HY4, have been used for NIR-IIb vascular imaging.^251−253^ HL3 and HQL2 dots showed a increase in PLQY of ∼11.7% and ∼11.9% over 1000 nm and ∼0.05% and ∼0.02% over >1500 nm in aqueous solution, respectively. Notably, by utilizing the tail emission of HL3 and HQL2 at 1500 nm, NIR-IIb imaging of blood vessels, cerebral vasculature, lymphatic drainage, and U87MG-tumor angiogenesis was successfully achieved with near-zero background noise, high resolution, and a high signal-to-noise ratio. Using the NIR-IIb emissive AIE HY4 dots, Hong and co-workers achieved real-time feedback on the therapeutic efficacy of Chinese medicine Dengzhan Xixin injection on ischemic stroke, as shown in Figure 10D.^253^ Aiming to prolong the imaging window beyond 1500 nm, AIE dots with highly twisted structures were used to precisely image the mice brains in the NIR-IIb window.^254^

**Figure 11:**
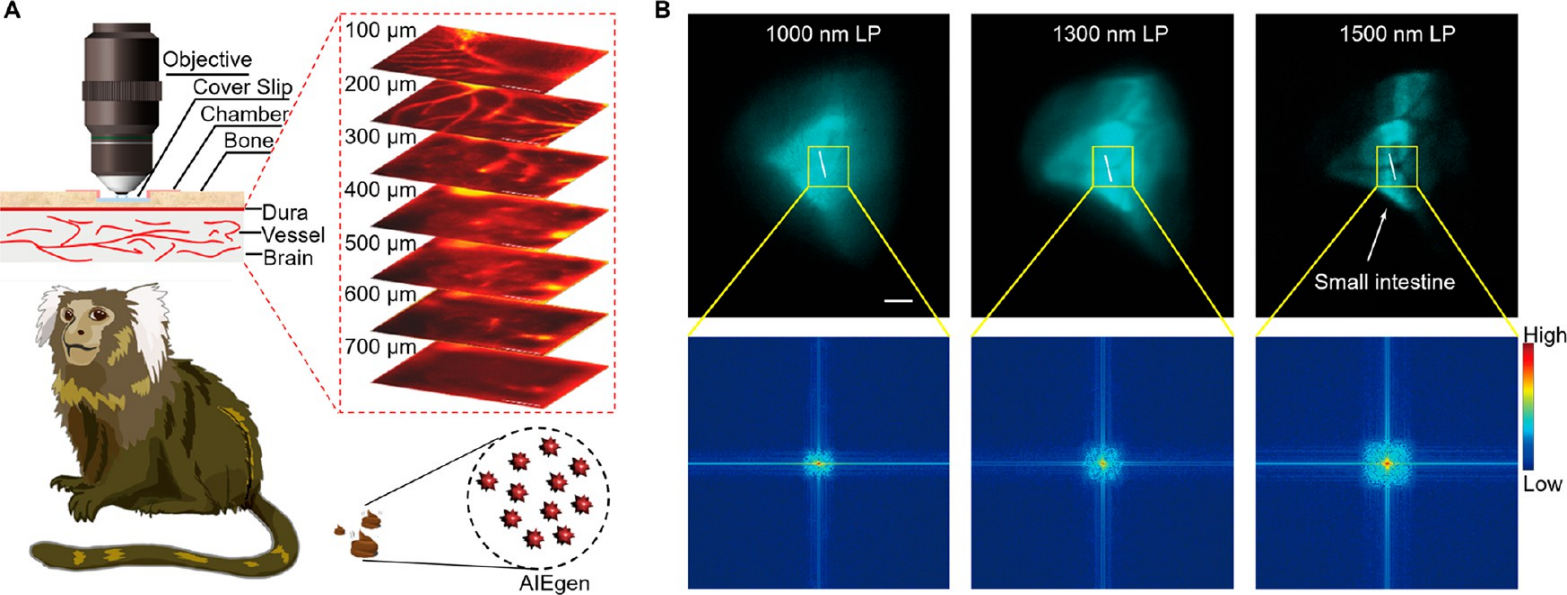
(A) Through-thinned skull cerebrovascular microimaging. (B) NIR-IIb fluorescence GI imaging. Scale bar: 5 mm. Adapted with permission from ref (255). Copyright 2021 Wiley-VCH.

Further, as an essential step toward deciphering life science, imaging targets have been expanded to nonhuman primates from mice/rats/rabbits. Herein, excretable and bright NIR-II AIE dots with emission tailing beyond 1500 nm have been developed. The NIR-II fluorescence microscopic monitoring through-thinned-skulls of nonhuman primates brain vessels using microscopic monitoring and the NIR-IIb fluorescence GI imaging was successfully achieved (Figure 11A and 11B).^255^ Centimeter-deep and three-photon fluorescence NIR-II vascular imaging in nonhuman primates has also been realized.^256,257^ Further, a series of quantitative approaches have been developed to assess the structural changes of deep vessels using biocompatible AIE dots.^258^ The morphological changes and organization remodeling of cortical blood vessels in marmosets during stroke have been fully and accurately analyzed.^259,260^

**Figure 12:**
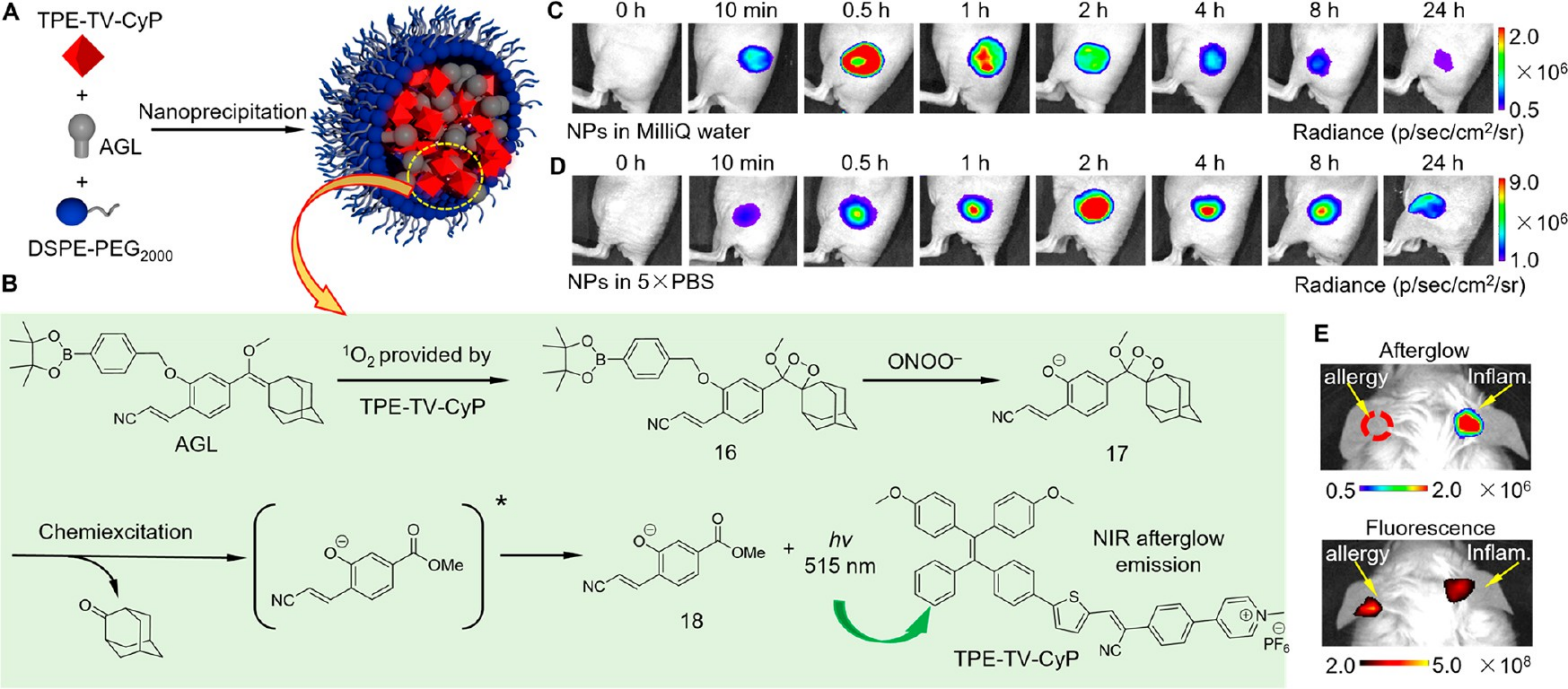
(A) Schematic of preparation of an ONOO^–^-activated NIR afterglow nanoprobe. (B) A series of reactions occurred within the NP for NIR afterglow luminescence. (C,D) Representative afterglow images of the inflammatory sites after injection (*in situ*) with preirradiated afterglow nanoprobes dissolved in (C) Milli-Q water or (D) 5× PBS buffer at various time points. (E) Representative images of afterglow and fluorescence imaging of a mouse model bearing with left ear allergy while right ear inflammation. Adapted with permission from ref (268). Copyright 2022 American Chemical Society.

Afterglow luminescence is an optical phenomenon where some substances keep emitting the light after stopping their optical excitation.^261^ By means of this property, afterglow imaging exhibits many advantages when compared with traditional fluorescence imaging such as no background signal interference, ultrahigh sensitivity, no real-time excitation, etc.^262−266^ To date, although there exist several kinds of afterglow materials such as rare-earth metal agents, semiconducting polymers, and small organic molecules, real applications including bioimaging in complex living contexts is still limited due to poor response to targets and weak emission in aqueous environments.^267^ Recently, Ding et al. conjugated an ONOO^–^ responsive group to the enol ether precursor of Schaap’s 1,2-dioxetane. A similar nanoengineering strategy by using twisted AIEgen was employed to prepare an ONOO^–^ and pH dual-responsive NIR afterglow nanoprobe with enhanced afterglow luminescence for the study of neutrophil-involved disease and antitumor drug screening.^268^ The process for preparing the nanoprobe is shown in Figure 12A, and the series of reactions that occurred within the nanoprobe are depicted in Figure 12B. Briefly, before use, the precursor of Schaap’s 1,2-dioxetane with a phenylborate moiety (compound AGL) needs to be oxidized to form compound **16** by singlet oxygen provided by the AIEgen (TPE-TV-CyP) under white light irradiation. Then, once exposed to ONOO^–^ at physiological pH, the responsive group (phenylborate moiety) of compound **16** was removed to form compound **17**, which then underwent decomposition and triggered chemiexcitation along with green light emission. Finally, the efficient energy transfer in the NP microenvironment resulted in the AIEgen emitting NIR luminescence without excitation. It is noteworthy that the responsive behavior was closely related to pH. Apart from the high selectivity toward ONOO^–^ at pH 7.4, lower pH values induced weaker afterglow activation, indicating the ONOO^–^ and pH dual-responsive nature of this nanoprobe. Inspired by its dual-responsive nature, the nanoprobe was used to explore acute skin inflammation, distinguish allergy and inflammation, and screen immunogenic cell death drugs. As displayed in Figure 12C, before and after inducing acute inflammation by LPS injection, the inflamed skin tissues of the mice were administrated with the nanoprobe dissolved in Milli-Q water in eight groups according to the predetermined time points. The afterglow activated for 10 min, which suggested the rapid recruitment of neutrophils accompanied by O^–^ release. The dynamic profile of afterglow intensities at different time points revealed the combined influence of both ONOO^–^ and pH changes on the progression of acute skin inflammation. To exclude the influence of pH on the response of the nanoprobe, the authors dissolved the nanoprobe in 5× PBS buffer and performed the same imaging experiments (Figure 12D). Interestingly, the maximum of the afterglow intensity appeared at 2 h instead of 0.5 h, suggesting that the ONOO^–^ level reached a peak at 2 h. Therefore, using this nanoprobe, real-time monitoring of the progress of neutrophil infiltration along with ONOO^–^ generation was realized. Furthermore, according to the large difference in neutrophil infiltration, the authors achieved a rapid *in situ* discrimination of allergy and inflammation using a mouse model for the two diseases (Figure 12E), saving much time and effort compared to conventional tissue analysis.

#### 2.3.2 Image-Guided Surgery

**Figure 13:**
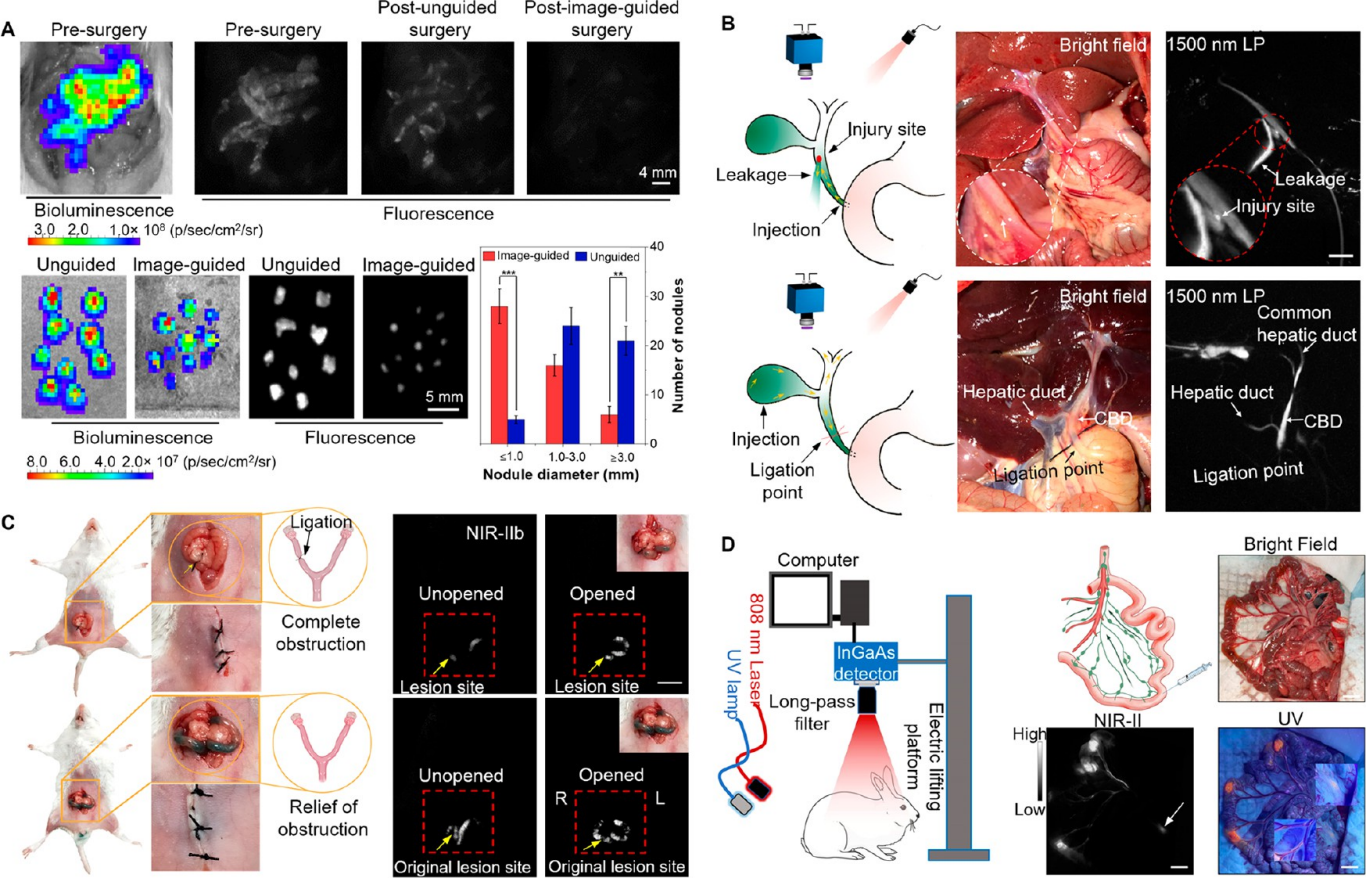
(A) NIR-IIa image-guided tumor resection in mouse models. Adapted with permission from ref (270). Copyright 2020 American Chemical Society. (B) NIR-IIb imaging of biliary injuries in rabbit models. Adapted with permission from ref (271). Copyright 2021 American Chemical Society. (C) NIR-IIb detection of the complete uterine obstruction in mouse models. Scale bar: 10 mm. Adapted with permission from ref (272). Copyright 2021 Elsevier. (D) NIR-II and visible fluorescence hybrid image-guided surgery of the mesenteric lymph nodes in rabbit models. Scale bars: 1 cm. Adapted with permission from ref (273). Copyright 2022 Elsevier.

The NIR-II imaging provides a suppressed imaging background, conducive to the precise surgery in the clinical settings.^269^ A pH-responsive AIE polymer, which could specifically accumulate at tumor sites, achieved NIR-IIa fluorescence image-guided cancer resection (Figure 13A).^270^Figure 13B illustrates intraoperative observation of acute biliary injuries in rabbit models using NIR-IIb fluorescence of the AIE dots.^271^ Through surrounding subcutaneous adipose tissues, perimetric fat pad, muscle layer, etc., Zhang and co-workers performed NIR-IIb fluorescence hysterography and recorded the repair of the uterine rupture and the relief of the uterine obstruction with nonradioactive risks (Figure 13C).^272^ Recently, Lin and co-workers developed a synergistic optical navigation mode using both visible and NIR-II fluorescence of the hybrid AIE probes, which shortened the duration of image-guided surgery and could potentially provide a potentially translational strategy (Figure 13D).^273^ Besides, utilizing the NIR-II fluorescence for the precise diagnosis and effective treatments for inflammatory bowel diseases, a complete resection of severe inflammatory bowls and a secure surgical anastomosis was achieved.^274^ As an alternative to fluorescence image-guided surgery, afterglow imaging was has also been used in cancer surgery.^275^

### 2.4 Pathogen Imaging

Microorganisms that exist everywhere are intimately associated with human life in all aspects. However, a large A large percentage of microorganisms are pathogens that can infect animals and plants and cause varying diseases.^276^ Increasing efforts have been devoted to acute and real-time imaging of pathogenic bacteria.^277−279^ Since bacterial cytoplasmic membranes are negatively charged, positively charged species can target bacteria specifically owing to electrostatic interactions.^280,281^ Due to the turn-on fluorescence feature, AIEgens can be employed as promising tools for differentiating dead and living bacteria, long-term tracking of bacterial viability and evaluating bacterial susceptibility to antibiotics.^282−284^ Bacteria can be divided into Gram-positive (G^+^) and Gram-negative (G^–^) subtypes, which is associated with differences in the cell wall structure and components.^285^ Considering the different roles and threats of varying types of bacteria, it is desirable to rapidly discriminate bacteria and further provide valuable information assisting the choice of appropriate antibacterial agents for accurate and efficient bacterial eradication.^286−289^ A series of AIEgens with orderly enhanced D-A strength were developed for the discriminative imaging of Gram^+^ bacteria.^290^ As an alternative to electrostatic interactions, hydrogen-bonding interactions were used in targeting bacteria.^291^

Aside from pathogenic bacteria, fungi and viruses can also cause serious illnesses.^292^ Fungi can proliferate rapidly under favorable environmental conditions, and they can even survive in extreme conditions.^293^ While many types of fungi are harmless, some species can cause serious infections in humans, particularly in people with weakened immune systems. Various AIEgens have been developed for fungal imaging and detections.^294,295^ Tang’s group has reported a method for visually discriminating between G^+^ bacteria, G^–^ bacteria, and fungi.^296^ They used an AIE-active probe with TICT properties that could locate in different sites of the three pathogens, displaying three discernible emission colors. G^–^ bacteria appeared as weak pink, G^+^ bacteria appeared as orange-red, and fungi appeared as bright yellow. Further, a water-soluble AIEgen, named DPASI, was represented as a promising approach for accurate and efficient fungal viability detection.^297^ DPASI could diffuse into dead fungi with an impaired cell wall and membrane, where it bound with mitochondria, giving a yellow emission. In contrast, DPASI cannot enter live fungi due to their intact cell walls and membranes, making it a reliable tool for distinguishing between live and dead fungi. The imaging and detection on viruses are discussed in Section 4.2.

To provide a clear overview of the AIEgens in Section 2, we have summarized their compound names and structures, applications and advantages, imaging modes, and references in Table 1.

**Table 1:**
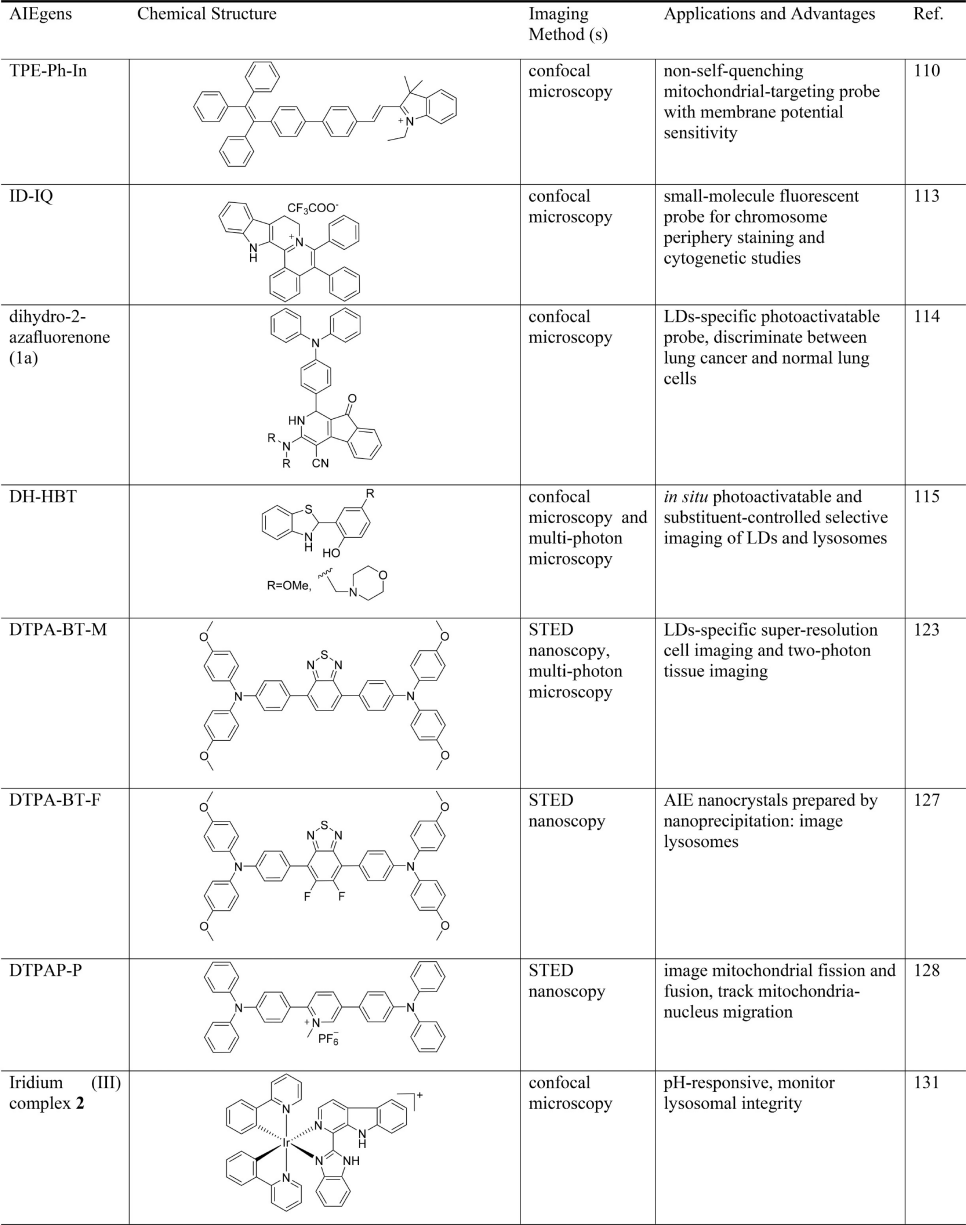

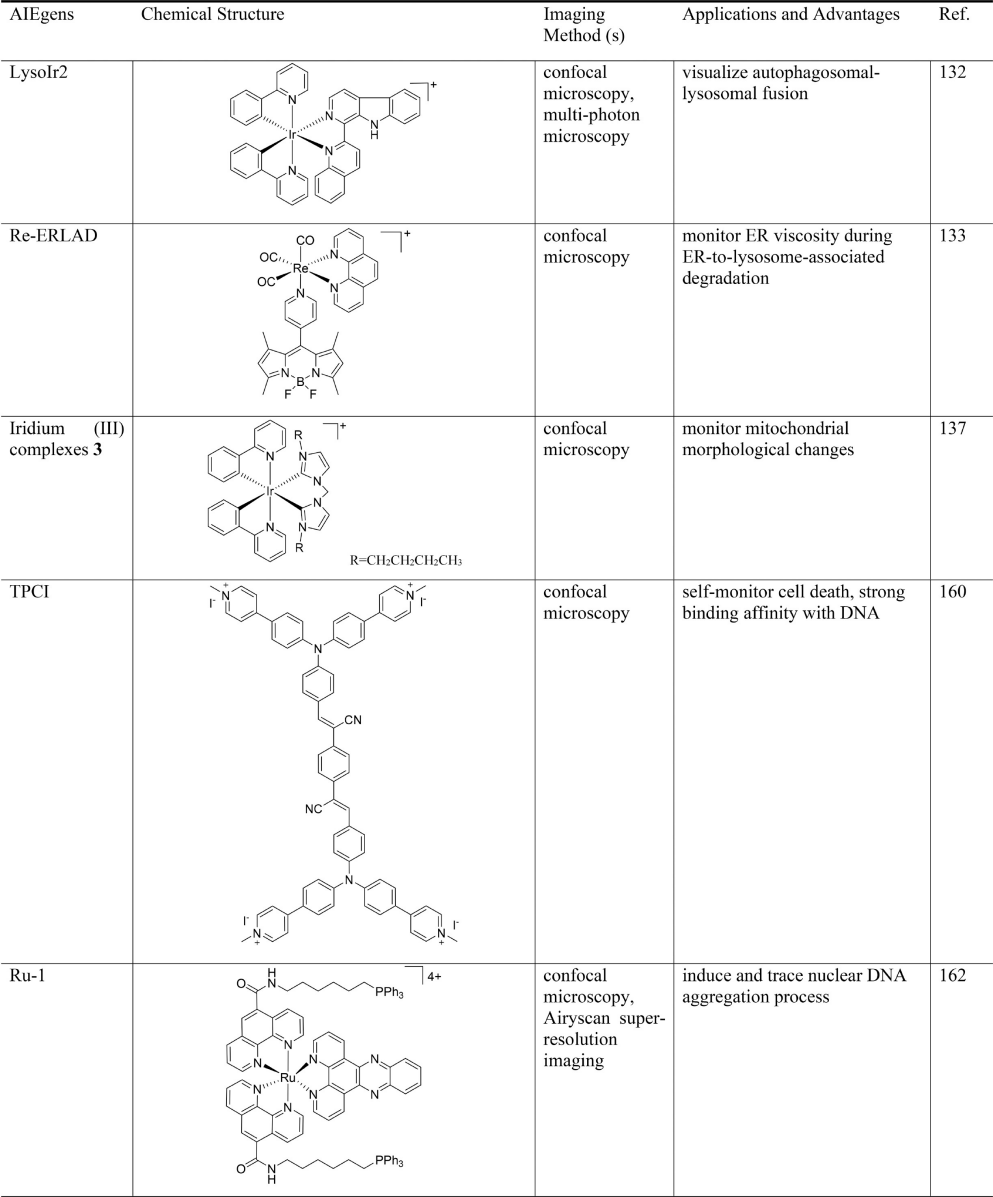

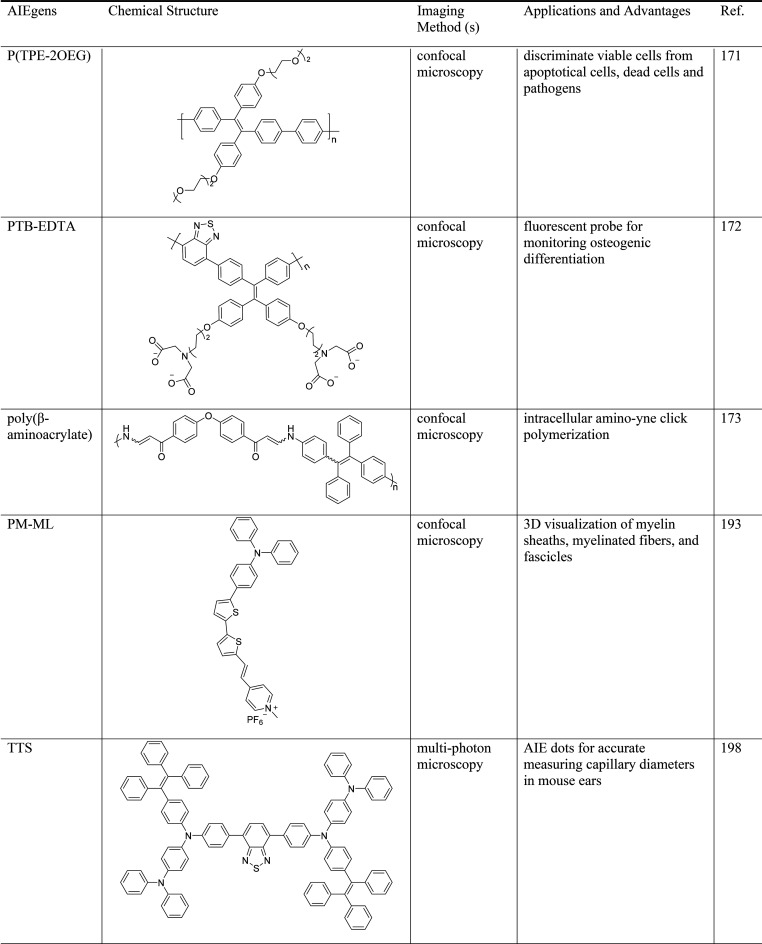

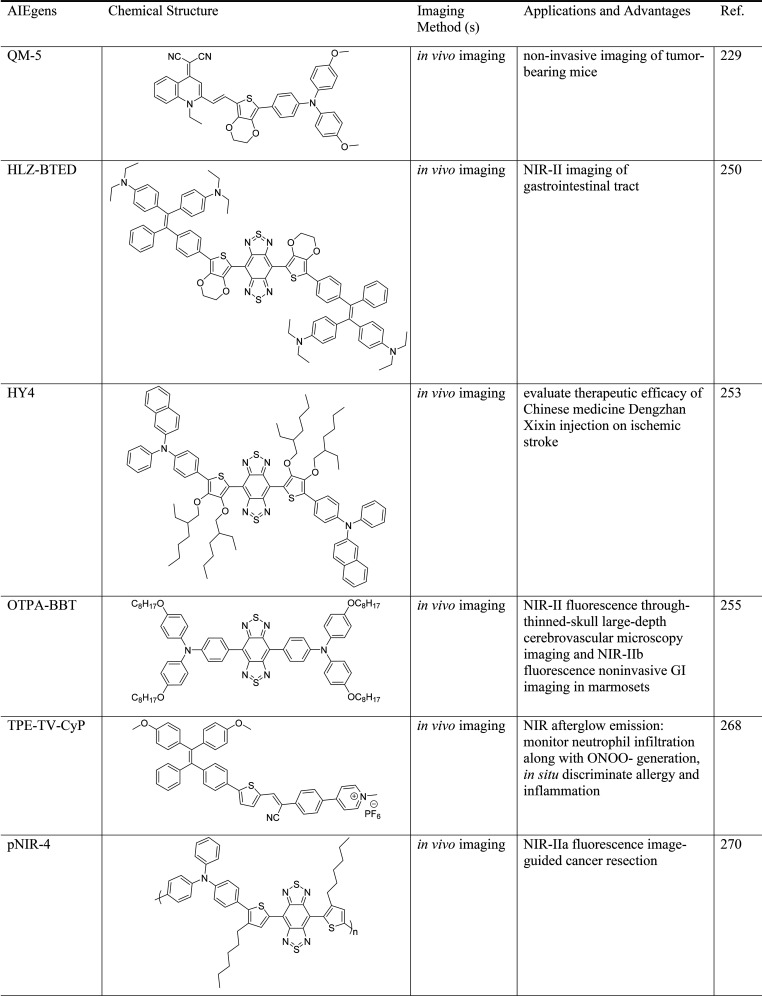

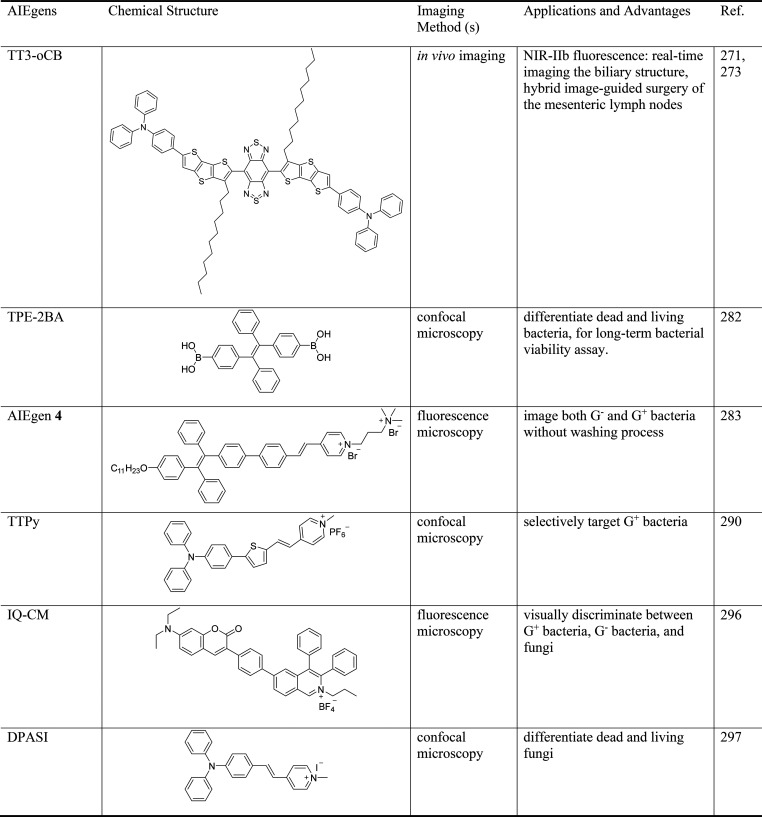
Reported AIEgens for Biological Imaging

## 3 Combat Diseases: Cancer Theranostics and Bacterial Elimination

Health has always been of critical global concern, as human beings have been battling various diseases since the inception of human civilization. With the development of modern society, diseases have increasingly raised medical and public concerns since they affect most people worldwide. Effective diagnosis and treatment of diseases are essential for protecting and improving public health and societal well-being.

As a global disease, cancer represents an ever present and increasing threat to the health of human beings because of an increasing morbidity rate, relapse rate, and low survival rate.^298^ There was an estimated 19.3 million new confirmed cancer cases and approximately 10 million deaths globally in 2020.^299^ The rate of cancer-related diseases is rising every year, with an estimated annual death of more than 13 million people by 2030.^300^ Thus, the diagnosis and treatment of cancer is of critical concern and has received enormous attention in life science and clinical research.^301−307^ Currently, the main methods of clinical treatment for tumors include surgery, chemotherapy, and radiotherapy.^308^ However, these treatments can cause side effects, such as systemic toxicity, unavoidable invasiveness, and a high risk of relapse. Therefore, the development of effective technologies for cancer diagnosis and therapy remains an urgent and challenging task.

Bacterial infections, another type of prevalent diseases, have seriously threatened human health and affected millions of people worldwide.^309−311^ Since the discovery and popularization of penicillin in 1928, the widespread use of antibiotics has greatly reduced the high mortality caused by bacterial infection and temporarily ended the end of infectious diseases.^312^ Unfortunately, the increasing abuse and misuse of antibiotics has led to the development of antibiotic resistance in some bacteria, forcing us back into the battlefield by restarting the fight against bacterial infections.^313^ Consequently, alternative therapeutic strategies with high specificity and efficient antibacterial activity are urgently needed.^314,315^

In comparison with traditional fluorescent materials with the ACQ effect, luminescent molecules with the AIE effect have been successfully demonstrated to exhibit significant potential in cancer therapy and bacterial elimination.^316−326^ In this section, representative research involving powerful AIEgens are cataloged by their biofunctional effects, ranging from diagnosis to treatment. For cancer therapy, PDT and combined therapy are discussed in sequence. For bacterial elimination, the tracking and killing of extracellular and intracellular bacteria are introduced sequentially.

### 3.1 Cancer Theranostics

Despite significant breakthroughs in cancer therapy over the past few decades, effective diagnosis and subsequent therapeutic interventions remain challenging.^327−333^ Compared with traditional treatment methods, phototheranostics, emerging as a promising pattern of cancer therapy, integrates photoinduced diagnostic imaging and concurrent *in situ* therapy into a single formulation with the merits of high spatiotemporal precision and noninvasiveness.^334−339^ The working mechanism behind phototheranostics is that a fluorophore transfers the absorbed photon energy into different forms of energy for disease diagnosis and therapy.^77^ On the one hand, when the absorbed photon energy of molecules relaxes through a radiative pathway, the generated fluorescence signal could be used for diagnostic fluorescence imaging with the merits of high sensitivity, real-time tracking ability, high spatiotemporal resolution, and noninvasiveness.^76^ As we already considered above, compared to the shorter wavelength region, fluorescence imaging in the NIR region has attracted great attention in biomedical research owing to its deeper tissue penetration, lower tissue autofluorescence interference, and less photodamage.^340^ On the other hand, the absorbed photon energy of molecules can also be used for generating ROS and heat, leading to PDT and PTT modalities, respectively, toward cancer treatment with the advantages of precise manipulation, high selectivity, noninvasiveness, and negligible drug resistance.^341,342^ Besides, the generation of heat can simultaneously produce acoustic waves that can be applied to PAI for diagnosis with an even deeper tissue penetration depth and a higher signal-to-noise ratio than fluorescence imaging.^343,344^ Nevertheless, these photophysical energy dissipation processes compete with each other, since energy should be conserved. Therefore, to meet practical demands, the rational design of AIE phototheranostic systems and manipulating their absorbed photon energy dissipation for generating fluorescence for imaging, ROS for PDT and heat for PAI/PTT according to practical demand are important and will be discussed.

#### 3.1.1 PDT

PDT involves the use of PS, light, and molecular oxygen to kill cancer cells.^345−349^ When a PS is excited with light, electrons are pumped to higher-energy orbitals from the singlet ground state and then subsequently relax to its lowest excited singlet state (S_1_) according to Kasha’s rule.^65,350^ A suitable small energy gap between S_1_ and excited triplet state (T_1_) results in S_1_→T_1_ ISC.^351^ Afterward, the energy of the PS in long-lived T_1_ state will be transferred to adjacent molecular oxygen (^3^O_2_), producing highly reactive and toxic singlet oxygen (^1^O_2_) through a type II reaction.^352^ Besides, a PS in long-lived T_1_ state can also participate in the formation of superoxide (O_2_^• –^) or hydroperoxyl radicals (HO_2_^•^) through transferring electrons to cellular substrates through a type I reaction.^353−355^ Different kinds of ROS and radicals generated by those two types of photochemical reactions can kill cancer cells in a PDT therapeutic manner.^356−358^ Selective and efficient PDT can also be realized by precisely manipulating the light irradiation area, which is an effective way to minimize side effects to healthy tissues.^359^ Besides, PDT exhibits several other merits such as noninvasiveness, few side effects, negligible drug resistance and low systemic toxicity, all of which has motivated scientists to work on PDT as a therapeutic modality for disease treatment.^360,361^ As one of the key elements in PDT, various PSs such as porphyrin, chlorin, phthalocyanine, squaraine, BODIPY derivatives, and transition metal complexes have been explored so far to date.^362−366^ The development of PSs with a superior organelle targeting capability can help to realize precision PDT toward cancer treatment.^367,368^ However, most of the existing PSs are still far from the ideal, they should possess characteristics including strong absorption at long wavelengths, efficient photosensitization, negligible dark toxicity, and good photostability and biocompatibility.^67,363^ In addition, these conventional PSs often possess a rigid planar π-conjugated structure and are prone to aggregate in aqueous media, resulting in significantly decreased photosensitization behavior and emission, which is another critical obstacle for conventional PSs in PDT applications.^65,369^ In this regard, the discovery and development of PSs with AIE characteristics presents a promising alternative approach.^370−373^ Herein, the application of AIE PSs in organelle targeted PDT and PDT-induced treatment will be discussed.

##### 3.1.1.1 Organelle-Targeted PDT

**Figure 14:**
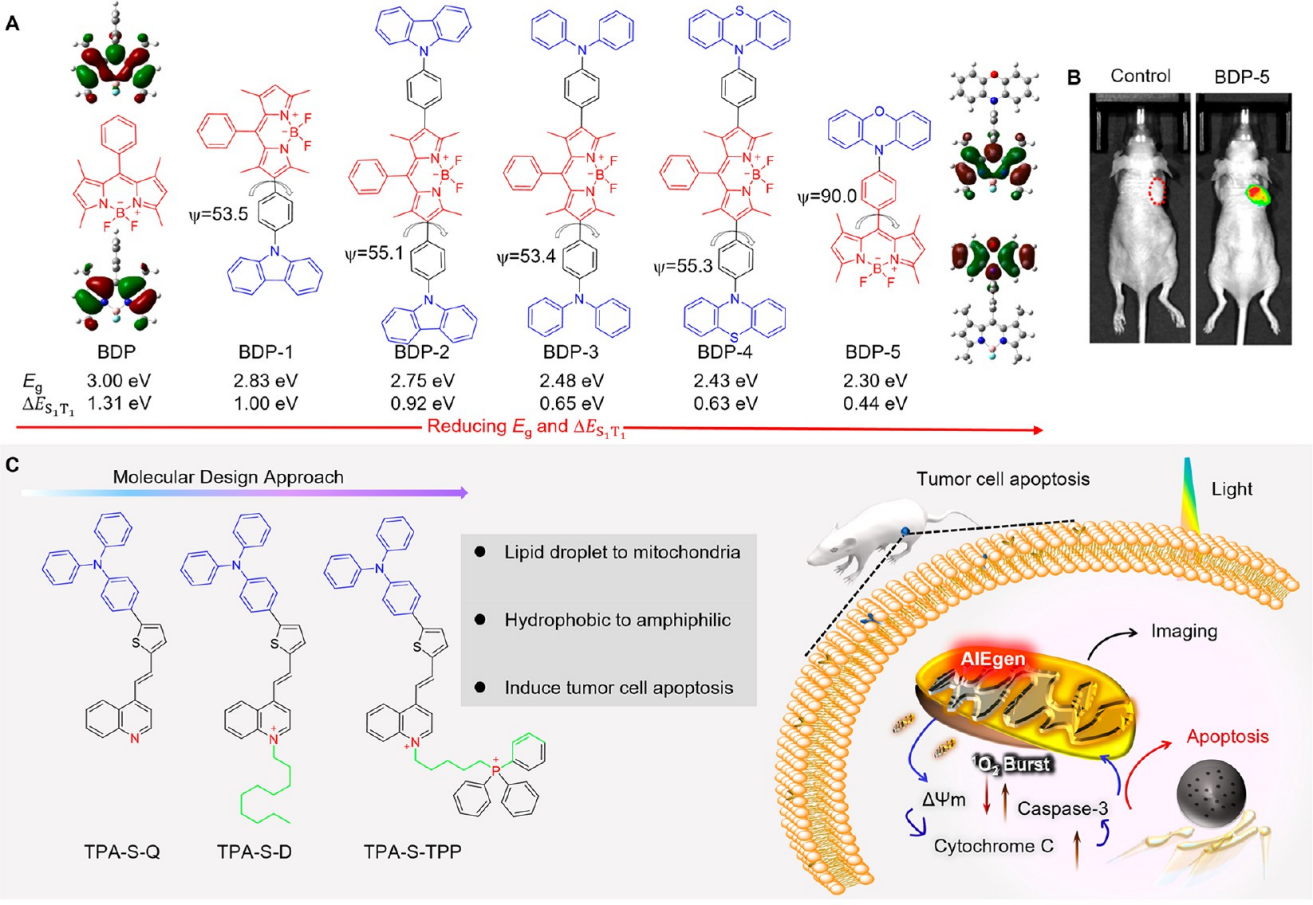
(A) Density functional theory (DFT) calculations of BDP dyes. (B) Tumor imaging and antitumor results treated with BDP-5 nanoparticles. Adapted with permission from ref (381). Copyright 2020 Wiley-VCH. (C) Molecular design approach of AIEgen TPA-S-TPP and its antitumor mechanism. Adapted with permission under a Creative Commons Attribution 3.0 Unported License from ref (382). Copyright 2021 The Royal Society of Chemistry.

As one of the most indispensable subcellular organelles, mitochondria are considered as a critical target for cancer therapy since mitochondria-targeted therapy can directly suppress the energy supply for cells and magnify the therapeutic effect.^374,375^ BODIPY dyes are widely used in bioimaging and tumor treatment by virtue of several advantages such as high molar absorption coefficients, high photostability, and ease of modification.^376−380^ In the field of PDT, many elegant PS have been designed to increase ISC through the introduction of heavy halogen atoms (notably bromine and iodine).^377^ Although they exhibit great potential for clinical transformation in the evaluation of antitumor effects, shortcomings include low fluorescence and dark toxicity, which hinders their further biological application. Therefore, the rational construction of heavy-atom-free BODIPY PSs is highly desirable. Inspired by the spin-orbit charge transfer ISC mechanism, Yoon’s group designed a series of BODIPY dyes with twisted D-A/D-A-D skeletons by tuning the section of electron donors, including carbazole, diphenylamine, phenothiazine, and phenoxazine (Figure 14A).^381^ Combined with DFT calculations, their study revealed that the electron donor (phenoxazine) and twisted molecular structure synergistically enabled BDP-5 with an Δ*E*_ST_ as low as 0.44 eV, which demonstrated the concept of reducing the Δ*E*_ST_ of BODIPY dyes for imaging-guided PDT. BDP-5 was AIE-active and showed a good ability to produce ROS under light irradiation. Utilizing Pluronic F127 as the encapsulation medium, BDP-5 NPs were obtained, able to effectively kill tumors in mice (Figure 14B). The regulation of molecular structures not only affects their aggregation and cellular localization, but also significantly affects the therapeutic effect on tumors. In 2021, Yoon and Peng proposed a molecular design approach to obtain a mitochondrial-targeted amphiphilic AIEgen TPA-S-TPP with NIR fluorescence of 737 nm, by balancing the lipophilic and hydrophilic moiety (Figure 14C).^382^ The smart AIEgen TPA-S-TPP displayed a “fluorescence-off” state before locating in the mitochondria of cancer cells and anchored firmly in the mitochondria with intense fluorescence. In contrast to commercial mitochondria dyes, AIEgen TPA-S-TPP overcame the long-term dependence on the mitochondrial membrane potential (ΔΨ_m_), effectively avoiding its spillover from the mitochondria. Upon white light irradiation, AIEgen TPA-S-TPP generated a high dose of ^1^O_2_ in the mitochondria of cancer cells, resulting in a decrease of the ΔΨ_m_ and release of cytochrome *c*, which activated the caspase-3 activity and induced apoptosis. Due to its good biocompatibility, tumor growth in mice were efficiently inhibited after treatment with AIEgen TPA-S-TPP. This successful example provides a direction for the construction of improved amphiphilic AIE theranostic agents for the treatment of tumors.

**Figure 15:**
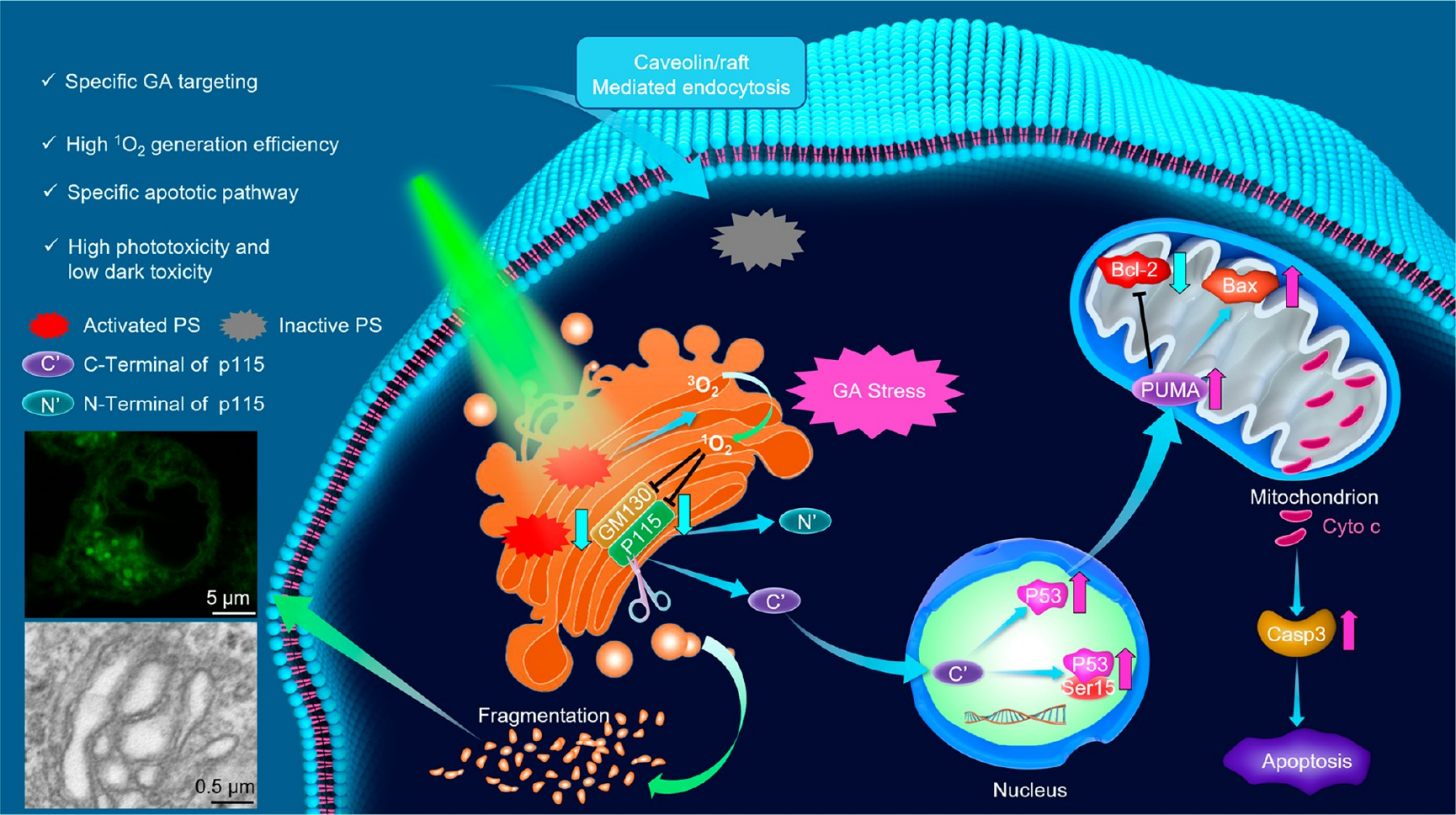
Schematic illustration of the GA-targeting mechanism and apoptotic pathway induced by ROS generation. Adapted with permission under a Creative Commons CC BY License from ref (383). Copyright 2022 Springer Nature.

The Golgi apparatus (GA) plays important roles in various physiological processes, and dysfunction of the GA is related to severe illnesses such as Alzheimer’s disease and Parkinson’s disease. AIEgen-based PSs with high ROS generation rates and efficient GA targeting abilities could cause severe damage to the GA and effectively kill tumor cells. Chen and co-workers reported a series of AIEgen-based PSs with an excellent GA targeting ability (Pearson correlation coefficient up to 0.98) and high ^1^O_2_ generation efficiency (Figure 15).^383^ Modification using a polar −CN group enhanced the intermolecular interactions and facilitated the formation of rod-like NPs with 200–400 nm size, which specifically accumulated in the GA via caveolin/raft mediated endocytosis. Meanwhile, the introduction of a pyrene group into the D-π-A scaffold helped separation of the electron cloud distribution of the highest occupied and lowest unoccupied molecular orbitals (HOMO and LUMO), which led to a further decrease of Δ*E*_ST_ and a higher ^1^O_2_ generation rate. The cleavage of major GA proteins (p115/GM130) and fragmentation of GA structure were observed during PDT. Additionally, the C-terminal of p115 translocated into the nucleus and caused a distinct loss of mitochondrial membrane potential and a dramatic inhibition of ATP synthesis, which activated the apoptotic pathway in HeLa cells. The GA-targeting AIEgens exhibited effective suppression of tumor growth *in vivo* through PDT with no obvious side effects. Importantly, with a similar ROS generation rate, TPE-T-CPS (GA-targeting) showed higher tumor inhibition efficiency than TPE-PyT-PS (non-GA-targeting). This work represents a typical example of GA-targeting PSs based on AIEgens, which provides a strategy for precise and effective PDT.

Coinage metal clusters of Au, Ag, and Cu with a precisely defined number of constituting atoms, which can be considered as an intermediate state between discrete metal atoms and plasmonic NPs, offer small size (I the range of 1–3 nm) and compositional homogeneity similar to that of well-defined molecules.^384−388^ Their properties, such as the ultrasmall size, biocompatibility, low cytotoxicity, large Stokes shift, and NIR emission, have made metal clusters a particularly attractive category of luminescent materials.^389,390^ Importantly, these nanoclusters also experience the AIE effect able to significantly enhance their PLQY in the aggregate and solid states. Due to their atomic precision, coinage metal clusters can provide an ideal platform at the nanoscale for understanding the precise interaction mechanisms with cells and biomolecules.^391,392^ In 2020, Zang’s group reported an enantiomeric pair of propeller-like trinuclear AIE-active copper clusters [Cu_3_(R/S-BINAP)_3_CO_3_]^+^(^t^BuSO_3_) (denoted as R/S-Cu_3_) by using (*R*)- or (*S*)-2,2-bis(diphenylphosphino)-1,1-binaphthyl (R-BINAP or S-BINAP) as ligands.^393^ They displayed variable emission wavelengths after UV light irradiation, which was caused by the gradual stepwise oxidation of ligands in these clusters. Furthermore, based on the capability for producing ROS, these clusters were successfully used for lysosome-targeted PDT. Subsequently, Zang’s group reported a series of AIE-active copper clusters exhibiting intense color-tunable emissions in an oxygen-free environment or in the aggregated state.^394^ Employing the O_2_ scavenger dimethyl sulfoxide as the solvent, joint photo- and oxygen-controlled multicolor switches were realized. In this study, an aggregation-induced barrier to oxygen, an AIE mechanism for metal clusters was proposed. Moreover, imaging-guided potential PDT was realized due to the combination of the AIE effects and photoinduced ^1^O_2_ production characteristics of the copper clusters.

##### 3.1.1.2 PDT-Induced Treatment

Ferroptosis is a newly defined modality of regulated cell death caused by plasma membrane lipid peroxidation in an iron-dependent manner.^395^ Since the discovery of ferroptosis, many physiological and pathological processes undergo ferroptosis, such as CD8^+^ T cell-mediated immune attack, cardiovascular diseases, brain diseases, aging processes, etc.^396^ Therefore, understanding the mechanism and action of ferroptosis during these biological contexts might bring us perspectives for disease diagnosis and treatment.

**Figure 16:**
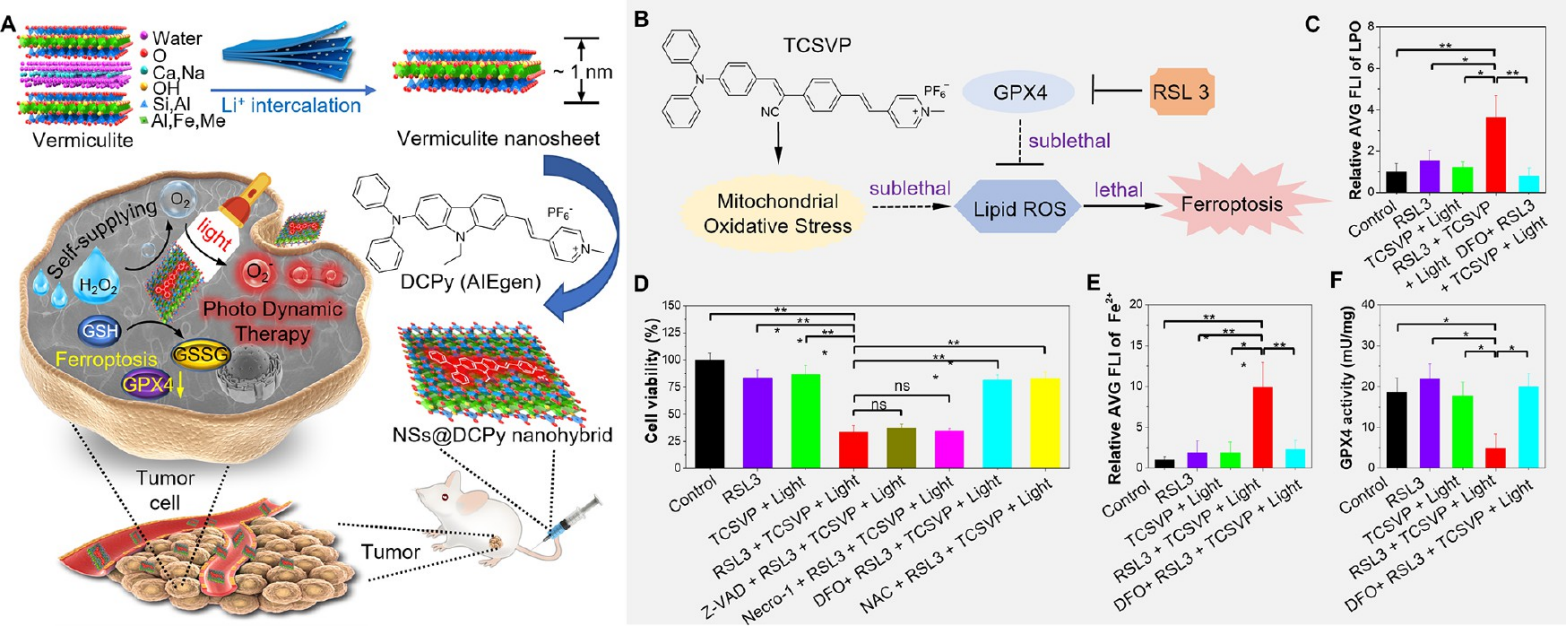
(A) Schematic illustration of ferroptosis-combined oxygen self-sufficient PDT. Adapted with permission from ref (397). Copyright 2022 Elsevier. (B) Chemical structure of TCSVP and the proposed mechanism of enhancing ferroptosis by mitochondrial oxidative stress via AIEgen treatment. (C) Lipid peroxidation (LPO) levels of PLC cells with different treatments. (D) Cell viability of PLC cells with different treatments. (E) Intracellular level of Fe^2+^ and (F) GPX4 activities of PLC cells with the corresponding treatments. Adapted with permission from ref (398). Copyright 2022 Springer Nature.

In addition to exploring the cellular dynamics of ferroptosis, AIEgens are very promising for improving ferroptosis-based cancer therapy.^42^ One efficient way to induce ferroptosis is triggering the Fenton reaction between iron and hydrogen peroxide in cells. The generated hydroxyl radicals can convert the unsaturated lipids to peroxidized lipids, which finally contribute to cell death. Based on this consequence, Tang et al. designed and prepared an AIE nanohybrid PS based on vermiculite nanosheets for ferroptosis-integrated photodynamic cancer therapy (Figure 16A).^397^ First, the nanosheet was fabricated by lithium-ion intercalation with potting soil vermiculite that contains Fe_2_O_3_, which can produce oxygen and promote the Fenton reaction to generate hydroxyl radicals by a series of catalytic reactions in the tumor microenvironment. Then, AIEgens were added to the surface of the as-prepared nanosheet by electrostatic attractions to facilitate enhanced photodynamic cancer treatment by virtue of self-generated oxygen. The antitumor experiments and the analysis of the levels of GPX4 and intracellular iron confirmed the benefits and efficiency to amplify ferroptotic cancer cell death using the combined AIEgen-based system for cancer therapy. Apart from combination with reagents that induce the Fenton reaction to enhance ferroptotic cell death, AIEgens can also trigger specific organelle oxidative stress to sensitize cancer cells to GPX4 inhibitors through an amplified ferroptosis pathway. Recently, a mitochondria-targeting AIE PS (TCSVP), capable of producing ROS efficiently under light irradiation after accumulating within mitochondria, was reported.^398^ Interestingly, the mitochondrial oxidative stress caused by TCSVP after light irradiation was sublethal to the cancer cells but could strongly stimulate the ferroptotic cell death when coadministered with RSL3, a GPX4 inhibitor also in a sublethal dose (Figure 16B). Further detailed studies of lipid peroxidation, GPX4 activity, and intracellular ferrous ion after combined administration of TCSVP and RSL3 confirmed the significant amplification of the ferroptotic cell death pathway (Figure 16C–16F). Besides, the rescue experiments using various cell death inhibitors shown in Figure 16D indicated that the combined killing effect on cancer cells by the two sublethal treatments is mediated by the ferroptosis pathway instead of by apoptosis or necrosis, which clearly illustrates the importance of AIE PSs in ferroptosis cancer therapy.

Immunogenic cell death (ICD) is a type of apoptosis accompanied by the release of damage-associated molecular patterns, such as outer-membrane-located calreticulin, high mobility group protein B1 (HMGB1), and heat shock protein70 (HSP70). These agents could further stimulate the antigen presentation by dendritic cells to T cells and activate a systematic antitumor immune attack, greatly contributing to the suppression of tumor growth or recurrence for a long time after stopping treatment.^399^ Because of the strong immunogenicity of dying cancer cells undergoing the ICD process, ICD induction has served as a promising method to convert an immunologically cold tumors that lacks T cell infiltration into a hot tumors with large infiltration of T cells, which makes the tumor more sensitive to immunotherapy.^400^ PDT is an efficient approach to induce ICD for antitumor immunotherapy.^401,402^

**Figure 17:**
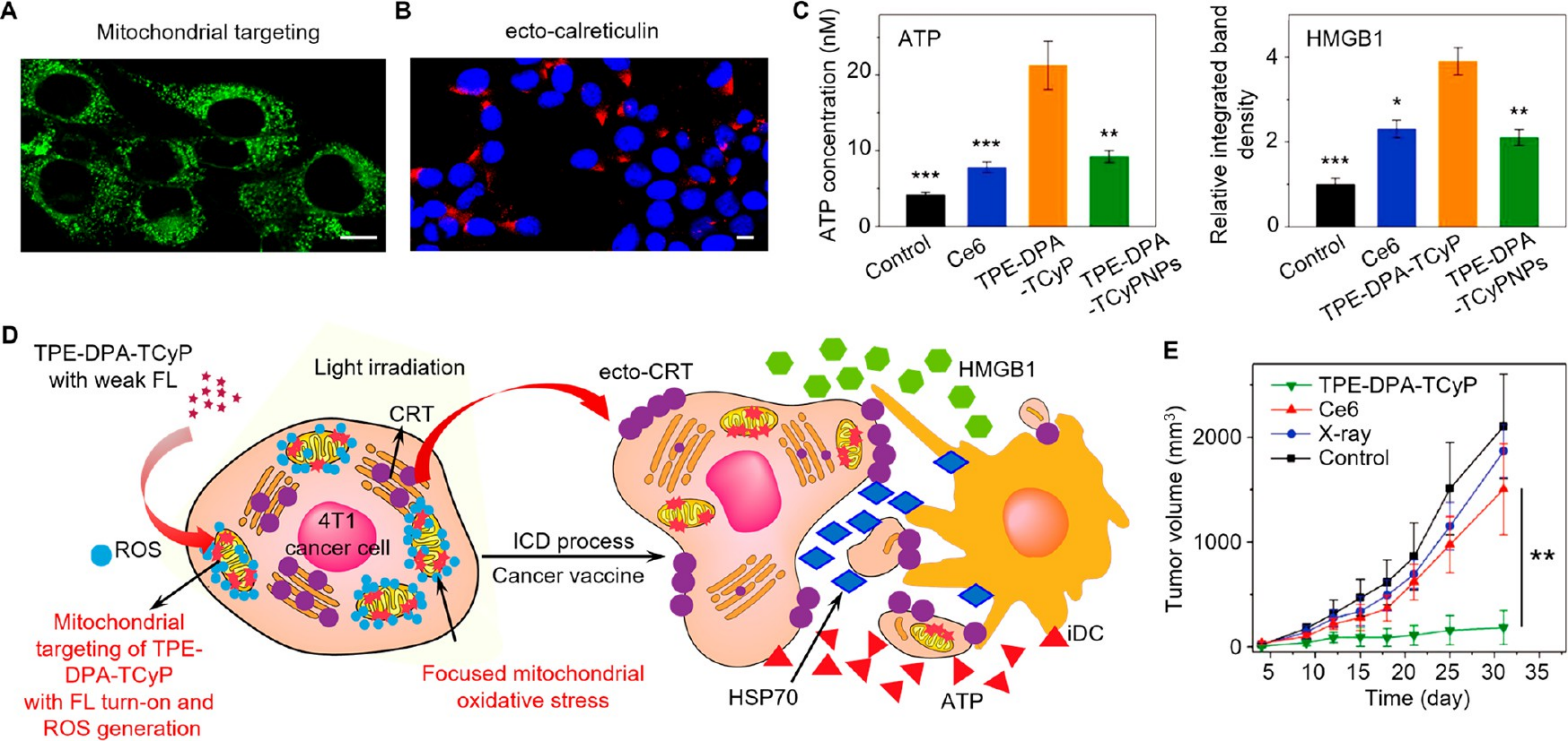
(A) CLSM image of 4T1 cancer cells stained with TPE-DPA-TCyP. Scale bar: 10 μm. (B) Representative CLSM image of ecto-calreticulin expression after TPE-DPA-TCyP PDT treatment (DAPI; blue fluorescence). Scale bar: 10 μm. (C) Released ATP and HMGB1 levels of 4T1 cancer cells with different treatments. (D) Schematic illustration of ICD initiation by focused mitochondrial oxidative stress via TPE-DPA-TCyP. (E) Tumor volume during the vaccine experiment. Adapted with permission from ref (403). Copyright 2019 Wiley-VCH.

In 2019, Ding et al. reported that mitochondrial oxidative stress caused by an AIE PS can efficiently induce ICD.^403^ They designed an AIE PS named TPE-DPA-TCyP exhibiting a twisted molecular skeleton with a typical D-π-A structure and a positive charge that conferred the AIE PS with a mitochondrial targeting ability. When TPE-DPA-TCyP was dissolved in PBS, excited-state energy was released by nonradiative decay, leading to a very weak emission. After binding to lipid vesicles (mimicking the mitochondria outer membrane) by charge attraction, intramolecular motion and the TICT process were restricted, resulting in a blue-shifted turn-on fluorescence emission. With the specific targeting and lighting ability to mitochondria, the ROS generation efficiently caused mitochondrial oxidative stress and further induced immunogenic cancer cell death, which was determined by the key markers of ICD such as ecto-calreticulin, ATP and HMGB1 (Figure 17A–17D). Furthermore, a prophylactic tumor vaccination experiment was performed to affirm the ICD effect induced by TPE-DPA-CyP (Figure 17E). Since, this work determined the relationship between mitochondrial oxidative stress and ICD.

In addition to mitochondrial stress, other organelle-related stresses have also attracted extensive attention for effectively inducing ICD. ER-targeting AIE probes were synthesized for highly efficient ICD induction.^404^ The probes were constructed by covalently coupling a TPE-based AIE PS and an ER-targeting peptide. After demonstrating the ER-targeting ability and ER-stress induction at the cellular level, the authors further confirmed the ICD effects induced by the probes using a vaccine experiment *in vivo*, showing better ICD induction than that of hypericin-based PDT (a widely recognized ER-targeting ICD inducer) under the same conditions. Furthermore, in 2021, Ding et al. reported a lysosomal membrane permeabilization inducer can be serve as an efficient ICD inducer and convert immunologically cold tumors into hot ones.^405^

#### 3.1.2 Combined Therapy

Currently, various cancer treatments, such as PDT, chemotherapy, radiotherapy, PTT, immunotherapy, etc., have exhibited high anticancer efficacy in laboratory research or clinical practice with great success in suppressing tumor proliferation.^365,406,407^ However, very often a single treatment modality cannot completely eliminate the whole tumor, and it may be ineffective in preventing cancer metastasis, which is attributed to the complexity, diversity, and heterogeneity of tumors.^408,409^ To overcome these obstacles of monotherapy, combined therapy, referring to the integration of two or more forms of treatment, has been proposed as an alternative approach to cancer therapy.^410−413^ Indeed, multimodal synergistic therapy can produce “superadditive” effects through cooperative interactions and enhancement among several types of monotherapies, resulting in a stronger therapeutic effect when compared to a single therapy.^414,415^ Therefore, the current cancer treatment trend has gradually shifted from monotherapy to multimodal treatment for enhanced treatment efficacy.^336,416−419^ Most of the multimodal phototherapeutic agents, such as inorganic materials and all-in-one materials, have been developed by integrating different functional components into a nanoplatform. Although these materials can partially satisfy multimodal diagnosis and therapy, there are still some problems such as complexity of the components, low reproducibility, difficulty in processability, and undesired ACQ effects. In this regard, organic AIEgens could be powerful candidates for combined therapy by virtue of their well-defined chemical/molecular structures, simple preparation, good reproducibility, ease of processability, and high brightness.

##### 3.1.2.1 NIR-Guided Phototheranostics

Phototheranostics, which integrates optical imaging diagnostics and phototriggered precision therapeutics, is a rapidly developing area of biotechnology. As the kernel of phototheranostic systems, the development of high-performance phototheranostic agents is crucial to the progress of phototheranostic research.^420,421^ NIR-II AIEgens-guided phototheranostics has attracted enormous attention for precise cancer treatment by virtue of a real-time diagnosis and concurrent *in situ* therapy upon light irradiation.

**Figure 18:**
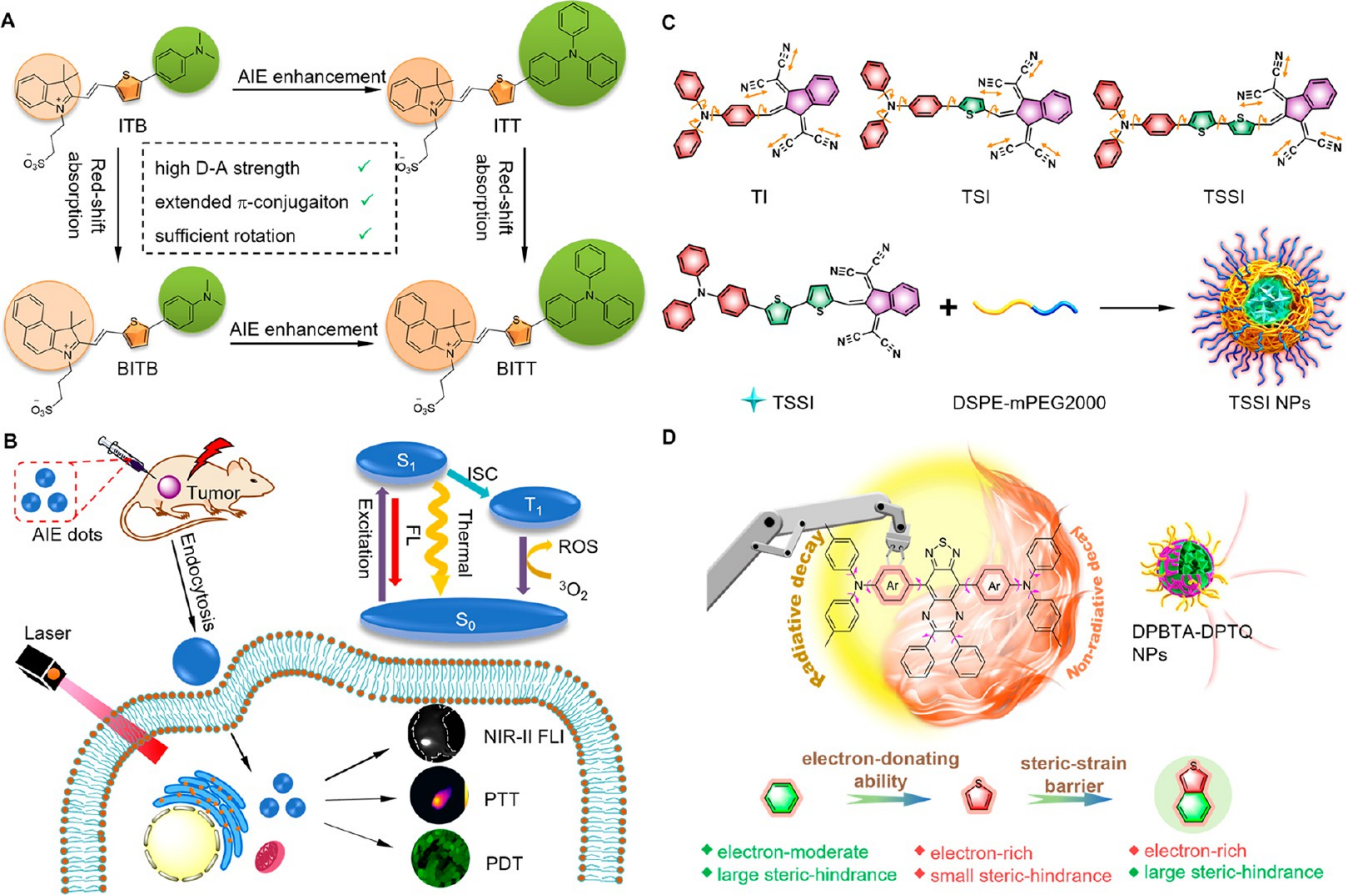
(A) Molecular design of ITB, ITT, BITB, and BITT. (B) Schematic illustration of NIR-II FLI-guided synergistic PDT-PTT phototherapies with BITT dots. Adapted with permission from ref (422). Copyright 2020 Wiley-VCH. (C) The molecular design (TI, TSI, and TSSI) and illustration of the formation process of TSSI NPs. Adapted with permission from ref (425). Copyright 2020 Wiley-VCH. (D) Schematic illustration of the design principle of NIR-II AIEgens by D/π-bridge manipulation as well as the corresponding construction and photoexcitation of DPBTA-DPTQ NPs. Adapted with permission from ref (428). Copyright 2021 Wiley-VCH.

Rational molecular design and facile synthetic strategies are required to tune the emission wavelength of AIEgens to the NIR-II region because most NIR-II AIEgens are constructed by enhancing the D-A effect.^422−424^ By integrating D-A enhanced and extending π-conjugation into a propeller-shaped molecule, a series of NIR-emissive zwitterionic AIEgens (ITB, ITT, BITB, and BITT) were obtained (Figure 18A).^422^ Interestingly, the target molecule BITT not only exhibits strong NIR-II emission due to enhanced high D-A strength and twisted rotor structure but also provides a satisfactory ROS generation capability and high photothermal conversion efficiency (PCE) for synergistic PDT-PTT phototherapy (Figure 18B). This study thus provided a groundbreaking tactic for constructing multimodal AIE phototheranostic systems using a single molecular species via combining various imaging technologies with different therapeutic methods. Accordingly, one-for-all phototheranostics, enabling simultaneous multimodal imaging and synergistic phototherapy, have been realized achieve the optimum use of dissipated energy by ingeniously regulating the balance between radiative and nonradiative decays.^425^ As illustrated in Figure 18C, three AIE compounds (TI, TSI, and TSSI) were designed with the 1,3-bis(dicyanomethylidene)indane moiety serving as a strong electronic acceptor and vibrator. Owing to the advantages of extended D–A strength, twisted conformation, intramolecular motion, and vibration in the aggregate state, the TSSI NPs balanced the energy dissipations between radiative and nonradiative decay and then provided efficient ROS generation and high PCE (46.0%) simultaneously as well as bright fluorescence in the NIR-II window. PDT-PTT synergistic therapy was realized after injection of TSSI NPs, resulting in precise tumor diagnosis and complete tumor elimination after a single irradiation. Inspired by the powerful all-round phototheranostic agent based on a single AIEgen, other AIE theranostic agents with one-for-all features were also developed to further facilitate precise cancer diagnosis and treatment. Owing to the small Δ*E*_ST_ caused by high D-A strengths, rotor-like twisted and charged structures, extraordinary photosensitizing properties were realized for trimodal image-guided PDT-PTT therapy with a single phototheranostic agent (TTT-4 dots), allowing intense NIR emission, high ROS generation performances, and high PCE (39.9%).^426^ For pancreatic cancer, known as the king of cancers, an AIE-active one-for-all phototheranostic agent (DCTBT NPs) has been developed for high-quality diagnosis and synergistic phototherapy, including NIR-II fluorescence imaging, type-I PDT, and PTT functions.^427^ Stable NIR-II AIEgens with balanced phototheranostic performances were also obtained by implementing precise D/π-bridge manipulation.^428^ As shown in Figure 18D, the conjugation length and distorted backbone were both enlarged by employing electron-rich benzo[*c*]thiophene as the bulky D/π-bridge, bringing about a favorable design principle for D-π-A-π-D type NIR-II AIEgens (DPBTA-DPTQ). Benefiting from the acceptable NIR-II PLQY (0.45%) and notable PCE (40.6%), multimodal imaging was achieved in HepG2 and B16–F10 tumor-bearing mice to guide the photothermal tumor ablation. These outputs confirm that D/π-bridge manipulation is an innovative molecular design strategy to construct multifunctional NIR-II AIEgens as high-performance theranostic agents.

**Figure 19:**
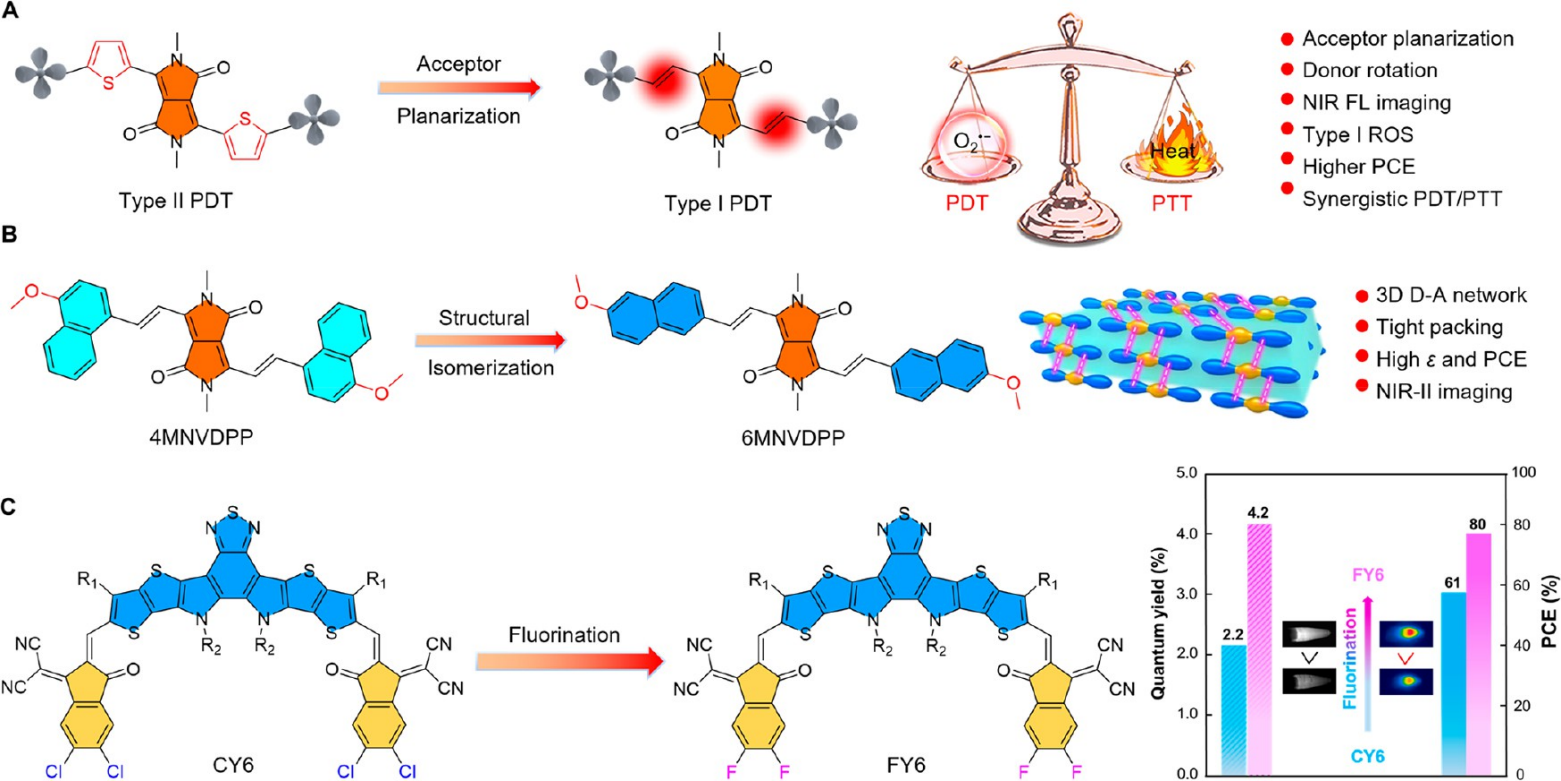
(A) Schematic illustration of molecular engineering strategies used to design type I PSs and photothermal reagents. Adapted with permission from ref (429). Copyright 2022 American Chemical Society. (B) Schematic illustration of design strategy for NIR-II phototheranostic agents by controlling molecular packing of 4MNVDPP and 6MNVDPP. Adapted with permission from ref (430). Copyright 2022 Wiley-VCH. (C) Schematic demonstration of fluorination strategy for designing A-D-A type NIR-II photothermal agents with synchronously improved QY and PCE. Adapted with permission from ref (431). Copyright 2023 Wiley-VCH.

Given that the therapeutic effectiveness of type II PSs is highly dependent on the surrounding oxygen concentration, Wang et al. proposed a strategy of the acceptor planarization and donor rotation to construct AIE-active PSs with both type I ROS generation and high PCE (Figure 19A).^429^ After replacing the thiophene linker in the 3- and 6-positions of diketopyrrolopyrrole (DPP) derivatives with a vinyl unit, three AIEgens (TPAVDPP, TPATPEVDPP, and 2TPEVDPP) were obtained via Knoevenagel condensation, in which triphenylamine or TPE was used as the donor moiety. The planarization induced by the double bond helped enhance intramolecular D-A interactions, diminished the energy gap between the HOMO and LUMO, and promoted red-shifted absorption, which transferred the type of the PSs pathway. Two highly twisted TPE donors could effectively block the intermolecular π-π interactions to maintain AIE, and at the same time, tune the nonradiative decay channels to endow high PCE. As a consequence, 2TPEVDPP NPs provided a good balance between ROS generation and thermal dissipation pathways (PCE = 66%), and realized NIR-I imaging-guided tumor ablation. Based on the same DPP acceptor unit, two isomers (4MNVDPP and 6MNVDPP) were synthesized (Figure 19B).^430^ Interestingly, the two isomers exhibited completely different stacking patterns in their single crystal structures. 6MNVDPP formed a 3D spatial D-A interlocked network, while 4MNVDPP exhibited 2D D-D type *J*-aggregates, which was attributed to the larger surface electrostatic potential difference of 6MNVDPP and the resulting strong intra- and intermolecular D-A interactions. Therefore, after encapsulated into NPs, the maximum absorption wavelength of 6MNVDPP NPs was red-shifted by 178 nm and exhibited a molar extinction coefficient of up to 3.71 × 10^4^ M^–1^·cm^–1^, and the emission wavelength was extended to the NIR-II region when compared with 4MNVDPP NPs. In addition, 6MNVDPP NPs exhibited excellent ROS generation, high PCE (89%), and good photothermal stability. The excellent photophysical properties of 6MNVDPP NPs enabled NIR-II fluorescence imaging-guided photodynamic/photothermal synergistic treatment of tumor tissues. Recently, given the contradiction between PLQY and PCE, which are two important parameters of NIR-II phototheranostic agents, a fluorination strategy was shown to enhance NIR-II PLQY and the PCE of phototheranostic agents concurrently by taking advantage of the small atomic radius, large electronegativity, low photon energy of fluorine atoms and high polarizability of the C–F bond (Figure 19C).^431^ As a proof-of-concept, a series of fluorinated or chlorinated phototheranostic agents were designed and synthesized. As expected, the NPs of all the fluorinated phototheranostic agents exhibited higher molar extinction coefficients, NIR-II PLQY and photothermal effect compared with their chlorinated counterparts. Among those agents, FY6-NPs exhibited the highest NIR-II PLQY (4.2%) and PCE (80%), which endowed FY6-NPs with excellent vascular imaging capabilities and enabled NIR-II imaging-guided photothermal ablation of tumors. This study provided valuable guidance for constructing NIR-II phototheranostic agents with high performance.

**Figure 20:**
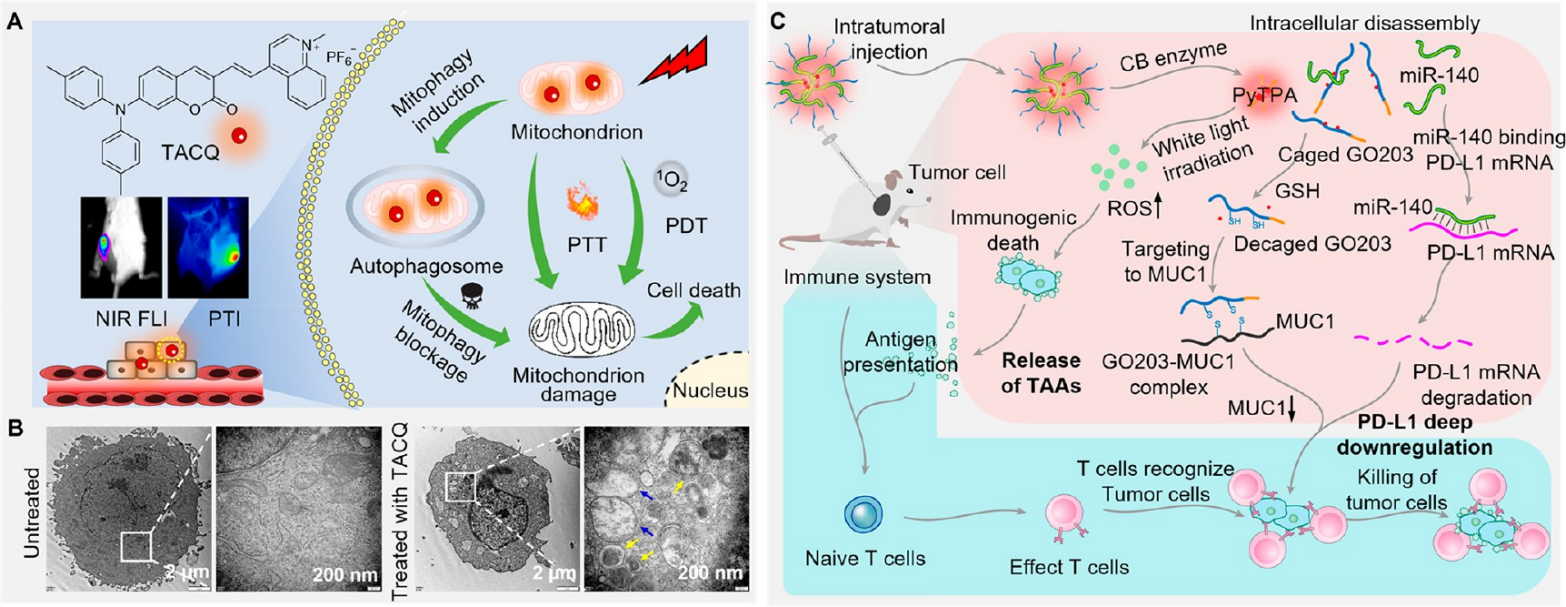
(A) Chemical structure of TACQ and its bioapplications. (B) TEM images of HeLa cells untreated/treated with TACQ. Adapted with permission from ref (432). Copyright 2021 American Chemical Society. (C) The processes of GCP/miR-140 NPs deep down-regulate the PD-L1 expression. Adapted with permission from ref (442). Copyright 2022 Wiley-VCH.

Mitophagy, a kind of mitochondria-related autophagy, plays a vital role in the energy balance and material metabolism. Mitophagy is considered to be a potential therapeutic target, since the mitophagy level in cancer cells is higher than that in normal cells. Recently, Zhao et al. developed a cationic AIEgen (TACQ) with mitophagy regulating capability for synergistic cancer theranostics (Figure 20A).^432^ The strong push-pull effect and twisted molecular conformation enabled TACQ to exhibit typical AIE characteristics and emit in the NIR-II region (>1000 nm). Upon irradiation with a 635 nm red laser, excellent photothermal efficiency of TACQ in the aggregated state with a PCE of 55% was obtained. Besides heat generation, TACQ also efficiently produced ^1^O_2_. Thanks to its lipocationic nature, TACQ selectively resided in cancerous mitochondria rather than in normal cells, even in complex environments. After HeLa cells were treated with TACQ, swollen mitochondria and double-membrane vesicles were observed, suggesting the occurrence of mitophagy (Figure 20B). Unexpectedly, TACQ could not only induce mitophagy but also block the mitophagic flux, which was confirmed using mCherry-GFP-LC3B staining experiments. This mitophagy-regulating capability increased the number of damaged mitochondria and led to cell death. Given the synergistic therapeutic effects of mitophagy blockage, PTT, and PDT, TACQ exhibited excellent photocytotoxicity against cancer cells.

Apart from molecular engineering, supramolecular complexes have been constructed for use in cancer theranostic applications. Qin et al. demonstrated a prism-like supramolecular metallacage C-DTTP with efficient NIR-II fluorescence through the assembly of a four-arm AIE ligand with Pt acceptors.^433^ C-DTTP exhibited a long emission wavelength (λ_max_ = 1005 nm) and displayed excellent biocompatibility, superior ROS generation behavior, and PCE of 39.3%. By means of the synergistic PDT/PTT therapy, precise tumor diagnosis and effective tumor elimination were afforded simultaneously, which should encourage the development and applications of fluorescent supramolecular theranostics. Pillar[*n*]arenes are an emerging class of macrocycles with many structural advantages and host-guest properties.^434,435^ Importantly, the convenient substitution on the symmetric rims of pillar[*n*]arenes not only enables their complexation with various AIEgens for enhanced emission but also enables hybridization with inorganic materials such as mesoporous silica NPs, metal-organic frameworks, and metallic nanomaterials, thus generating combined functions of both the supramolecular compositions and inorganic entities. Taking advantage of these characteristics, multifunctional theranostic systems have been successfully fabricated. For instance, pillar[5]arene-modified gold nanorods (AuNRs) loaded with AIEgens via host-guest interactions as the PS were prepared, which exhibited multiple modalities including desirable photothermal effect for PTT, high ROS generation capacity for PDT, fluorescence imaging, and PAI for imaging-guided treatment.^436^ In the preparation step, carboxylatopillar[5]arene (CP5)-modified AuNRs were synthesized via a ligand-exchange process, and the AIE-active PS, denoted as TTPY-Py, was encircled with CP5 through host-guest complexation. On the one hand, the fluorescence of TTPY-Py was dramatically enhanced upon forming host–guest interactions with CP5 due to the RIM mechanism. On the other hand, the release of TTPY-Py could be efficiently triggered by multiple external stimuli, including NIR irradiation, lowering the pH, and raising the temperature. The dissociation between TTPY-Py and CP5 was followed by the escape of the PS from the nanomaterial to the mitochondria, where TTPY-Py accumulated because of its positively charged pyridinium terminals and then triggered the generation of ROS under white light. In addition, apart from AIEgen release, NIR irradiation could also initiate the photothermal conversion activity of AuNRs. Accordingly, combined theranostic efficacy integrating multiple therapeutic performances (PTT and PDT) was successfully activated under the synergistic effect of these stimulating factors, which is highly beneficial for promoting noninvasive and accurate cancer treatment.

##### 3.1.2.2 Photodynamic-Immunotherapy

Although AIEgen-based PDT has attracted significant recent attention, the limited light penetration depth and specific location for light activation have led to difficulties in using PDT for treating deep-seated or metastatic tumors. On the other hand, immunotherapy such as immune checkpoint blockade (ICB) therapy could activate the T cells, and alleviate the immunosuppressive microenvironment, eventually boosting antitumor immune response for the whole body.^437−439^ However, although promising, this treatment currently only benefits a minority of cancer patients whose tumors have been preinfiltrated by T cells. Considering that PDT could result in an immunogenic tumor microenvironment, the combination of PDT and immunotherapy has the potential to treat diverse difficult-to-treat tumors; as such, some research on this topic has been reported. And Lou et al. proposed that immune the response procedures were a series of step-by-step links, and if any hindrance arises, the immunity cycle could be halted, and obviously, the immunotherapy effect would be discounted.^440^ Thus, based on the whole cancer-immunity cycle, they reported an immunotherapy strategy and constructed a cascade amplification nanocomposite (PMRA/Poly (I:C)) to achieve a continually reinforcing immune response through strengthening every step in the whole immunity cycle.^441^ Based on the current single down-regulation strategy, Lou et al. designed the caged peptide-conjugated AIEgen/miR-140 NPs to deep down-regulate the PD-L1 expression and achieve enhanced PDT/immunotherapy (Figure 20C).^442^ The caged peptide-conjugated AIEgen probe (GCP) consisted of three parts: caged GO203 peptide, cathepsin B (CB) enzyme cleavable peptide, and PyTPA. GCP with a hydrophobic aromatic structure and positively charged side-chain was self-assembled with negatively charged miRNA into GCP/miR-140 NPs. After internalization by tumor cells, the structure of GCP/miR-140 could be broken by the overexpressed CB enzyme, releasing miR-140 and the caged GO203 peptide. Then, the high glutathione (GSH) concentration in tumor cells removed the tert-butyl mercaptan group on caged GO203, exposing the active thiol on the CQC sequence. Decaged GO203 peptide could then inhibit the homodimerization process of mucin 1 (MUC1), thus downregulating PD-L1 expression. At the same time, miR-140 could bind to the PD-L1 messenger RNA (mRNA) and prevent the translation process of PD-L1. *In vivo* experiments proved that after down-regulating PD-L1 expression in tumor cells using two methods and enhancing antigen presentation via PyTPA-PDT, antitumor immunity could be activated effectively, thereby realizing enhanced immunotherapy.

**Figure 21:**
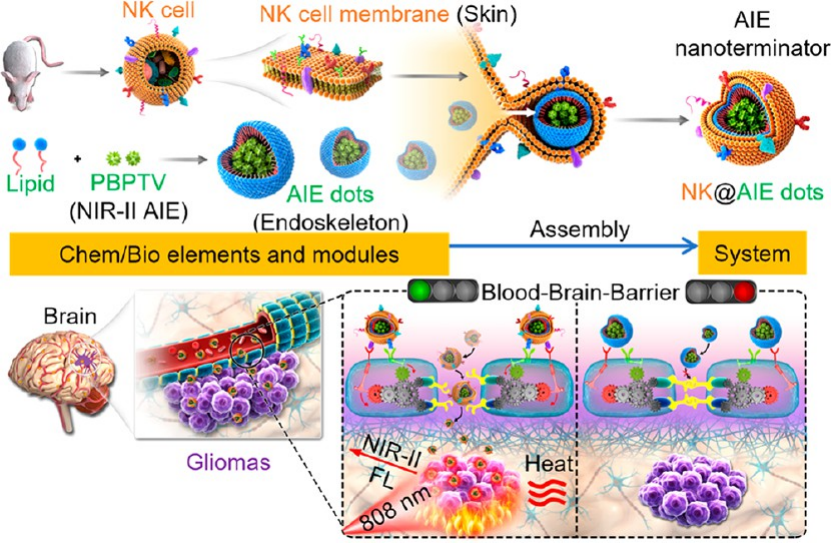
Schematic illustration of the preparation and assembly process of NK-cell-mimic AIE nanoparticles (NK@ AIEdots) and the “smart” tight-junction (TJ)-modulated blood brain barrier penetration of NK@AIEdots for brain tumor targeted light-up and inhibition. Adapted with permission from ref (444). Copyright 2020 American Chemical Society.

Natural cell membrane-coated NPs have arisen as a biomimetic theranostic platform for cancer therapeutic applications. These NPs were inspired by the structure of natural cell membranes, which inherit the membrane protein profile from the source cells, enabling them to act as seemingly cells that can deliver their cargo to the target site and activate immune-associated cells. Natural cell membrane-coated NPs strike a chord with the concept of “terminators”, killing machines with living tissue over a robotic endoskeleton, which inspired researchers to develop a nanoscale “terminator” for cancer eradication. Immune cells are a crucial component of the precise treatment of cancer. Some complex proteins are distributed on the immune cell membrane, which helps to improve the recognition and targeting of immune cells to tumors and enhance the antitumor immunity of immune cells. Moreover, there is evidence that the immune cell membrane exhibits a certain antitumor immunotherapy function. Based on this characteristic, researchers have attempted to transplant the properties of immune cells on AIE nanoaggregates to produce immune cell-like nanoterminators for tumor targeting and therapy through cell membrane coating technology.^443^ Natural killer (NK) cells are lymphocytes of the innate immune system that are involved in tumor immunosurveillance. Born to kill, these cells are manufactured in the bone marrow, circulate in the blood, and identify abnormal cells with the help of receptors expressed on their cell membrane. NK cells exhibit various proteins for tumor recognition and have been used in cancer immunotherapy. The natural killer cell membrane was wrapped on AIE polymeric nanoaggregate to produce an NK-cell-like nanoterminator (NK@AIEdots) (Figure 21).^444^ The as-prepared NK@AIEdots with bright NIR-II fluorescence could not only cross the blood-brain barrier but also specifically accumulate in brain tumors within the complex brain matrix for further *in situ* through-skull-scalp imaging and PTT. Dendritic cells (DC) are “professional” antigen-presenting cells that can prime T cells. Research suggests that mature DCs could induce potent antitumor T cell immunity. The priming of T cells by mature DCs was mainly through cell membrane surface components, which may be important for highly functional therapeutic T cell generation. The DC cell membrane was coated on the nanoaggregate formed by AIE PSs to produce a DC-cell-like nanoterminator (DC@AIEdots) for cancer photodynamic and photothermal immunotherapy.^445^ Notably, the inner AIE PSs could selectively accumulate in tumor cells for PDT, and the outer cell membrane could facilitate DC@AIEdots to “hitchhike” on endogenous T cells in a modular manner and stimulate T cell proliferation and activation. The tumor delivery efficiency of PSs increased from 25.12% to 39.77% (i.e., about 1.6 times) by leveraging T cells. The combination of the LDs targeting PDT and artificial antigen-presenting to T cells can effectively reduce the size of the tumor *in situ* and evoke the whole immune system to inhibit the growth of both primary and distant tumors with a long-lasting effect. This study not only confirms the great potential of LDs as targeting therapeutic agents for tumor microenvironment modulation but also provides a photoactive antigen-presenting platform with abilities for highly efficient drug delivery and cancer photon-immunotherapy.

##### 3.1.2.3 Photodynamic-Chemotherapy

Cancer therapies usually suffer from a poor targeting ability and serious side effects. Photoactivatable cancer therapy offers the significant advantages of high spatiotemporal resolution and can be monitored *in situ* by fluorescence imaging, which is promising for precise cancer treatment.^446^ Gao and co-workers developed dual organelle-targeted NPs with synergistic chemo-PDT functions through self-assembly of mitochondria-targeted chemotherapeutic agent AIE-Mito-TPP and lysosomes-targeted PS AlPcSNa_4_.^447^ These dual organelle-targeted NPs could be quickly taken up by cancer cells through endocytosis and gradually decompose to release AIE-Mito-TPP and AlPcSNa_4_, which accumulated in the mitochondria and lysosomes. The AIE-Mito-TPP efficiently destroyed mitochondrial functions by disrupting mitochondrial membrane potential and inhibited ATP synthesis, while the AlPcSNa_4_ efficiently destroyed lysosomes via ROS generation under NIR light irradiation. The dual organelle-targeted drug delivery process can also be self-monitored by the dual light-up fluorescence of green-emissive AIE-Mito-TPP and red-emissive AlPcSNa_4_. Under NIR light irradiation, the AIE-Mito-TPP/AlPcSNa_4_ NPs display cytotoxicity against A375 cells and efficient inhibitory ability for *in vivo* tumor growth, which is promising for image-guided precise cancer therapy. Prodrugs which can be selectively activated in cancer cells are ideal agents for cancer therapy due to their improved tumor specificity and minimized side effects. Recently, an esterase-responsive prodrug (TPE-QC) was developed, which was constructed using the anticancer drug of chlorambucil and an TPE-QO AIEgen using a hydrolyzable ester linkage.^448^ The fluorescence and photosensitization of TPE-QC were quenched due to the photoinduced electron transfer (PET) process. However, the ester group of TPE-QC could be hydrolyzed by esterase to release TPE-QO and chlorambucil, which blocked the PET process and resulted in the recovery of both fluorescence and photosensitization. Benefiting from the higher levels of esterases in cancer cells, TPE-QC could be selectively activated in cancer cells over normal cells. The activated TPE-QC was mainly located in the mitochondria and lysosomes, as indicated by the enhanced fluorescence signals. As expected, TPE-QC exhibited higher photocytotoxicity for cancer cells under white light irradiation. Importantly, TPE-QC could be successfully activated at the tumor site and efficiently inhibited tumor growth through synergetic PDT and chemotherapy.

### 3.2 Bacterial Elimination

Bacterial infections are the most prominent underlying reasons for many severe diseases, such as pneumonia, sepsis, septic arthritis, and inflammatory bowel disease, which have increasingly raised medical and public concerns across the world.^449^ There is also increasing evidence that bacteria can also indirectly promote the occurrence and progression of other diseases (e.g., cancer) and compromise the efficiency of cancer chemotherapy and immunotherapy.^450^ Since the discovery of penicillin, antibiotics have emerged as the most important weapon for humans against bacterial infections. However, such antibacterial abilities have been dramatically impaired due to the emergence and dissemination of antibiotic resistance. Increasing efforts are devoted to bacterial elimination and infection treatment to alleviate or resolve these severe issues.^451,452^

Deep into the threats of bacterial infections in tissue, Liu and Kong et al. reported a strategy using metal-organic framework (MOF) NPs as the nanocarrier for precise metabolic bacterial labeling and killing *in vivo*.^453^ After intravenous injection, these NPs accumulated preferentially and degraded rapidly within the inflammatory environment, releasing encapsulated 3-azido-d-alanine (D-AzAla) in the process. Subsequently, D-AzAla was selectively integrated into the cell walls of bacteria. Ultrasmall PS-active AIE dots were delivered to react with the modified bacteria through *in vivo* biorthogonal chemical reactions. Upon light irradiation, the bacteria on the infected tissue were ablated by the efficiently generated ROS, indicating a promising strategy for precise bacterial detection and killing guided by fluorescence imaging. The exponential growth of bacteria promotes biofilm formation, whose existence provides a physical barrier to prevent the penetration of antibiotics but also utilize enzymes to degrade or absorb antibiotics and make them inactive. To address this issue, Zhao and co-workers designed a cationic antibacterial agent based on phosphindole oxide with AIE properties and efficient ^1^O_2_ generation capability.^454^ As such a visual diagnosis of bacterial biofilms could be carry out. In addition, owing to the synergistic effect of phototoxicity and dark toxicity, superb antibacterial and antibiofilm performances against multidrug-resistant Gram^+^ bacteria was achieved. Polymeric AIE molecules are reported to exhibit much higher efficiency in intersystem-crossing and triplet exciton generation, leading to ROS generation.^455^ A conjugated AIE polymer with benzothiadiazole and TPE units was developed for reliable bacterial eradication, showing much higher ROS generation efficiency compared to its monomer counterpart and commercial PS.^456^ Effective inhibition of bacteria growth was achieved under light irradiation both *in vitro* and *in vivo*, with noteworthy specificity and biocompatibility. In addition to AIEgens, researchers have found that some natural products can kill bacteria efficiently by PDT using inherent AIE features.^457,458^

Apart from these photodynamic-active AIE PSs, the existence of molecular rotors in the AIEgen skeleton could facilitate excited-state energy dissipation via nonradiative decay and thus promote photothermal conversion.^72,459−461^ Combining these features, a nanofibrous membrane, namely, TTVB@NM, was developed for synergistic photodynamic and photothermal treatment making full use of both ROS and heat. Constructed using an AIE unit, TTVB as the dopant, and an electroactive polymer PVDF-HFP as the matrix. The multilayered porous structure of TTVB@NM favored the interception of pathogenic droplets and aerosols. Upon sunlight irradiation, the generated massive ROS played a dominant role in microbicidal activity, and a moderate photothermal conversion activity supplemented the microbal inhibition, synergistically endowing the effective application for the inhibition of bacteria.^459^

Recent studies have revealed that most invasive pathogens can survive inside host cells during traditional antimicrobial treatments. Due to the challenges associated with detecting shielded intracellular bacteria and their high resistance to antibiotic therapies, traditional antimicrobial reagents have difficulties in treating these refractory infections.^462,463^ Some strategies have thus been developed based on AIEgens to image and eliminate bacteria that reside inside host cells, which include (i) one-step metabolic labeling methods, (ii) bioresponsive labeling methods, and (iii) direct labeling methods.

**Figure 22:**
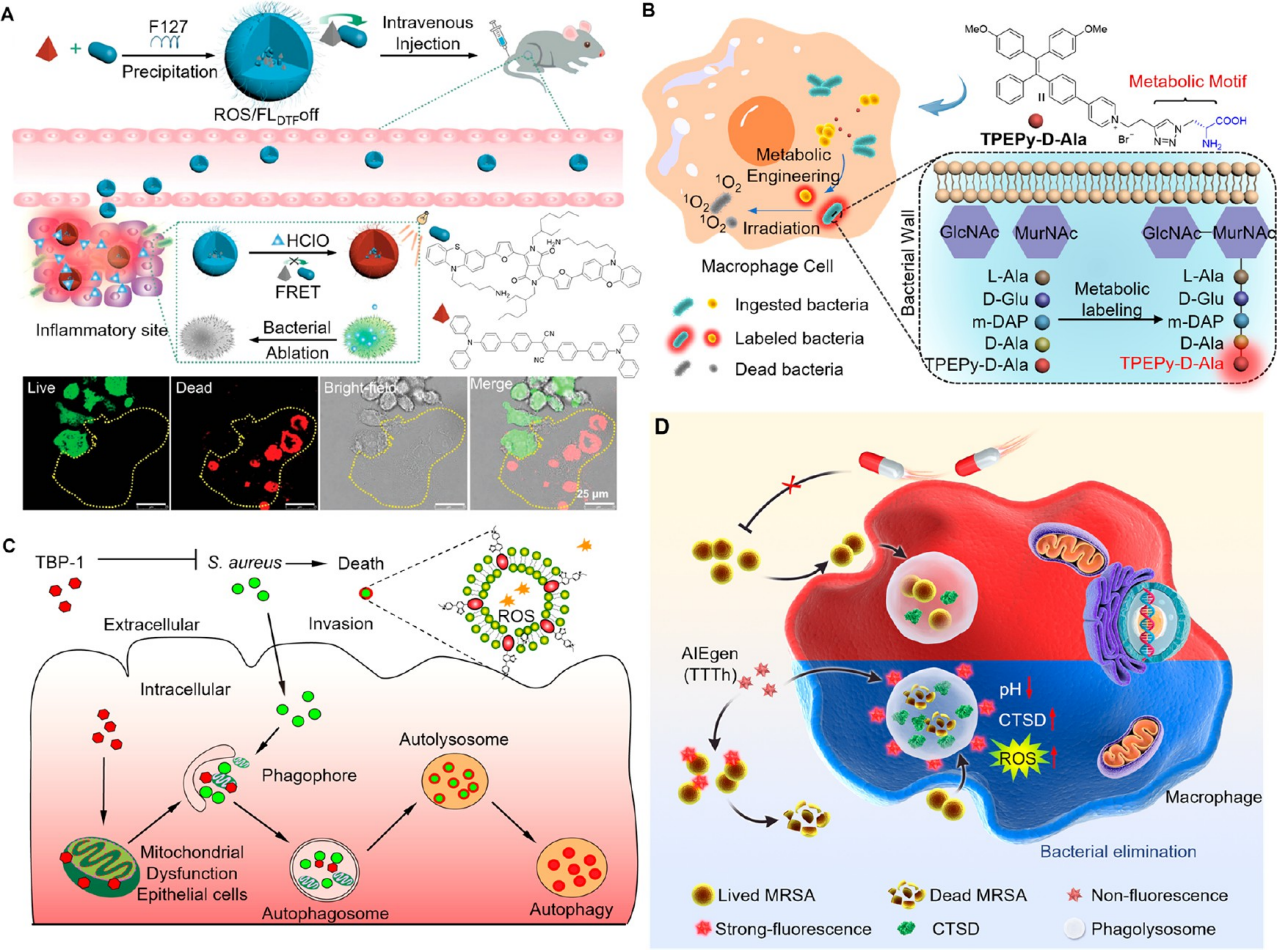
(A) Schematic of DTF-FFP NPs activated by HClO produced by phagocytes to effectively image and ablate the bacteria inside phagocytes. Adapted with permission from ref (464). Copyright 2020 Wiley-VCH. (B) Schematic representation and confocal images of TPEPy-d-Ala for detecting and ablating intracellular bacteria. Adapted with permission from ref (470). Copyright 2020 Wiley-VCH. (C) After binding with the bacterial membrane phospholipids, TBP-1 killed extracellular *S. aureus* by inducing ROS generation. Meanwhile, TBP-1 induced the autophagy of epithelial cells through regulating mitochondria to accelerate the clearance of intracellular *S. aureus*. Adapted with permission from ref (472). Copyright 2021 Wiley-VCH. (D) TTTh achieved a high photodynamic killing efficacy toward both extracellular and intracellular *S. aureus* by disrupting the bacterial membrane integrity and inducing the lysosomal maturation. Adapted with permission from ref (474). Copyright 2022 Wiley-VCH.

Bacterial walls are composed of peptidoglycans with repeating units of *N*-acetylglucosamine and *N*-acetylmuramic acid. Metabolic precursors incubated with bacteria are expressed on the bacterial cell walls, which offers a promising approach for the detection of bacteria detection based on bacterial peptidoglycan imaging. Liu and co-workers developed a one-step metabolic labeling probe TPEPy-d-Ala for *in situ* visualization and ablation of intracellular bacterial pathogens (Figure 22A).^464^ TPEPy-d-Ala showed a weak emission in aqueous media because of the good water solubility of the d-Ala and pyridinium units in TPEPy-d-Ala. By incorporation of TPEPy-d-Ala into bacterial peptidoglycan through metabolic processes, the fluorescence of TPEPy-d-Ala was enhanced because of inhibited intramolecular motion, which enabled the detection of intracellular bacteria. The probe could selectively label the peptidoglycan of intracellular bacteria but not the membrane of the host cells. Moreover, TPEPy-d-Ala could ablate intracellular bacteria *in situ* by photodynamic treatment, which was more effective than using vancomycin, a common antibiotic. Subsequently, another one-step metabolic probe, TPACN-d-Ala, was developed.^465^ By virtue of its superior water solubility, TPACN-d-Ala could circulate in blood when intravenously injected into mice and was able to diffuse well into the host cells or biofilms to be involved in the biosynthesis of peptidoglycan. TPACN-d-Ala specifically illuminated the *in vivo* bacterial wall to track and ablate the sheltered bacteria effectively through PDT.

Although the metabolic labeling method showed high specificity for imaging and ablating bacteria inside cells, it requires considerable time for labeling. Phagocytes, including macrophages, can naturally respond to bacterial infection by activating numerous biological pathways, which make the macrophage microenvironment, including enzyme activity, redox state, and ROS levels, quite different from that of a healthy macrophage.^466^ This specific microenvironment can enable fast and bioresponsive intracellular bacteria labeling. As macrophages can recognize bacterial infections and activate caspase-1 (Casp-1),^467,468^ Liu et al. developed a molecular probe (PyTPE-CRP), an AIE molecule (PyTPE) conjugated with a casp1-responsive peptide (NEAYVHDAP), for bacterial infection imaging and intracellular bacteria elimination in macrophages.^469^ PyTPE-CRP exhibits weak fluorescence in aqueous media, but once it was selectively cleaved by Casp-1 in bacteria-infected macrophages, the resulting residues could self-assemble into aggregates and accumulate inside the phagosomes to light up the fluorescence in the macrophages. *In situ* intracellular bacteria ablation was achieved through the photodynamic treatment of PyTPE-CRP residues with negligible cytotoxicity to macrophages. This motivated the development of probes that could offer simultaneous activation of both fluorescence and photosensitization to facilitate real-time image-guided infection treatment with negligible side effects on normal cells. Bacteria with specific secretion systems are able to avoid the cytotoxic effect of hypochlorous acid (HClO), which is commonly released as a defense against bacterial invasion in phagocytes. Therefore, Liu and co-workers designed HClO-stimulated theranostic nanoprobe DTF-FFP NPs for bacteria diagnosis and therapy inside phagocytes (Figure 22B).^470^ DTF-FFP NPs are composed of two key elements: effective AIE PS DTF and HClO responsive molecules FFP, in which FFP quenched both the fluorescence signals and the ROS production of DTF by Förster resonance energy transfer (FRET). Upon accumulation of DTF-FFP NPs at the infected area, FFP was oxidized and degraded by the released HClO from the infected phagocytes, causing DTF to produce red fluorescence for intracellular bacteria imaging. Under light irradiation, the HClO-activated photosensitization selectively ablated bacteria and infected phagocytes without obvious side effects to normal phagocytes.

When compared to the first two methods, a simpler strategy is to design a probe that is permeable to cells and can offer direct bacteria labeling.^471^ Li et al. found that TBPs not only showed high efficacy against diverse G^+^ bacteria through membrane disruption without the development of resistance after repetitive passages for 30 days, but also killed the internalized pathogens via enhancing the autophagy of epithelial cells (Figure 22C).^472^ TBP-1 could directly bind to the bacterial phosphatidylglycerol and cardiolipin, thus inducing the loss of membrane integrity by increasing ROS accumulation and potential depolarization. Interestingly, after the infections of *S. aureus*, TBP-1 labeled bacteria colocalized with the lysosomes of epithelial cells, indicating that TBP-1 facilitated the clearance of intracellular bacteria in both pathogen-directed and host-directed ways. Notably, autophagy upregulated by TBP-1 was closely related to the combination of the PS with host mitochondria and subsequent ROS burst. Although mitochondria are the major energy supplier of host cells, lysosomes are directly involved in the elimination of intracellular pathogens.^473^ A D-π-A type AIEgen TTTh was reported with the capabilities to inhibit the growth of bacterial pathogens and to upregulate the lysosomal maturation at the same time (Figure 22D).^474^ Based on the cationic groups of the PS, TTTh bound to the methicillin-resistant G^+^ bacteria *S. aureus* (MRSA) within just 5 min. Then, TTTh directly impaired the membrane integrity of MRSA, and the damage was further aggravated after light irradiation, owing to a significant photodynamic effect. Meanwhile, lysosome-targeted TTTh also increased the lysosomal acidification and production of cathepsin D in macrophages, thus promoting the clearance of intracellular *S. aureus*.

## 4 Follow the Breadcrumbs: Detection and Monitoring

With the rapid development of medicine, life science research is moving from the macroscopic world to the micromolecular level. There are a wide variety of bioactive molecules with complex mechanisms in living organisms, such as amino acids, enzymes, and various species of active oxygen, nitrogen, sulfur, etc., which are an important part of the body’s homeostasis.^475−479^ Studying their physiology in-depth and visualizing these difficult-to-observe biological processes often require sophisticated tools. The saying “follow the breadcrumbs” from the story of Hansel and Gretel refers to tracing back clues or hints to find solutions, answers, or conclusions when the way forward is not clear. Fluorescence, to this extent, acts as the “breadcrumbs” that enable us to track invisible bioactive molecules or species in a real-time and *in situ* manner.^480−482^ In this section, we will discuss applications of AIEgens in detecting and monitoring biological molecules, including macromolecules and small molecules. Applications in various health-related areas, such as virus detection, food safety screening, and point-of-care testing, will be considered.

### 4.1 Biomolecules Detection and Monitoring

Biomolecules refer to bioactive small molecules, and biomacromolecules are involved in many physiological functions in the body and closely associated with a series of pathological events.^483,484^ For example, a variety of biological enzymes are widely regarded as biomarkers for the diagnosis and potential treatment of diseases in the organism.^485−487^ Meanwhile, gasotransmitters including nitric oxide, hydrogen sulfide, and carbon monoxide participate in various signaling pathways in biological systems such as regulating neurotransmission, relaxing blood vessels and inhibition of insulin signaling.^488,489^ In addition, the dynamic changes of intracellular cations and anions significantly affect the homeostatic control and lead to metabolic abnormalities.^490^ Thus, it is vital to obtain information *in situ* about the level change or dynamic process of these biomolecules for a deeper and more comprehensive understanding of disease diagnosis and treatment.

#### 4.1.1 Biomacromolecules Sensing and Screening

Biomacromolecules are large biological polymers composed of linked monomers, including nucleic acids, proteins, and carbohydrates. Biomacromolecules play critical roles in many biological processes, such as storage and transmission of genetic information and enzymatic catalysis of biochemical reactions, they also provide structural support to cells and tissues.^491^ Therefore, understanding the intricate structure and diverse functions of biomacromolecules is crucial for comprehending the complex mechanisms that underlie the behavior of living systems.

**Figure 23:**
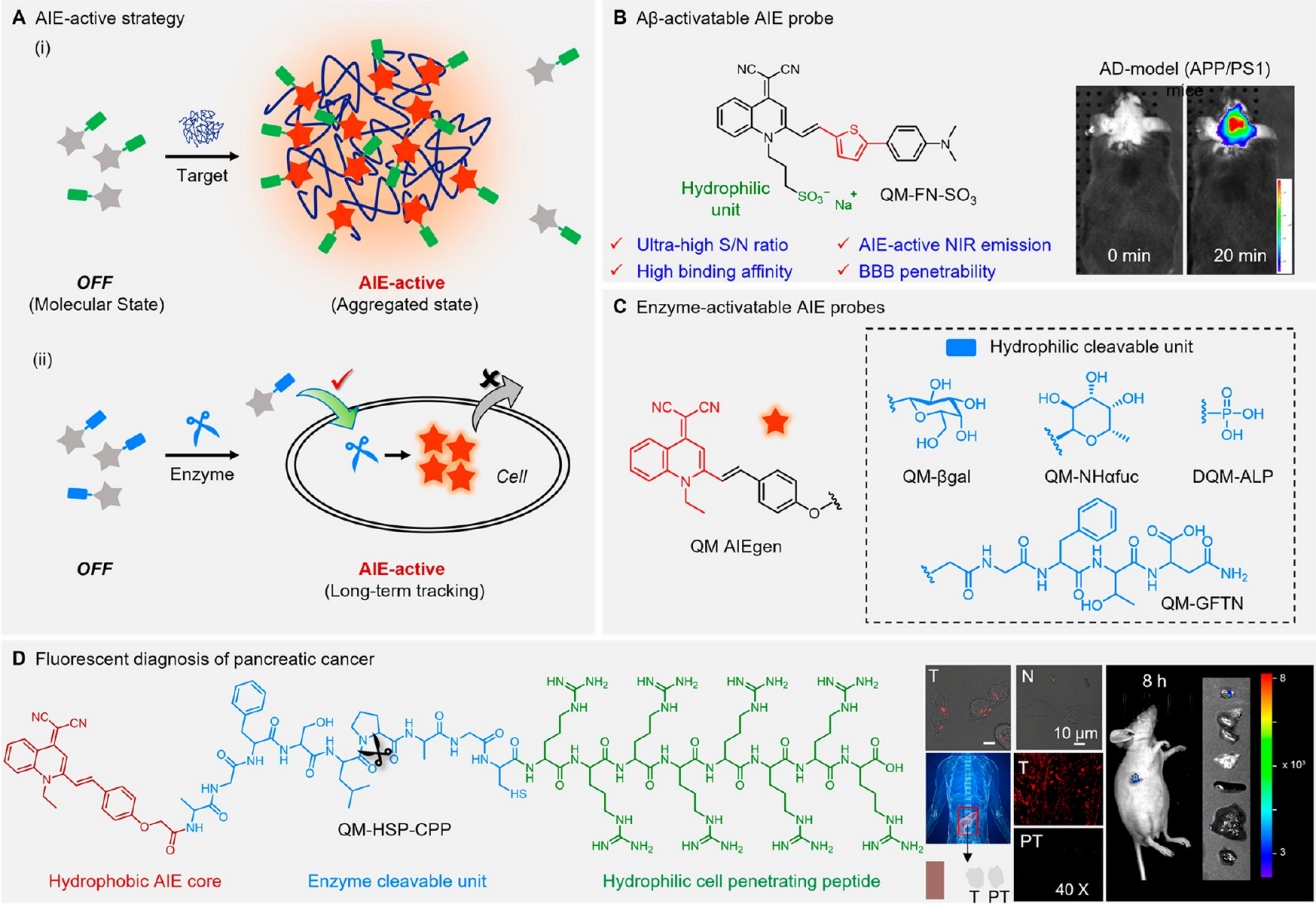
(A) AIE-active strategy. (B) QM-based Aβ probe QM-FN-SO_3_. Adapted with permission from ref (498). Copyright 2019 American Chemical Society. (C) Enzyme-activatable AIE probes: QM-βgal for β-galactosidase, QM-NHαfuc for α-l-fucosidase, DQM-ALP for hydrophilic alkaline phosphatase, and QM-GFTN for Atg4B protease. (D) QM-based probe QM-HSP-CPP for intraoperative pathological fluorescent diagnosis of pancreatic cancer via specific cathepsin E. Adapted with permission from ref (506). Copyright 2022 Wiley-VCH.

An AIE-active strategy is highly preferred to realize on-site and real-time monitoring of enzyme activities *in vivo*.^492,493^ However, because the hydrophobic aryl structure is an important structural motif in AIEgens, undesirable initial aggregation before sensing inevitably results in a false-positive fluorescence signal. Thus, increasing the water solubility of AIE probes is highly demanded, thus realizing a fluorescence-off background.^494−497^ Relying on the introduction of a hydrophilic sulfonic group to regulate the aggregated behavior, Zhu et al. built turn-on NIR probes for ultrasensitive mapping of Aβ plaques (Figure 23A and 23B).^498^ the fine structures of Aβ plaques were visualized using super-resolution imaging, and the 3D distribution of Aβ plaques was also determined.^499,500^ Moreover, given that the substrates of some crucial enzymes are naturally hydrophilic (such as glycosyl unit, peptide, etc.), conjugating AIEgens with a water-soluble substrate is a highly effective strategy (Figure 23C).^501^ In the molecular engineering of QM-βgal, hydrophobic QM-OH was utilized as an AIE reporter and the β-galactopyranoside unit was used as a hydrophilic cleavable substrate. QM-βgal is a highly water-soluble species, rendering it almost nonfluorescent in an aqueous solution. After being specifically hydrolyzed by β-galactosidase, hydrophobic AIEgens are released and aggregated, resulting in a lighting-up of the fluorescence signal. This sensing mode could also afford diffusion-resistant on-site localization and long-term trapping of endogenous β-galactosidase activity. Starting from the QM-βgal probe, a series of enzyme-activatable probes have been developed, and have enabled the *in vivo* sensing α-*L*-fucosidase, alkaline phosphatase, and Atg4B Protease, etc.^502−505^ Because pancreatic cancer is a highly lethal malignancy, a QM-based AIE probe (named QM-HSP-CPP) was used for the for high-fidelity fluorescence diagnosis of this kind of tumor by monitoring the overexpressed Cathepsin E (CTSE).^506^ QM-HSP-CPP is composed of three units: QM-OH as a long-wavelength AIEgen, peptide Ala-Gly-Phe-Ser-Leu-Pro-Ala-Lys-Arg (HSP) as the CTSE-responsive group, and transmembrane peptide for increasing water solubility and the deep-tissue penetration ability (Figure 23D). Notably, QM-HSP-CPP with amphiphilic characteristics well coped with the unwanted “always-on” fluorescence in the aqueous biosystem and the undesirable aggregation signal in the lipophilic organelle before targeting the receptor. Upon digestion with CTSE, the probe would aggregate into a tight state in an aqueous environment activating the AIE fluorescence signals. Consequently, QM-HSP-CPP was able to on-site monitor endogenous CTSE, thereby realizing pathological diagnosis of human pancreatic cancer sections and achieved long-term tracking in the heterotopic nude mouse model.

Protein aggregation is a multistep process that is associated with a growing number of human diseases, including neurodegenerative disorders, metabolic disorders, and cancers.^507−509^ Solvatochromic probes have been developed to reveal protein misfolding in both test tubes and living cells, as represented by Prodan, SBD, and others.^510−514^ In recent years, AIEgens have been applied to detect protein unfolding, oligomerization, and aggregation.^515−518^ 1,2-Bis[4-(3-sulfonatopropoxyl)phenyl]-1,2-dipheny-lethene salt (BSPOTPE), a biocompatible AIEgen, was used to report insulin amyloid genesis.^519^ BSPOTPE exhibits significant fluorescence activation that correlates with insulin nucleation, elongation, and equilibrium phases, thus enabling the assessment of the amyloid genesis kinetics. Most of the probes currently used, represented by Thioflavin T (ThT), can monitor only the mature fibrils, leaving the more critical oligomers undetected. Distinct from ThT, TPE-TPP exhibits a significant fluorescence enhancement and a shorter lag phase when monitoring α-synuclein fibrillation.^520,521^ Rapid fluorescence activation confirms that TPE-TPP could detect intermediate misfolded oligomers during fibrillation.^522^

**Figure 24:**
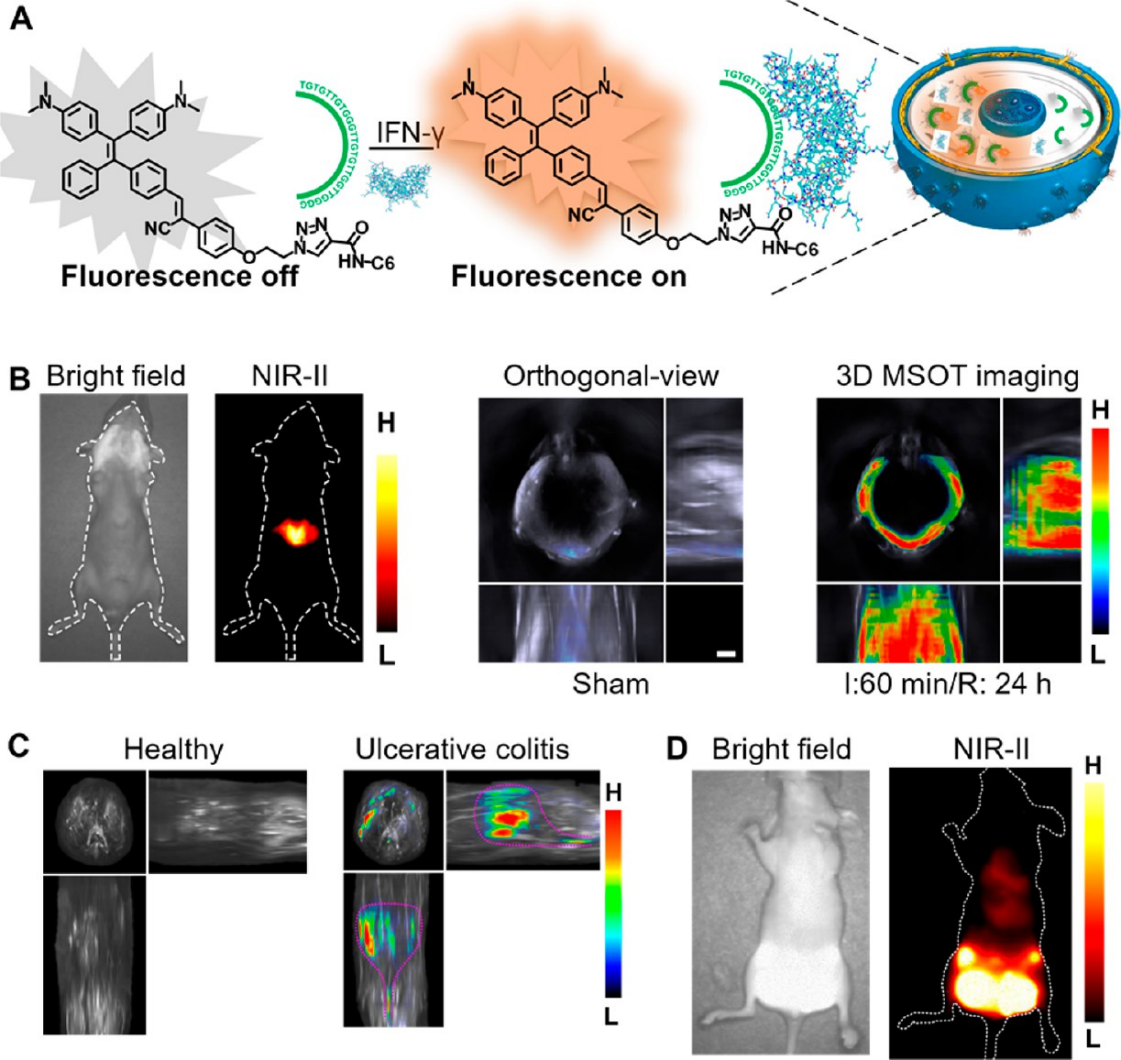
(A) Schematic illustration of the AIEgen-based fluorescent aptasensor for detecting intracellular IFN-γ. Adapted with permission from ref (524). Copyright 2018 American Chemical Society. (B) Bright-field and NIR-II fluorescent images and othogonal-view 3D MSOT images of liver ischemia-reperfusion injury in mouse model by the nanoprobe BTPE-NO_2_@F127. Adapted with permission under a Creative Commons CC BY License from ref (533). Copyright 2021 Springer Nature. (C) Orthogonal-view 3D MSOT images of ulcerative colitis by the nanosystem QM@EP. Adapted with permission from ref (534). Copyright 2022 Elsevier. (D) Bright-field and NIR-II fluorescent images of breast cancer metastasis in mouse model by the nanoprobe NP-Q-NO_2._ Adapted with permission from ref (535). Copyright 2020 Wiley-VCH.

Cytokines are small proteins that are released by cells; they act as important signaling molecules for cellular interactions, and changes in their concentration can reflect the health of the organism. Therefore, cytokines are often used as biomarkers of inflammation or diseases.^523^ The application of AIEgen-based immunoassays has made significant contributions to the field of cytokine monitoring. Ma et al. developed a turn-on fluorescence aptasensor that could monitor intracellular IFN-γ secretion (Figure 24A).^524^ This aptamer consisted of an AIEgen called TPEN_3_, which is a TPE molecule modified with an azide group and an IFN-γ aptamer. Only in the presence of IFN-γ, the TPE-aptamer complexes with IFN-γ and exhibits an intense red emission. This aptasensor was suitable for monitoring and real-time bioimaging of IFN-γ in living cells, with a minimum limit of detection (LOD) of 2 pg/mL. It was highly sensitive and specific as other proteins did not cause signal interference. Additionally, compared to “turn-off” counterparts, the “turn-on” character of AIEgens is advantageous for lowering the likelihood of producing erroneous positive/negative signals.^525^ Another AIEgen-labeled magnetic NPs was shown to localize cytokine VEGF.^526^ AIEgen-based poly (l-lactic-*co*-glycolic acid) Fe_3_O_4_ NPs modified with oleic acid were used as carriers, in combination with triphenylamine-divinylanthracene-dicyano and anti-VEGF antibodies so that they possessed specific immunoreactivity against VEGF, producing intense emission in the aggregate state and reacting to VEGF-A in cells down to 68 pg/mL.

Apart from the common applications of AIEgens as amyloid probes, their use as amyloid modulators and screeners has also been explored. Zheng et al. proposed a design strategy to develop a dual-function, multiple-targeting molecule of G7-TBA by conjugating a GNNQQNY (G7) amyloid fragment with a TBA AIEgen, which served as both an amyloid probe and amyloid modulator for monitoring, detecting, and altering amyloid aggregates.^527^ G7-TBA as an amyloid probe demonstrated its conformational-specific, sequence-independent detection ability with turn-on fluorescence for detecting Aβ, hIAPP (associated with Type 2 diabetes), and hCT (associated with medullary thyroid carcinoma) aggregates both in bulk and on the sensor surface. Meanwhile, G7-TBA as an amyloid modulator demonstrated its multiple-target functions to (i) accelerate the aggregation and misfolding of different Aβ, hIAPP and hCT, (ii) reduce amyloid-induced cell toxicity, and (iii) generate fluorescence images of amyloid fibrils. Such sequence-independent amyloid probing and modulation mechanisms of G7-TBA mainly stem from their cross-interactions with amyloid detection via β-structure interactions. This study offered a “kill two birds with one stone” case model to not only integrate both “amyloid probe/sensing” and “amyloid modulation” functions into a single molecule but also to introduce the concept of amyloid cross-seeding for designing protein-based, AIE-active molecules for disease diagnosis and drug discovery. In another case, a class of amyloid inhibitor probes was developed by conjugating EPB molecules with pAzF-modified amyloid proteins (i.e., EPB@pAzF-amyloid probes), realizing the high-throughput screening of small molecules as amyloid inhibitors.^528^ Upon incorporation of pAzF and EPB into a specific site of Aβ42 or αSN proteins, EPB@pAzF-amyloids were still able to self-aggregate, changing from unstructured monomers to semistructured oligomers to β-sheet-rich amyloid-like fibrils, and showed similar amyloidosis properties to wild-type amyloids. These EPB@pAzF-amyloid conjugates served as a high-throughput screening platform to repurpose FDA-approved drugs, small molecules, and natural components with amyloid inhibition functions against different amyloid proteins. This strategy discovered a dual amyloid inhibitor of tolcapone from Drugbank databases to prevent the aggregation, cell toxicity, and neuronal dysfunction of both Aβ and α-synuclein. Molecular modeling and molecular dynamics simulations further revealed that the common inhibition effect of tolcapone on Aβ and α-synuclein aggregation stemmed from the strong binding of tolcapone molecules to a common structural motif of β-sheet grooves present in both Aβ and α-synuclein oligomers as well as to some hydrophobic, aromatic residues, which explained the experimental data.

#### 4.1.2 Biological Small Molecules Detection and Monitoring

Besides biomacromolecules, many small biomolecules also play important roles in complex biological processes.^87,529^ Multispectral optoacoustic tomography (MSOT), which relies on the use of multiple-wavelength NIR laser excitation and spectral unmixing algorithms, can distinguish optoacoustic signals contributed by different photoabsorbers and can identify a specific probe in the target tissue.^530^ Additionally, MSOT imaging, which can generate 3D images (maximum-intensity projection images or 3D rendering images), can detect spatially overexpressed biomarkers in the organ or tissue of interest.^531,532^ Therefore, the combination of NIR-II imaging and MSOT imaging with a biomarker-activatable probe can combine their respective advantages and establish a serviceable means for offering accurate and noninvasive diagnosis and therapeutic outcome monitoring with high spatiotemporal resolution.

One typical example is the nanoprobe BTPE-NO_2_@F127, realized by molecular probe BTPE-NO_2_ encapsulated with the FDA-approved amphiphilic and biocompatible polymer Pluronic F127.^533^ The biomarker H_2_O_2_ at pathological levels in the disease sites (e.g., in the liver) cleaved the amide bond of the electron-withdrawing biomarker-responsive nitrophenyloxoacetamide units and produced the activated probe (chromophore BTPE-NH_2_) with the electron-donating amino groups, thereby red-shifting the absorption band to 680–850 nm and generating strong NIR-II emission in the range 950–1200 nm. With two TPE groups in the core serving as the molecular rotors, the activated probe (chromophore BTPE-NH_2_) was AIE-active, further enhancing the NIR-II fluorescence in the aggregate state. The nanoprobe was used to image liver ischemia reperfusion (I/R) injury, which is a major complication in clinical scenarios such as liver resection and liver transplantation. In the case of liver I/R injury, ROS (including H_2_O_2_) is overexpressed in the hepatic region, which could serve as a biomarker for the disease. The nanoprobe BTPE-NO_2_@F127 could detect the degree of liver injury, on account of its response to hepatic H_2_O_2_. Mice were treated with ischemia for 0 min (the sham group serving as the control), 30 or 60 min, followed by reperfusion for 24 h, then the mice were intravenously (i.v.) injected with the nanoprobe and underwent imaging. As shown in Figure 24B, the liver area of the mice in the sham group only exhibited weak fluorescence and optoacoustic signals at 90 or 120 min upon i.v. injection of the nanoprobe BTPE-NO_2_@F127, while the liver areas of the ischemia groups exhibited obvious fluorescence, and the long ischemia group (ischemia for 60 min) exhibited stronger fluorescence and optoacoustic intensities, because a higher level of hepatic H_2_O_2_ was generated for extended ischemia time and thus more severe liver damages occurred for the long ischemia group. The AIE-active hemicyanine-based molecular probe QY-SN-H_2_O_2_ and the NLRP3 inhibitor MCC950 encapsulated using enteric polymers rendered the colon-targeting nanosystem QM@EP, those enteric polymers were FDA-approved PLGA and Eudragit S100, with the former being used as a biodegradable excipient for sustained release of drugs, and the latter as the excipient for targeted payload release in the colon since Eudragit S100 is soluble in colon-fluid and usually serves as a matrix for drug formulation to prevent premature drug release in the stomach and specifically release drug in colon at pH > 7.^534^ Once triggered by colonic pH, the nanosystem discomposes and subsequently releases the molecular probe and NLRP3 inhibitor in the colon. Accordingly, the released NLRP3 inhibitor exerts a therapeutic effect, and the pathological colonic H_2_O_2_ cleaves the biomarker-responsive moieties of QY-SN-H_2_O_2_ to generate the AIE-active chromophore QY-SN-OH for NIR-II fluorescence imaging and optoacoustic imaging (Figure 24C). Hence, this nanosystem served as a theranostic tool for ulcerative colitis integrating noninvasive *in situ* imaging and therapy into one system. Another AIE-active hemicyanine chromophore (Q-NO_2_) readily generates the nanoprobe (NP-Q-NO_2_) in aqueous media due to its amphiphilic molecular structure, which is responsive to the biomarker nitroreductase. In the absence of nitroreductase, the nanoprobe was neither absorptive nor fluorescent in the NIR region.^535^ Nitroreductase transformed the strongly electron-withdrawing nitro group in the molecular probe to an amino group, which led to a self-elimination reaction and generated the electron-donating hydroxyl group, thus offering an activated probe (NP-Q-OH) with strong optoacoustic and NIR-II fluorescence signals. NP-Q-NO_2_ was shown to respond to nitroreductase in a breast tumor metastasis mouse model, and image the sequential metastases from the orthotopic breast tumors to lymph nodes and then to the lung (Figure 24D).

### 4.2 Virus Detection

Viruses typically range between 20 and 300 nm in length. An emerging and re-emerging spectrum of viral infectious pathogens has severely threatened human health and become one of the major public health concerns. Facing the continuous and severe threat of COVID-19, it became critical for mankind sensitive and accurate methods for virus clinical diagnosis, and especially for early stage of infection, to prevent the spread of viruses and disease outbreaks.^536,537^

**Figure 25:**
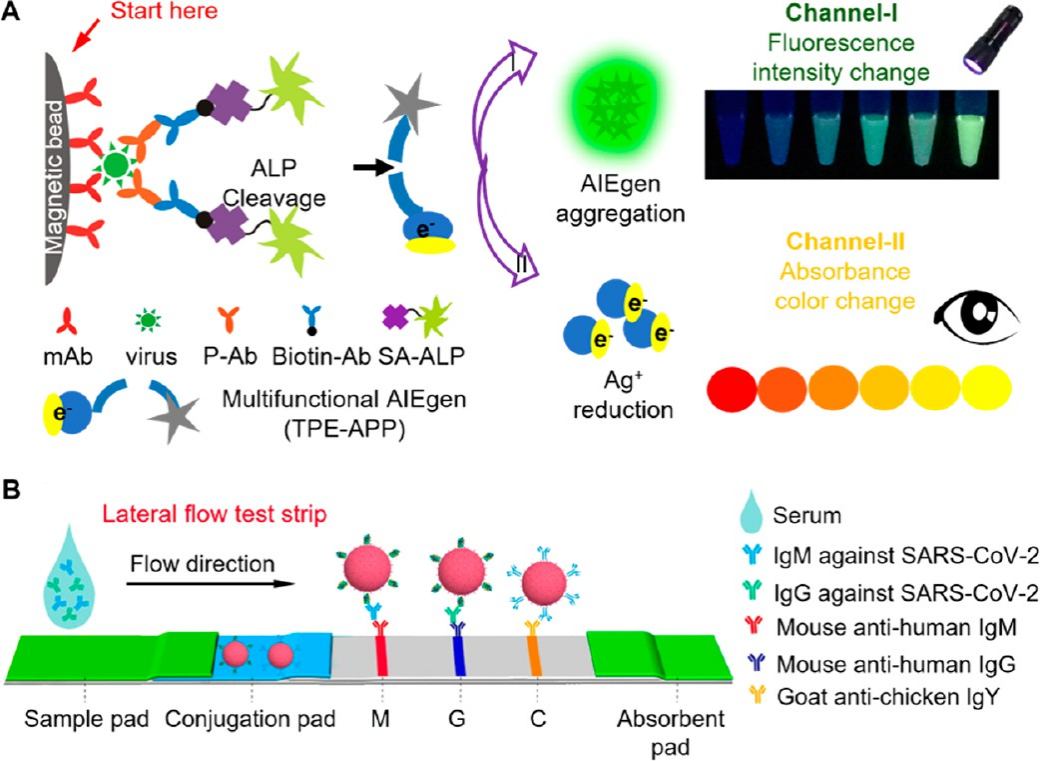
(A) Schematic illustration of the fluorescent and plasmonic colorimetric dual modality for virus detection. Adapted with permission from ref (538). Copyright 2018 American Chemical Society. (B) Schematic illustration of the test strip developed for the detection of IgM and IgG against SARS-CoV-2 in a human serum sample. Adapted with permission from ref (540). Copyright 2021 American Chemical Society.

A dual-modality immunoassay for viruses was developed based on an enzymatic-responsive TPE-based AIEgen (Figure 25A).^538^ Initiated by virions immunobridged enzymatic hydrolysis of terminal phosphoryl groups, highly emissive AIE aggregates and silver-shelled gold NPs were generated, leading to a robust turn-on fluorescence and naked-eye discernible plasmonic colorimetric signal, respectively. As a proof of concept, EV71 virions could be specifically detected with a detection limit as low as 1.4 copies/μL under fluorescence modality. Additionally, the naked eye could observe a broad range of EV71 virions from 1.3 × 10^3^ to 2.5 × 10^6^ copies/μL. Most importantly, real clinical samples were diagnosed with 100% accuracy using RT-qPCR as a standard, without the assistance of any expensive instrumentations. This dual-modality immunoassay has enabled convenient preliminary screening based on a colorimetric signal and the accurate diagnosis of suspect infections via a fluorescence signal.

Considering the imaging advantages of NIR emission, significant effort has been devoted to developing NIR AIEgens for the detection of severe acute respiratory syndrome coronavirus 2 (SARS-CoV-2).^539,540^ For the early detection of immunoglobulin M (IgM) and immunoglobulin G (IgG) in clinical serum samples, Li and colleagues developed a rapid and sensitive serological diagnostic method based on AIE_810_NP in a lateral flow immunoassay (Figure 25B).^540^ The AIEgen, BPBT, with NIR emission at 810 nm, served as the fluorescent unit. The detection ligand was labeled with polystyrene NPs sized 300 nm, which were loaded with 3.18 × 10^6^ AIE_810_NP to amplify the fluorescent signal. Compared to the enzyme-linked immunosorbent assay and the AuNP-based test strip, the AIE_810_NP exhibited a lower LOD (0.236 μg mL^–1^ and 0.125 μg mL^–1^) and comparable or higher sensitivity (78% and 95%) for IgM and IgG. Such effective and accurate detection of COVID-19 could help to cut the spread of the virus and halt the pandemic in the public area.

### 4.3 Food Safety

Food safety is a major issue associated with national health and social stability, which has attracted increasing attention with the transformation of the human diet from traditionally eating until full (quantity) to eating well (quality).^541,542^ Generally, food safety problems can be caused by microbial contamination, environmental organic pollutants, food spoilage, and illegal use of pesticides, veterinary drugs, fertilizers, and food additives.^543,544^ Food safety inspection is regarded as an important means of monitoring food quality and ensuring food safety. In recent years, several analytical techniques have been developed to test and monitor food quality and safety. In this regard, fluorescence detection technology based on AIEgens has the advantages of high sensitivity, simple operation and user-friendliness, which exhibits significant prospects in the area of practical food safety and quality control.^545−547^ According to the RIM mechanism, the fluorescence signal switching of AIEgens can be easily achieved by activating/inactivating RIM processes or disrupting the photophysical processes through target-assisted molecular recognition.^370^ Accordingly, various high-performance fluorescence sensing detection systems have been developed for food safety and quality monitoring.

**Figure 26:**
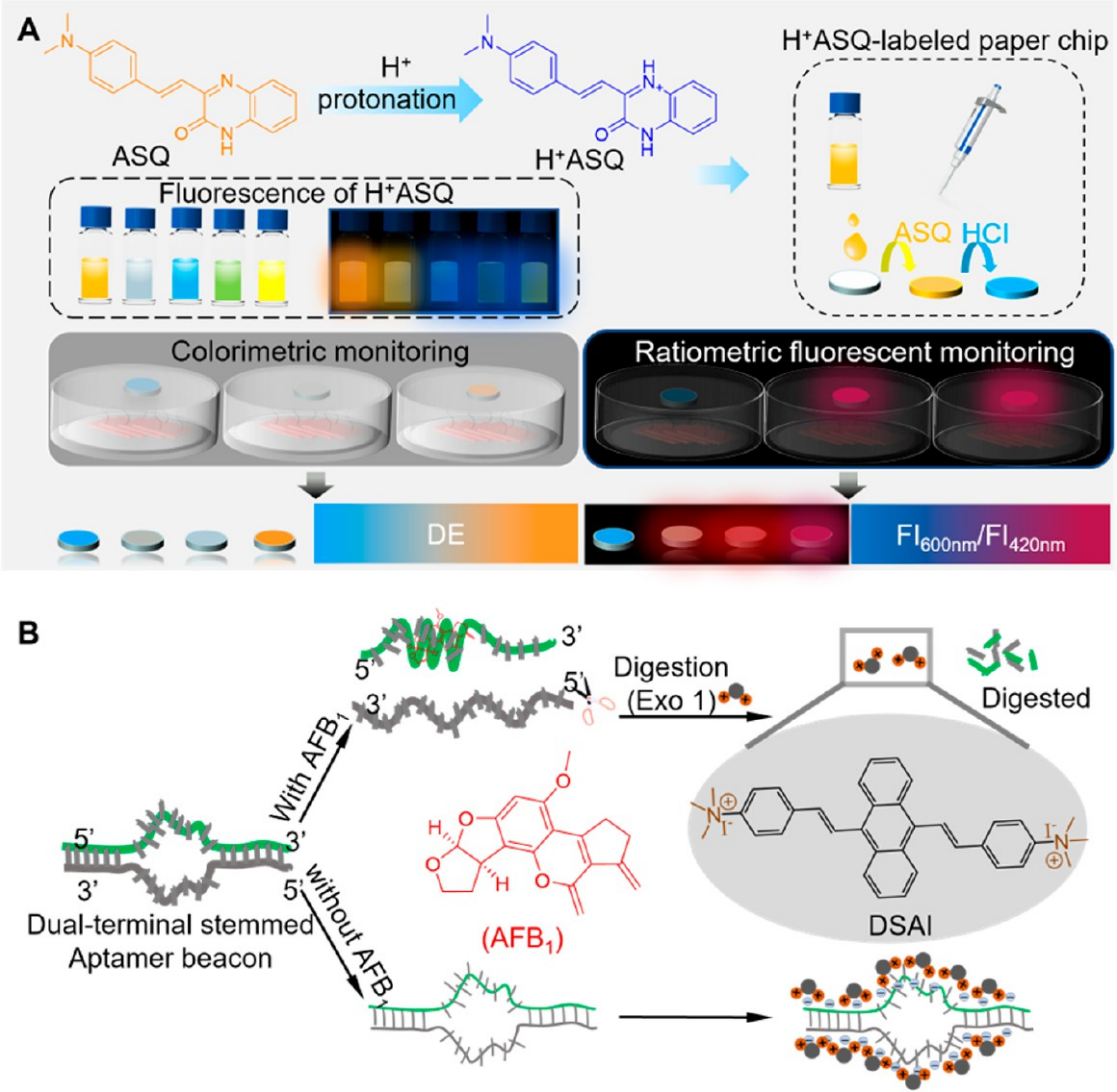
(A) Schematic illustration of preparation of a H^+^ASQ-loaded paper chip and their use in real-time and visual biogenic amine monitoring. Adapted with permission from ref (548). Copyright 2022 Elsevier. (B) The design of an aptamer beacon and its application for the label-free detection of AFB_1_. Adapted with permission from ref (557). Copyright 2018 American Chemical Society.

Active substances in food can interact directly with AIEgens by various chemical reactions such as coordination, hydrogen bonding, and hydrophobic and electrostatic interactions, thus achieving precise manipulation of the photophysical process occurring in AIEgens and thus controllable fluorescence signaling. Such target-directed responsive AIEgens do not require any complex design or operational processes. A typical example is from AIEgens with amine-response activities, which were used to monitor food freshness. Huang, Xiong, and co-workers reported an amine-responsive AIEgens, namely, 4-(dimethylamino)styryl)quinoxalin-2(1*H*)-one (ASQ), which was used as biogenic amine indicator to develop an intelligent sensor chips for monitoring food freshness (Figure 26A).^548^ ASQ, with a typical D-A structure, displayed nonmonotonic color and fluorescence responses to pH by protonation/deprotonation of the D/A groups. After depositing acidified ASQ (H^+^ASQ) on qualitative filter paper, the obtained paper sensor chip showed obvious colorimetric and ratiometric fluorescence responses to biogenic amines. Due to its excellent amine reactivity, the H^+^ASQ-labeled paper chip enabled real-time, nondestructive, and visual monitoring of meat and seafood freshness at different storage temperatures. Additionally, AIEgens with amine-, H_2_S-, Al^3+^-, and O_2_-reactivity have been reported for monitoring the freshness of fruits, red wine, crab, fish, shrimp, and scallops.^549−556^

Although the above-mentioned target-responsive AIEgens can enable the real-time *in situ* monitoring of food hazards, the sensitivity and accuracy of such AIEgen systems face great challenges due to the diversity of food hazards and the complexity of foods. Therefore, design concepts and strategies for AIEgens targeting various food hazards are urgently required. In this regard, indirect-responsive AIEgens have been developed as promising alternatives, in which targets can react with the intermediate and induce changes in its physicochemical properties, which in turn trigger the aggregation/disaggregation of AIEgens, or the detachment/release of AIEgens from the quenchers/activators. Deng et al. designed a dual terminal stemmed aptamer beacon (DS aptamer beacon) for homogeneous and label-free detection of aflatoxin B_1_ (AFB_1_) in broad bean sauce and peanut oil (Figure 26B).^557^ With this research, the DS aptamer beacon possessed two stems at the 3′ and 5′ terminals and two symmetrical loops in the middle region. The stems at the 3′ and 5′ terminals could protect the aptamer probe from being digested by exonuclease I. When AFB_1_ was present in the sample solution, the target would competitively bind to the aptamer and induce disassembly of the DS aptamer beacons. With the beacon structure switching, the generated single-stranded DNA was subsequently digested by exonuclease I, thus inhibiting the binding of positively charged AIEgen (DSAI) and resulting in a decreased fluorescent signal. The LOD of this DS aptamer beacon-driven AIEgen assay was as low as 27.3 ng/mL.

Organophosphorus pesticides (OPs) are widely used to improve the agricultural production efficiency and product quality. However, OPs can irreversibly inhibit acetylcholinesterase (AChE) activity even at low concentrations, thus causing excessive accumulation of the neurotransmitter acetylcholine (ATCh) in living organisms. ATCH is associated with many neurological diseases. Thus, developing highly sensitive methods for the quantitative detection of OPs residues is of great importance. In this regard, several indirect-responsive label-free AIEgens toward OPs have been developed by the AChE-mediated catalytic hydrolysis of ATCh to manipulate the aggregation or release of AIEgens, resulting in significantly enhanced fluorescence.^558^ Using this strategy, Li et al. achieved the rapid visual detection of AChE activity and OPs with LODs of 2.5 mU/mL and 0.5 ng/mL, respectively, using a paper-based fluorescence sensor based on maleimide functionalized TPE as an indicator.^559^ Furthermore, a 3D-printed fluorescent sensing platform was developed by Jiao et al. to detect OP levels in vegetables quickly.^560^ The AIEgen maleimide-functionalized TPE molecule was nonemissive in both solution and solid states, but could be easily illuminated by thiol-ene hydrothiolation reaction. Under alkaline conditions, OPs were hydrolyzed to thiols and reacted with TPE-MI to form a fluorescent thiolated product. Strong emission of the solution was captured and analyzed by a smartphone, and the OPs residue concentration exhibited a linear relationship with the smartphone readout gray value, with the LOD of 0.01–0.5 mg/kg. The analysis time was decreased to 30 min, making the detection of OPs residual concentrations more rapid, sensitive, and portable. Such portable sensing devices were realized with the superior selectivity, excellent photostability, and outstanding signal reliability of AIEgens.

### 4.4 Point-of-Care Testing

In recent years, the use of point-of-care testing (POCT) methods for disease diagnosis and bioassays has attracted widespread interest.^561^ The combination of AIEgens with diverse analytical platforms to develop portable and efficient POCT detection devices exhibits significant potential in addition to a promising market value.

**Figure 27:**
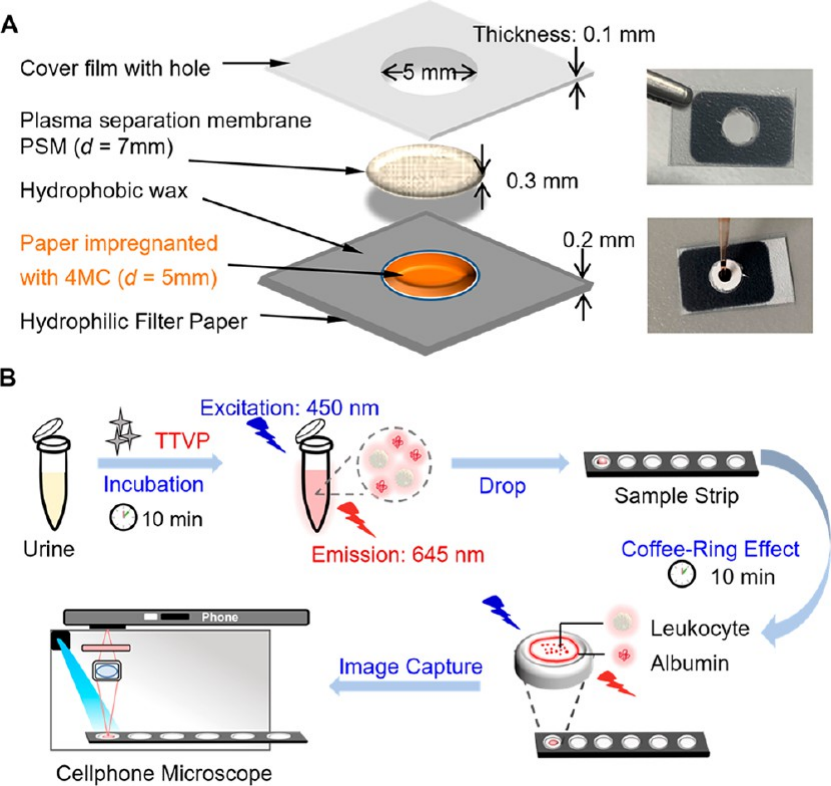
(A) Schematic of the 4MC-integrated microfluidic paper-based analytical device (4MC-μPAD). Adapted with permission from ref (563). Copyright 2020 Wiley-VCH. (B) Schematic assay protocol and detection setup for the AIE-based POCT method for one drop of urine. Adapted with permission from ref (565). Copyright 2022 American Chemical Society.

As a biomarker for many specific diseases, home monitoring of human serum albumin (HSA) concentration is particularly critical for elderly and chronic disease groups.^562^ For the rapid detection of HSA, a microfluidic paper-based analytical device (μPAD) that was based on a ratiometric fluorescence method was demonstrated (Figure 27A).^563^ Change from 4MC nanoaggregates to the 4MC-HSA complex resulted in a color change from red to green which was distinguishable to the naked eyes. A linear response toward HSA concentrations over a range from 0 to 9 μM, with a calculated LOD of 16.4 nM and a response time of <3 min was obtained. By integrating 4MC with the cost-effective paper-based platform μPAD, high precision ratiometric detection of HSA in blood samples could be achieved, and the needs for their rapid detection at home could be met.

Urine albumin detection is recommended by multiple clinical practice guidelines for early chronic kidney disease screening.^564^ Leukocytes in urine can reflect the occurrence of a body infection or inflammation. Situ, Zheng, and co-workers reported an AIE-based POCT method for rapidly detecting and quantifying microalbuminuria and leukocytes using one drop of urine.^565^ The AIE probe TTVP activated its NIR fluorescence in the presence of albumin and leukocyte via hydrophobic or electrostatic interactions. In combination with a smartphone-based detection device, the fluorescence signals were well-separated. This was because after evaporation, due to the coffee ring effect, the urine’s albumin in the droplets was driven and concentrated toward the contact line, while most of the leukocytes were evenly distributed on the contact surface, dominated by gravitational sedimentation. Thereby, simultaneous quantification and analysis of urine’s albumin and leukocytes was successfully achieved within 20 min (Figure 27B). This study provides a rapid, simple, and low-cost approach for urinary POCT analysis. Also, an open platform was designed by Tang and co-workers, which was capable of providing suitable optical excitation for different fluorescence processes, consisting of a flexible optical signal detector, either wired or wireless connection for transferring and storing data, as well as a single computer module to analyze the fluorescence signal locally.^566−568^ This portable device based on colorimetry was able to detect and monitor albumin, glucose, and creatinine by loading 96-well plates, cuvettes, basements, etc., and with imaging capture and built-in analyzing algorithms to evaluate the Red Green Blue (RGB) color-space values. Ultimately, urinary measurements of the 73 patients with renal diseases using this device were in good agreement with the local clinical results.

Metal-AIEgen frameworks (MAFs), which refer to AIEgens employed as ligands of MOFs, are bright fluorescent materials, with PLQY reaching 99.9%.^569^ Jiang et al. reported 1,1,2,2-tetra(4-carboxylphenyl)ethylene (TCPE)@Zr MAFs and their application in POCT sensors.^570^ By adding regulators, blue-emissive MAFs were synthesized with different morphologies and sizes, and transparent MAFs@hydrogel composites for stable and ultrasensitive POCT were produced. Compared to the other fluorescent MOFs, the sensitivity of MAFs was enhanced about 1000 times. The authors further explored MAF particles as fluorescent tags for immunodetections, showing a strong binding affinity to label antibodies through simple direct incubation. Due to the high PLQY luminescence efficiency of MAFs particles, LODs of MAFs-based lateral flow immunoassays were lower than conventional QDs-based ones. Therefore, MAFs can be considered as promising next-generation functional materials for POCT sensors.

## 5 Summary and Perspective

Emerging from being a seemingly paradoxical photophysical phenomenon, AIE has grown into a vibrant research field over the last two decades due to numerous groundbreaking and trailblazing advancements. While initially focused on fluorescence, the AIE has expanded well beyond this domain. Deepening understanding of the RIM mechanism has endowed AIEgens with additional attractive merits, including enhanced ROS generation ability, photothermal/photoacoustic effects, room temperature phosphorescence, circularly polarized luminescence, etc., offering diverse application prospects for bioimaging, sensing, and therapy. Owing to the great efforts made by scientists worldwide, AIE has also flourished in the areas of life science and healthcare, with collaborations spanning clinical diagnosis, drug delivery, pathology, rehabilitation, and precision medicine. As summarized in this review, the study topics for AIE have been expanded from *in vitro* proteins and organelles to tissues and living animals. Also, apart from monofluorescence imaging, imaging techniques were significantly expanded to super-resolution, two-photon, NIR, and afterglow imaging. Treatment options have progressed from monotherapy to synergistic therapy, which combines phototherapy (PDT/PTT) with chemotherapy, immunotherapy, or gene therapy.

Up to now, a plethora of AIE materials have been prepared in different laboratories, but the issues of high-cost and time-consuming synthetic routes, environmental concerns from the synthetic protocols, poor hydrophilicity, and potential dark toxicity of PSs are still inevitable. In this regard, natural AIEgens derived from biomass sources, with intrinsic advantages of abundance, sustainability, biodegradability and pharmacological activity, may offer a promising alternative to conventional artificial AIEgens.^571^ Such natural AIEgens can be divided into nonaromatic and aromatic structures.^572,573^ Nonaromatic biomass sources for natural AIEgens are primarily composed of polysaccharides such as cellulose, hemicellulose, sodium alginate, starch, and chitosan, as well as monosaccharides including glucose, xylose, fructose, and galactose.^574^ Early reports on the emission behavior of these materials go back to the observation of fluorescence and phosphorescence in solid powders of starch and cellulose.^575^ These natural luminophores generally possess nonconjugated structures containing subgroups, such as ether, hydroxyl, carbonyl, and carboxyl, and tend to form strong hydrogen-bonding interactions with each other.^576^ As a result, these oxygen-incorporating units form clusters in concentrated solutions or the solid state. In contrast to through-bond conjugation of aromatic structures, through-space conjugation occurs in the oxygen clusters.^577^ Aromatic natural AIEgens are primarily derived from the secondary metabolites of plants, and reported aromatic AIEgens to include lignin, quercetin, berberine chloride, tannic acid, riboflavin, palmatine, myricetin, etc.^578−580^ Recent studies indicated that aromatic AIEgens, such as lignin, tannin acid and caffeic acid, could exhibit RTP when trapped in a polymer/inorganic matrix.^581−583^ While many natural AIEgens have been reported in recent years, they represent only a small fraction of the vast library of natural products. We believe that there are still a large number of AIE-active natural products waiting to be discovered and explored. In addition to studying their luminescence properties, further investigations into pharmacological mechanisms as well as applications in diagnosis and therapy are desired.

Based on this review, we envision some critical issues in biological applications that need to be carefully considered:

For improving background signal in bioimaging, further efforts should be focused on NIR-II imaging, multiphoton fluorescence microscopy, and afterglow luminescence. Bioimaging in the NIR-II window (1000–1700 nm), and especially the NIR-IIb window (1500–1700 nm), enables deeper tissue penetration and higher spatial resolution, thus providing a high signal-to-noise ratio. However, currently available NIR-IIb AIEgens still lag behind their NIR-IIa counterparts and suffer from a relatively low brightness. To address the limitations of the current NIR-IIb AIE agents, novel systems with a high PLQY in the NIR-IIb region and a large absorption coefficient at the wavelength of the laser excitation should be developed. Furthermore, combinations of hybrid nanomaterials, such as AIEgens coupled with upconversion NPs, can enable both excitation and emission in the NIR region. Multiphoton fluorescence microscopy offers deeper penetration depth and better optical focusing due to much lower light scattering, resulting in an ultrahigh signal-to-background ratio and better sectioning ability as compared to the traditional single-photon microscopy. The confined imaging area is the main limitation of multiphoton fluorescence microscopy, particularly three-photon excited emission. Afterglow imaging has emerged as an alternative to NIR-II imaging or multiphoton fluorescence microscopy as it enables satisfactory background signals with high contrast as well as long-time monitoring. However, afterglow luminescence is relatively weak compared to fluorescence, and its intensity becomes even weaker over time. Therefore, efforts should focus on both enhancing the PLQY and prolonging the lifetimes of AIE-active afterglow agents. Another issue to address is the stability of afterglow emission in complex biological environments, where it can be easily quenched (e.g., by singlet oxygen). More importantly, multimodal imaging combining the above-mentioned or other imaging techniques is a feasible approach to improve the quality of biomedical imaging.

The translation of AIE materials from the laboratory to the clinical environment still faces several challenges that need to be addressed. Interference by the dynamic and complex environment of *in vivo* diagnosis and treatment, AIEgens may undergo different chemical reactions and physical changes caused by unknown species, such as oxidation, fluorescence quenching, and unexpected aggregation or disaggregation. Thus, the challenge is to maintain the high specificity and sensitivity of AIEgens in real samples. In addition, maintaining the photostability of AIE agents in complex biological systems for *in vivo* long-term monitoring, such as in cases of tumor metastasis and organ transplantation, is crucial. Biosafety, especially the long-term safety of AIEgens, is another significant concern in the clinical translation process. Despite abundant reports indicating the biocompatibility of AIEgens, it is essential to conduct further research into the pharmacokinetics and metabolism of AIEgens via established long-term toxicological evaluations. Moreover, the translation of AIE materials from the laboratory to the clinic necessitates regulatory approval from relevant authorities such as the Food and Drug Administration (FDA). This process involves extensive documentation and validation of the safety, efficacy, and quality of AIE materials, as well as compliance with various regulatory requirements and guidelines.

With the advent of big data, artificial intelligence (AI) has provided opportunities for advancing research in the AIE field. In this regard, a universal and versatile database on aggregate science research named ASBase has been built.^584^ One of the areas where AI can be used in the design and synthesis of AIE-active molecules. By analyzing the large data sets of chemical structures, and photophysical and physicochemical properties of AIE compounds in different aggregate states, AI algorithms can quickly screen potential candidates and predict their performance. It is expected that this will significantly accelerate the discovery of novel AIEgens and reduce time-consuming and high-cost synthesis and characterizations. Big data can also enable real-time data analysis and interpretation, allowing for more accurate and reliable detection and monitoring. Thus, we anticipate that AI-assisted research will become a vital aspect of AIE research.

AIE materials have already shown promising potential in the fields of health and life science. Moving forward, we firmly believe that AIE can continue to take the central stage at the forefront of life science, particularly in the areas of neurodegenerative diseases, phase separation, cell communication, and optogenetics. To that end, significant efforts are to invest in AIE research, driving innovation and progress to improve our life and health. With ongoing exploration and development, we expect that AIE will make revolutionary discoveries and create innovative methods toward understanding biological systems and improving human health.

## VOCABULARY

luminogens: those nonemissive as molecules but emissive under appropriate conditions, e.g., as aggregates
AIE luminogens (AIEgens): nonemissive or weakly emissive in their single molecular state but strongly emissive in the aggregate state
Stokes shift: the difference between the spectral position of the maximum of the first absorption band and the maximum of the fluorescence emission and can be expressed in either wavelength or wavenumber units
precision medicine: sometimes known as “personalized medicine”, is an innovative approach to tailoring disease prevention and treatment that takes into account differences in people’s genes, environments, and lifestyles
theranostics: the integration of diagnosis and therapy in a single platform
limit of detection (LOD): the lowest concentration that can be measured/detected with statistical significance by means of a given analytical procedure
